# An ultra-tunable platform for molecular engineering of high-performance crystalline porous materials

**DOI:** 10.1038/ncomms13645

**Published:** 2016-12-07

**Authors:** Quan-Guo Zhai, Xianhui Bu, Chengyu Mao, Xiang Zhao, Luke Daemen, Yongqiang Cheng, Anibal J. Ramirez-Cuesta, Pingyun Feng

**Affiliations:** 1 Department of Chemistry, University of California, Riverside, California 92521, USA; 2 Department of Chemistry and Biochemistry, California State University Long Beach, 1250 Bellflower Blvd, Long Beach, California 90840, USA; 3 Spallation Neutron Source, MS-6473, Oak Ridge National Laboratory, Oak Ridge, Tennessee 37831, USA

## Abstract

Metal-organic frameworks are a class of crystalline porous materials with potential applications in catalysis, gas separation and storage, and so on. Of great importance is the development of innovative synthetic strategies to optimize porosity, composition and functionality to target specific applications. Here we show a platform for the development of metal-organic materials and control of their gas sorption properties. This platform can accommodate a large variety of organic ligands and homo- or hetero-metallic clusters, which allows for extraordinary tunability in gas sorption properties. Even without any strong binding sites, most members of this platform exhibit high gas uptake capacity. The high capacity is accomplished with an isosteric heat of adsorption as low as 20 kJ mol^−1^ for carbon dioxide, which could bring a distinct economic advantage because of the significantly reduced energy consumption for activation and regeneration of adsorbents.

## Abstract (web summary)

Synthetic design of crystalline porous materials is important for applications such as catalysis and adsorption. Here, the authors demonstrate a platform for the development of crystalline porous materials with a variety of organic ligands and metallic clusters, and control of their gas sorption properties.

The development of high-performance CO_2_ capture materials is important for efficient and cost-effective carbon capture and storage. Towards this goal, many materials have been studied for their CO_2_ capture properties. Among them, crystalline porous materials (CPM), metal-organic framework (MOF) materials in particular, have risen to a prominent position, because their compositional and structural variety lends them a great potential for property engineering^1^,^2^,^3^,^4^,^5^,^6^,^7^,^8^,^9^,^10^,^11^,^12^,^13^,^14^,^15^,^16^,^17^,^18^,^19^,^20^,^21^,^22^,^23^,^24^,^25^. Currently, the best-performing materials, in terms of CO_2_ uptake capacity, belong to the MOF-74 family made from 2,5-dihydroxy-1,4-benzenedicarboxylic acid (here denoted as H_2_DHBDC or H_4_DOBDC, depending on whether it is −2 or −4 in resulting MOFs) and various metal ions (usually Mg, Co, Ni and Zn)^26^. Specifically, MOF-74-Mg has the highest CO_2_ uptake capacity (slightly under 230 cm^3^ g^−1^ at 273 K and 1 bar)^27^ among known porous solids.

While the MOF-74 structure type has highly desirable features such as high-density open-metal sites, as a platform for property engineering, it has significant limitations with respect to its compositional tunability. For example, the bonding mode between M^2+^ and DOBDC^4−^ in MOF-74 is very unique in MOF chemistry and is inaccessible by common ligands. It is therefore desirable to develop an alternative platform with greater tunability for engineering materials' properties.

Before our study, while many MOF structure types are known and some of them do have far greater compositional diversity than MOF-74, few have shown the sufficient compositional variety and potential to overtake MOF-74 type, in terms of the CO_2_ capture capacity. One example is a large family of materials with the MIL-88 type structure, also denoted as the *acs* type^28^,^29^. It is based on 6-connected trimeric clusters crosslinked by a generic and often linear dicarboxylate. Even though it has far greater compositional diversity than MOF-74 in terms of both metal and ligand types and is also equipped with open metal sites (one per metal site, same as in MOF-74), the CO_2_ uptake properties of known MIL-88 materials are dismal compared with MOF-74 series.

Here, we report a multi-step and dramatic development of a powerful platform that has led to a family of CPMs (nomenclature for all new materials in Supplementary Table 1) with exceptional CO_2_ uptake properties (Table 1). This platform, based on the structure type denoted here as the *pacs* type (*pacs*=partitioned *acs*), is far more tunable than the MOF-74 type in both metal and ligand types and offers an outstanding alternative to the MOF-74 platform. The *pacs* family of materials is fundamentally different from the MOF-74 family, because the former does not have any open metal sites and exhibits a totally different mechanism for high CO_2_ uptake. It is because of its unique mechanism, the *pacs* family of materials could accomplish extraordinarily high levels of CO_2_ uptake capacity comparable to MOF-74 (actually higher than MOF-74 at ≤273 K and 1 atm), yet with a very low heat of adsorption (*Q*_st_) that is only about half of that for MOF-74 and is among the lowest for MOFs. Such exceptional combination of low-*Q*_st_ and high-capacity uptake could bring a distinct economic advantage because of the significantly reduced energy consumption for low-temperature activation (≈60 °C) and regeneration of adsorbents. Also due to the low *Q*_st_, the process of activation, adsorption, and regeneration induces no strain on the host frameworks, and these *pacs* materials suffer no loss in crystallinity through these processes (in fact, better crystallinity is often seen after gas sorption measurements), which indicates excellent recyclability of these extraordinary adsorbents.

## Results

### Advancing Mg-MOFs beyond the chain-type MOF-74-Mg

**Figure 1:**
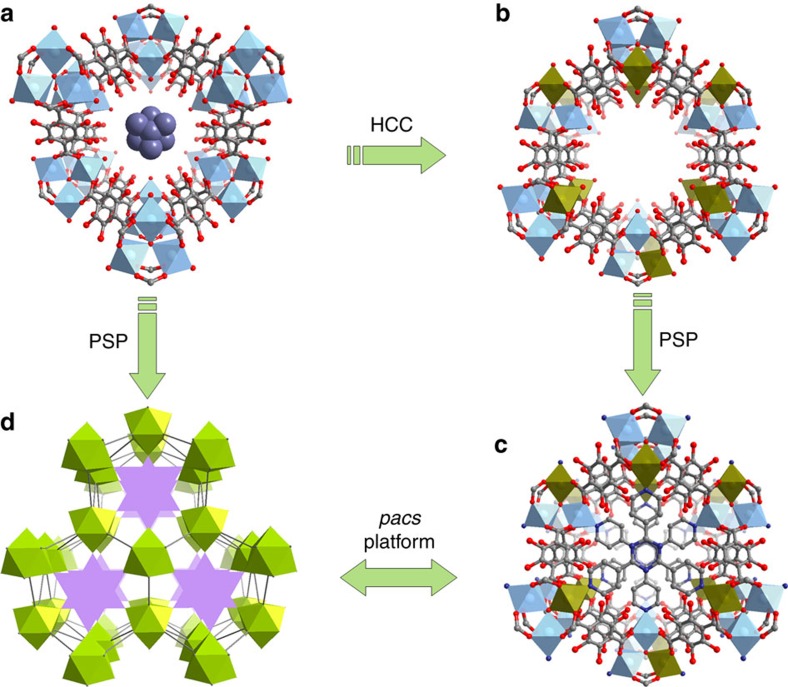
**Stepwise design of the** ***pacs*****-MOF platform.** (**a**) Anionic framework of **Mg3-MIL-88** with the charge-balancing [(CH_3_)_2_NH_2_] cations simplified as blue balls. (**b**) V^3+^-containing neutral framework of **Mg2V-MIL-88**. (**c**) Pore space partition through TPT insertion in **Mg2V-DHBDC**. (**d**) Polyhedral drawing of PSP through TPT insertion in **Mg3-DHBDC**. TPT=2,4,6-tri(4-pyridinyl)-1,3,5-triazine.

Our first-step strategy in this study is inspired by the chemical composition and the recording-setting CO_2_ uptake property of MOF-74-Mg. A systematic study of Mg-MOFs culminates with the eventual creation of **CPM-140** (also denoted here as **Mg3-MIL-88,** the *acs* type) built from [Mg_3_(OH)(RCOO)_6_]^−^ clusters. Crystals of **CPM-140** were synthesized by a solvothermal reaction of Mg(NO_3_)_2_·6H_2_O and H_2_DHBDC at 100 °C for 5 days and crystallize in *P*6_3_/*mmc* symmetry (Supplementary Methods, Supplementary Table 9). [Mg_3_(OH)(COO)_6_] trimeric clusters are joined by DHBDC^2−^ to form a MIL-88-type framework (Fig. 1a; Supplementary Fig. 13). It is worth noting that although the [M_3_(μ_3_-OH/O)(COO)_6_] cluster is common for trivalent V, Cr, Fe, and In or mixed di- and tri-valent Fe, Co and Ni^30^, this pure M^2+^-based [Mg_3_(μ_3_-OH)(COO)_6_] trimer is quite unusual. **CPM-140** is the first example of MIL-88 type with anionic framework.

**Figure 2:**
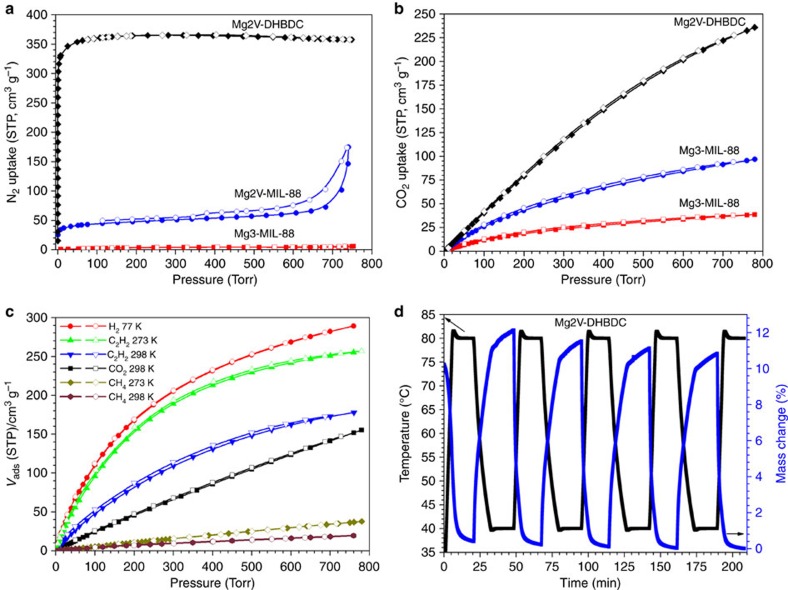
Gas sorption study on MIL-88 and pacs-MOF compounds. (**a**) N_2_ sorption isotherm at 77 K. (**b**) CO_2_ sorption at 273 K. (**c**) H_2_ (77 K), CO_2_ (298 K), CH_4_ (273 and 298 K), and C_2_H_2_ (273 and 298 K) adsorption isotherms. (**d**) Repeated adsorption–desorption kinetics between a 15: 85 CO_2_/N_2_ (v/v) flow at 313 K and a pure N_2_ flow at 353 K.

**CPM-140** exhibits negligible N_2_ adsorption (Supplementary Fig. 17), but has a significant CO_2_ uptake of 35.6 cm^3^ g^−1^ (1.59 mmol g^−1^) at 273 K and 1 atm (Fig. 2a). This performance of **CPM-140,** in terms of CO_2_ uptake, is about 15% of that of the best performing MOF-74-Mg. Despite such unimpressive CO_2_ uptake, the successful synthesis of **CPM-140** enabled the subsequent two design strategies that culminate in the creation of **CPM-231** with the record-setting CO_2_ uptake at≤273 K.

### Heterometallic cooperative crystallization and charge tenability

Our success in transitioning from the chain-type Mg-MOFs to cluster-based Mg-MOFs broadens the structure types accessible by Mg-MOFs, however, throughout our study of cluster-based Mg-MOFs, we were plagued by the repeated observation of lower stability and lower porosity of our newly created cluster-based Mg-MOFs. To address this issue, our second-step strategy focuses on the incorporation of higher-valent metal ions^31^ into **Mg3-MIL-88**. (We later also discovered that our third-step strategy could independently solve the above issue. See Table 1 for high-performance Mg_3_-cluster based materials made in this work.)

The addition of VCl_3_ to the reaction condition of **CPM-140** results in **CPM-230** built from heterometallic [Mg_2_V(μ_3_-OH)(COO)_6_] clusters. The use of VCl_3_ in the absence Mg^2+^ led to clear green solution, demonstrating that the formation of **CPM-230** is a cooperative crystallization process between Mg^2+^ and V^3+^. The Mg/V ratio of 2, determined from single crystal structure refinement and the EDS analysis (Supplementary Fig. 12), leads to a charge-neutral MIL-88-type framework (Fig. 1b). This heterometallic cooperative crystallization (HCC) strategy boosts the CO_2_ uptake capacity by 250% from 35.6 cm^3^ g^−1^ in CPM-140 to 95.3 cm^3^ g^−1^ in CPM-230 at 273 K and 1 bar. Still the value for CPM-230 is only 42% of that of MOF-74-Mg, and additional molecular engineering is required to reach or surpass the level of MOF-74-Mg.

### Pore space partition through Mg-N coordination bond

Here Pearson matching refers to the well-known principle that hard acids prefer to bond with hard bases and soft acids prefer to bond with soft bases. Our third-step strategy originates from our recently introduced concept called pore-space partition or PSP^32^. This concept and its various forms of implementation can simultaneously improve multiple key factors affecting gas sorption properties such as density of guest binding sites and framework stability. This third-step strategy involving the insertion of a PSP ligand (for example, **TPT**, 2,4,6-tri(4-pyridinyl)-1,3,5-triazine) to partition the pore space of Mg-based MOFs was initially thought to be highly unlikely based on hard-soft acid-base principle as observed in known MOFs.

Before this work, we knew that TPT could partition the pore space of Ni-based structures through Ni-N bond formation^33^. Following our step-one and step-two synthesis of Mg_3_ and Mg_2_V-based **CPM-140** and **CPM-230**, it is foreseeable that the pore partition of these phases could boost CO_2_ uptake properties. Given the hardness of the Mg^2+^ sites (Pearson hardness parameter η for Mg^2+^ is 32.55, which is nearly four times that for Ni^2+^, 8.50 (ref. 34)), our initial strategy was to use ligands with oxygen donor atoms as the PSP ligand to bond to Mg^2+^/V^3+^ sites. After unsuccessful tries with O-donor ligands, we came to the realization that the symmetry matching between the PSP ligand and the *acs* framework is perhaps more crucial than the Pearson matching (for PSP to be successful, there is also an issue of size matching between dimensions of channels and size of pore partitioning ligands, but the size matching can be more easily satisfied due to the flexibility of MIL-88 net and the availability of different dicarboxylates to tune the channel dimension). As a result of this new realization, the pore space partition was achieved by the addition of 2,4,6-tri(4-pyridyl)-1,3,5-triazine (TPT) in the reaction conditions of **CPM-140** and **CPM-230** to generate **CPM-141** and **CPM-231**. Clearly, pore space partition has the added power of being able to stabilize bonds (for example, Mg-N) that are otherwise much less likely to form. This phenomenon is related to the observation that the host-guest chemistry can be used to stabilize reactive intermediates.

### Stepwise framework change and dramatic gas sorption tuning

In contrast to the *acs*-type topology of **CPM-140** and **CPM-230**, in which the trimers are 6-connected, [Mg_2_V(μ_3_-OH)(COO)_6_] trimers in **CPM-231** (**Mg2V-DHBDC**) are 9-connected with three axial positions occupied by pyridyl groups from inserted TPT ligands (Fig. 1c). The occupation of open metal sites by TPT ligands increases the framework connectivity, which prevents the breathing effect of MIL-88 structure. Such rigidification leads to a robust structure of **CPM-231**. This was supported by the thermal stability results (Supplementary Fig. 11) and the water humidity stability experiments. Neither **Mg3-MIL-88** nor **Mg2V-MIL-88** is stable under 75% humidity, but the framework of **Mg2V-DHBDC** is stable for at least 15 days under the same condition (Supplementary Fig. 6), despite the presence of Mg-N and V-N bonds. In addition to greater stability, the insertion of TPT in **CPM-231** divides the original continuous hexagonal channel along the *c*-axis into infinite number of small finite segments (Fig. 1d). For example, by partitioning with TPT ligand, the infinite channel in **CPM-230** was fragmented into numerous small segments of about 4.5 Å in length for **CPM-231** with potential for enhanced confinement effects. Moreover, the height of each segment of cylindrical pore can also be varied by adjusting the length of the dicarboxylate ligand^33^. For **CPM-237** with 26NDC, the distance between two adjacent TPT is about 7.0 Å. Along with this variation in the length of the *c* axis is the increased density of the effective host surface exposed to the adsorbate. Overall, the presence of PSP, lightweight Mg^2+^, robust water stable frameworks, and greater density of binding sites is believed to be key factors contributing to its exceptional gas adsorption properties.

As we transition step-by-step from pure magnesium **CPM-140** to heterometallic **CPM-230** and further to pore-space-partitioned **CPM-231**, a dramatic increase in the CO_2_ uptake capacity has been accomplished even with the loss of all open metal sites and reduced binding affinity. Significantly, the CO_2_ uptake of **CPM-231** at 273 K and 1 bar reaches 232.3 cm^3^ g^−1^ (10.37 mmol g^−1^, 45.6 wt %). This value compares favourably with that of MOF-74-Mg (<230 cm^3^ g^−1^ under the same conditions) which has been the highest value since its discovery. Impressively, such an extraordinary high uptake capacity by **CPM-231** is accomplished with an isosteric heat of adsorption (*Q*_st_) as low as 20.4 kJ mol^−1^, less than half of that of MOF-74 (ref. 26). The low-*Q*_st_ and high-capacity features possessed by **CPM-231** could enable new or more cost-effective applications in gas storage and separation.

### The establishment of an ultra-tunable platform

The *pacs* type exhibited by **CPM-231** is a highly tunable platform for property engineering. Countless new MOFs can be made on this platform from ligands short or long, allowing many possible applications. Since the focus of this work is CO_2_ capture under ambient conditions, only short ligands are used here. In total, we have examined 84 permutations from 7 types of dicarboxylates, 6 types of homometallic inorganic nodes, and 6 types of heterometallic nodes. So far, 45 of these permutations have been synthesized, which are summarized in Supplementary Table 1.

In addition to DHBDC, six additional dicarboxylic acid ligands with different functional groups or length (OHBDC, BDC, NH_2_BDC, NO_2_BDC, 14NDC and 26NDC) were utilized, (Supplementary Tables 9–15; Supplementary Fig. 14). In addition to Mg_3_ and Mg_2_V, other heterometallic (Mg_2_Sc, Mg_2_Ti, Mg_2_Fe, Mg_2_Ga and Mg_2_In) and homometallic (Mn_3_, Fe_3_, Co_3_, Ni_3_, and Zn_3_) compositions have also been achieved, resulting in a large family of isostructures denoted as **CPM-141** to **CPM-293** (Table 1; Supplementary Tables 1 and 2). It is worth noting that some of these heterometallic combinations (for example, Mg_2_Ti) are unprecedented in MOFs. In addition, this is also the first time that the MIL-88 type framework is accomplished with new types of clusters such as [Mn_3_(μ_3_-OH)(COO)_6_] and [Zn_3_(μ_3_-OH)(COO)_6_]. Some of these trimer compositions are quite rare in MOFs, likely because they are difficult to form. Yet on the *pacs* platform, these can all be readily crystallized.

A powerful platform goes beyond the simple ability to accommodate a large variation in chemical, structural and geometric factors such as different chemical compositions, attachment of substituent groups, lengthening or shortening of ligands or coordination bonds. The *pacs* platform is superbly adaptable in all these aspects. What is the most impressive about the *pacs* platform is, however, its power to induce the formation of chemical entities that are otherwise unable to form or exist. It appears that the PSP ligand does not just serve as the secondary ligand to partition the channels of pre-formed primary MOF frameworks. Rather, it appears that the PSP ligand plays an active role in templating and assembling the primary MOF framework, which is why chemical species (for example, Mg_2_Ti) not seen elsewhere in MOFs are also found in the *pacs* platform. To our knowledge, the *pacs* platform is arguably the most versatile one among crystalline porous materials.

### High-performance gas sorption of the *pacs* platform

New materials with very high surface areas can be easily made on the *pacs* platform by using elongated dicarboxylates, as well as lengthened PSP ligands. However, this study is primarily focused on gas capture properties under ambient conditions. Another focus is on the effect of various homometallic and heterometallic compositions. For this reason, ligands selected in this study are mostly based on short BDC derivatives to maximize uptake capacity for CO_2_, H_2_, C_2_H_2_ and CH_4_ under ambient conditions.

The N_2_ sorption isotherms at 77 K indicate a typical type I sorption behaviour for all *pacs* materials reported here (Fig. 2a; Supplementary Figs 18–50). Compared with the negligible or small N_2_ uptakes of **CPM-140 (Mg3-MIL-88)** and **CPM-230 (Mg2V-MIL-88)**, **CPM-231 (Mg2V-DHBDC)** exhibits a saturated sorption amount of 366.5 cm^3^ g^−1^ (16.4 mmol g^−1^, Fig. 3b). The Langmuir and BET surface areas of **CPM-231-237**, tuned by the functional groups and the length of the dicarboxylate linkers, vary from 823 to 2,117 and 588 to 1,475 m^2^ g^−1^ (Supplementary Tables 3–7). For CPMs with the same dicarboxylate linker, the BET surface areas are also dramatically tuned by the metal types (Supplementary Fig. 16). Among all pacs CPMs reported here, the Mg_2_Sc MOFs have the highest values, while Mg_3_ MOFs show the smallest BET surface areas due to the existence of charge-balancing cations.

The sorption properties of several fuel molecules such as H_2_, C_2_H_2_ and CH_4_ have also been studied. At 77 K and under 1 bar, **CPM-231** can adsorb 289.0 cm^3^ g^−1^ (2.58 wt%) H_2_ (Fig. 2c). Under the same condition, the H_2_ uptake values for all other iso-MOFs range from 136.6 cm^3^ g^−1^ (1.22 wt%) to (326.1 cm^3^ g^−1^, 2.91 wt%) (Supplementary Tables 3-7). The uptake capacity of **CPM-231** for C_2_H_2_ and CH_4_ reaches 255.1 and 36.7 cm^3^ g^−1^, respectively at 273 K and 1 bar. Its corresponding value at 298 K is 177.6 and 24.0 cm^3^ g^−1^ for C_2_H_2_ and CH_4_ (Fig. 2c). Notably, the C_2_H_2_ uptake value (255.1 cm^3^ g^−1^ at 273 K and 1 bar) is the highest one among all reported MOFs without open metal sites, which is only less than the record of 290 cm^3^ g^−1^ for ZJU-5 (ref. 35) and 277 cm^3^ g^−1^ for FJI-H8 (ref. 36) with open Cu sites under the same conditions. Also, other Mg2V MOFs (**CPM-232-237**) all show superior sorption performance for C_2_H_2_ at 273 K (Supplementary Tables 3–7).

Under 1 bar, the CO_2_ uptake of **Mg2V-DHBDC** (**CPM-231**) at 273 K reaches 232.3 cm^3^ g^−1^ (10.37 mmol g^−1^, 45.6 wt%, Fig. 2b), which outperforms all known MOFs including Mg-MOF-74 (10.22 mmol g^−1^)^9^ under the same conditions. At 298 K and 1 bar, **CPM-231** can absorb CO_2_ of 151.6 cm^3^ g^−1^ (6.77 mmol g^−1^, 29.8 wt%, Supplementary Fig. 35). This value is also impressive, but is only lower than the values of Mg-MOF-74 (8.06 mmol g^−1^)^26^, Cu-TDPAT (7.94 mmol g^−1^)^6^, Co-MOF-74 (6.96 mmol g^−1^)^26^ and MAF-X25ox (7.14 mmol g^−1^)^37^ (Supplementary Table 8). The highest CO_2_ uptake at 273 K or below and the fifth highest uptake at 298 K is related to the unique framework compositions and the pore-space partition features of our *pacs* platform.

### Advantages of PSP over open metal sites for gas sorption

Going from **CPM-230** to **CPM-231**, three open metal sites in **CPM-230** are taken by three pyridyl groups of pore-partitioning ligand TPT. A comparison of CO_2_ capture uptake properties of **CPM-230** and **CPM-231** shows the PSP is superior to open metal sites in enhancing CO_2_ uptake. This was supported by the fact that **CPM-230** (**Mg2V-MIL-88**) without TPT insertion just has CO_2_ uptakes of 95.3 cm^3^ g^−1^ (4.25 mmol g^−1^, 273 K) and 55.1 cm^3^ g^−1^ (2.46 mmol g^−1^, 298 K, Supplementary Fig. 34) at 1 bar. Due to the presence of open metal sites in **CPM-230**, the isosteric heat plot for CO_2_ sorption is markedly higher than that of **CPM-231** (Supplementary Fig. 55). Also, following calculation clearly shows that PSP contributes much more to the extraordinarily high CO_2_ storage capacity of **CPM-231** compared with three open metal sites. For **CPM-230**, the open metal site density is 3.96 mmol g^−1^. The amount of CO_2_ gas adsorbed at 273 K and 1 bar for **CPM-230** is 95.3 cm^3^ g^−1^ (4.25 mmol g^−1^), which corresponds to 1.07 CO_2_ molecules per open metal sites. Assuming that each open metal site can bind one CO_2_ molecule, the open metal sites contribute to 88.7 cm^3^ g^−1^ of total 95.3 cm^3^ g^−1^ CO_2_ storage capacity at 273 K and 1 atm, which means that the total pore space channel within MIL-88 framework only attribute 6.6 cm^3^ g^−1^ of the CO_2_ uptake. After partitioned by TPT and losing all open metal sites from **Mg2V-MIL-88** (**CPM-230**), the metal and TPT densities of **CPM-231** are of 2.94 mmol g^−1^ and 0.98 mmol g^−1^, respectively. The amount of CO_2_ gas adsorbed at 273 K and 1 bar for **CPM-231** reaches to 232.3 cm^3^ g^−1^ (10.37 mmol g^−1^), which corresponds to 3.52 CO_2_ molecules per metal and 10.58 CO_2_ molecules per TPT. Clearly, the implementation of PSP with loss of open metal sites in MIL-88 frameworks is a great success to improve the CO_2_ storage capacity. Also, taking advantage of PSP, **CPM-141** (**Mg3-DHBDC**) shows a dramatically high CO_2_ uptake performance (Table 1, 194.6 cm^3^ g^−1^, 8.69 mmol g^−1^, 273 K and 1 bar; 123.8 cm^3^ g^−1^, 5.53 mmol g^−1^, 298 K and 1 bar) even with part of the pore space occupied by [(CH_3_)_2_NH_2_]^+^ cations.

### Effects of ligand and metal types on CO_2_ capture properties

The CO_2_ uptake properties of most CPMs reported here (for example, **CPM-231**, **CPM-232**, **CPM-233**,...) are outstanding compared with almost all other MOFs (Supplementary Table 8). Still it is clear that CO_2_ uptake properties can be tuned by the types of ligands. Especially, we observed that the DHBDC^2−^ ligand, or more precisely, the hydroxyl group, contribute to the CO_2_ uptake properties. This is evident by comparing **CPM-231** with (OH)_2_-BDC with two isoreticular phases with only one –OH (**CPM-232** with OH-BDC) and no –OH (**CPM-233** with BDC) group. At 273 K, the CO_2_ uptakes under 1 bar are 210.6 cm^3^ g^−1^ (9.40 mmol g^−1^, 37.6 wt%, Supplementary Fig. 36) for **CPM-232** and 171.4 cm^3^ g^−1^ (7.65 mmol g^−1^, 33.7 wt%, Supplementary Fig. 37) for **CPM-233**, showing a gradual decreases of CO_2_ uptake with the loss of –OH groups. The beneficial effect of the -OH group is further shown by lower CO_2_ uptake properties of **CPM-234** (with -NH_2_) and **CPM-235** (with -NO_2_) (Supplementary Figs 38–39). It is also established that the short length of the BDC core contributes significant to the CO_2_ uptake, because **CPM-236** (with 1,4-NDC, Supplementary Fig. 40) and **CPM-237** (with 2,6-NDC, Supplementary Fig. 41) show much lower CO_2_ adsorption capacity (Supplementary Fig. 15).

Compared with MOFs with open metal sites such as MOF-74 series, the effect of metal types on gas sorption in the *pacs* family of materials is subtle because the *pacs* materials have no open metal sites and do not involve direct metal–gas interactions. As shown in Table 1 and Supplementary Fig. 15, for a given dicarboxylate linker, Mg2M (M=Sc^3+^, Ti^4+^, V^3+^, Fe^3+^, Ga^3+^ and In^3+^) heterometallic CPMs exhibit exceptionally high CO_2_ adsorption ability at 273 K and 1 bar. Among six series of homometallic CPMs (Mg_3_, Mn_3_, Fe_3_, Co_3_, Ni_3_ and Zn_3_), Mn_3_ and Zn_3_ materials have significantly lower CO_2_ uptakes for the same dicarboxylate linker. Still, compared with most other MOF materials, nearly all *pacs* MOFs have outstanding gas sorption properties (Supplementary Table 8).

### CO_2_ adsorption dynamics by inelastic neutron scattering

To further understand the high CO_2_ uptake performance of these *pacs* MOF, inelastic neutron scattering (INS) were used to visualize the binding dynamics for adsorbed CO_2_ molecules in **CPM-231**. INS is a powerful neutron spectroscopy technique that has been used widely to investigate the H_2_ binding interactions within various storage systems by exploiting the high neutron scattering cross-section of hydrogen. However, this technique cannot directly detect the CO_2_ binding interaction within a carbon capture system because the scattering cross-sections for C and O are too small to obtain a clear neutron scattering signal^10^. In this study, we successfully combined INS and DFT to visualize captured CO_2_ molecules within **Mg2V-DHBDC** (**CPM-231**) by investigating the change in the dynamics of the hydrogen atoms of the local MOF structure, including those of the hydroxyl groups and benzene or pyridine rings of the ligand. As can be seen from the Supplementary Fig. 58, the simulation qualitatively reproduced the main features in the INS spectrum, including the low energy band below 30 meV, the two sharp peaks near 50 meV, as well as the group of peaks ranging from 80 meV to 200 meV. The vibrational modes responsible for the peaks can then be assigned. Specifically, the low energy peaks are primarily due to the flapping of the 2,5-dihydroxybenzene and 4-pyridyl rings associated with the torsion of the axial C–C bonds; the sharp peaks near 50 meV correspond to the torsion modes of the C–O bonds. The series of peaks in 80–200 meV can be attributed to the out-of-plane and in-plane bending modes of the C–H and O–H bonds. One important observation in the INS experiment is that, upon dosing of CO_2_, there is an obvious blue shift of the low energy modes. With the above peak assignment, this observation can be easily understood: the CO_2_ molecules will most likely go to the empty space between the layers/rings in the MOF, and this should mostly affect the flapping modes of the rings. As the empty space is filled, these modes should be hindered and stiffened, causing the blue shift. In contrast, other modes at higher energy are more local and do not involve displacement of the entire ring, and they are thus less affected by the CO_2_ dosing.

### Potential applications in CO_2_ capture and CO_2_/CH_4_ separation

**CPM-231**, **-232** and **-233** were evaluated as potential adsorbents for scrubbing flue gas emissions by selectively adsorbing CO_2_ at a partial pressure of 0.15 bar. The CO_2_ adsorption and desorption behaviours of **CPM-231**, **-232** and **-233** under mixed-gas and dynamic conditions were analysed by thermogravimetry (Fig. 2d; Supplementary Fig. 56), in which the adsorbents were blown repeatedly using a 15: 85 CO_2_/N_2_ (v/v) mixture at 313 K (a typical flue gas environment) and a pure N_2_ flow (a typical regeneration method) at 353 K (optimized). The maximum and repeatable weight changes of **CPM-231**, **-232** and **-233** were about 11.5 wt%, 6.5 wt% and 3.5 wt%, respectively. Although such a mixed gas adsorption measurement is considerably more relevant for practical CO_2_ capture applications under flue gas conditions, it has been rarely reported in the previous studies. Notably, these quantities are comparable to other porous materials with top CO_2_ capacities, such as HKUST-1 (11.6 wt% at 293 K)^38^, mmen-CuBTTri (9.5 wt% at 298 K)^39^, and PPN-6-CH_2_DETA (11.8 wt% at 295 K)^40^.

**Figure 3:**
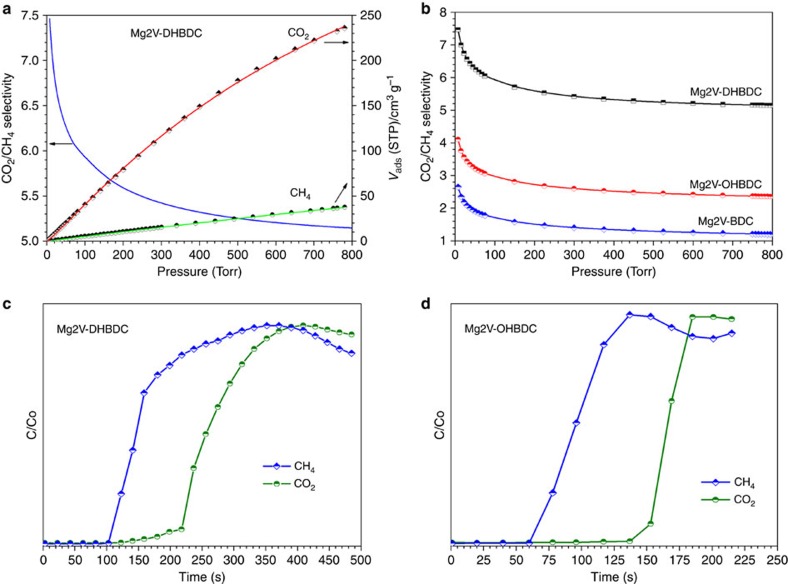
**The CO**_**2**_**/CH**_**4**_ **selectivity evaluation for Mg2V-MOFs.** (**a**,**b**) Adsorption selectivity predicted by IAST for CO_2_ (50%) over CH_4_ (50%) at 273 K. (**c**,**d**) Breakthrough curves for the separation of a 0.15:0.85 (v/v) 16 ml min^−1^ flow of CO_2_/CH_4_ mixture at 296 K.

Another possible application of these *pacs* MOFs is selective adsorption of CO_2_ over CH_4_. To predict CO_2_-CH_4_ binary mixture selectivity, an ideal adsorbed solution theory (IAST) calculation^41^,^42^,^43^,^44^,^45^,^46^ based on a Langmuir-Freundlich (LF) simulation was employed on the basis of the single-component CO_2_ and CH_4_ adsorption isotherms. Figure 3a,b shows the adsorption selectivity of **CPM-231**-**233** for CO_2_ (50%) and CH_4_ (50%) at 273 K. It shows that the CO_2_/CH_4_ selectivity values increased from 2.65 to 4.10 to 7.46 with the addition of –OH groups (Supplementary Fig. 57; Supplementary Table 3). Such tunable CO_2_ selectivity over CH_4_ could be due to the synergistic effect of functional groups and the pore space partitioning effects. The robust and tunable pacs MOFs reported here may have potential applications in separation of CO_2_ from CH_4_.

Measurements of breakthrough curves of a 0.15:0.85 (v/v) mixture of CO_2_/CH_4_ flowed through a chromatographic column packed with **CPM-231** at 296 K reveal the passage of CH_4_ through this material and the selective retention of CO_2_ (Fig. 3c). The breakthrough takes place approximately 125 s after dosing the gas mixture, which represents about 1.1 mmol of CO_2_ being retained per gram of **CPM-231** under these dynamic conditions. This kind of behaviour was expected in view of the differentiated interaction of these two gases with the **CPM-231** framework decorated with two –OH groups. When we performed a similar measurement with **CPM-232**, a related behaviour was observed with the breakthrough taking place at about 100 s (Fig. 3d) and the CO_2_ removal capacity being lowered to 0.53 mmol g^−1^ due to the absence of one –OH group.

## Discussion

We have demonstrated a powerful platform for the development of new CPMs and control of their gas sorption properties. This platform is arguably the most versatile one known to date in crystalline porous materials because it can accommodate a large variety of crosslinking carboxylate ligands, either homometallic (Mg, Mn, Fe, Co, Ni and Zn) or heterometallic metal clusters (Mg/Sc, Mg/Ti, Mg/V, Mg/Fe, Mg/Ga and Mg/In), in addition to the variation of pore-partitioning ligands. Such enormous compositional diversity allows extraordinary tunability in the pore properties and dramatic variation and enhancement of gas sorption properties. Several dozens of new CPM materials reported here are in fact only a very small number of materials that could be achieved on this platform.

Importantly, this platform, based on the structure type denoted here as the *pacs* type (*pacs*=partitioned *acs*), is fundamentally different from almost all other types of MOF platforms, because the *pacs* platform does not have any open metal sites and exhibits a totally different mechanism for high CO_2_ uptake. It is because of its unique mechanism, the *pacs* family of materials could accomplish extraordinarily high levels of CO_2_ uptake capacity even when its heat of adsorption (*Q*_st_) is among the lowest for MOFs. Such exceptional combination of low-*Q*_st_ and high-capacity uptake could bring a distinct economic advantage because of the significantly reduced energy consumption for low-temperature activation (≈60 °C) and regeneration of adsorbents. Also due to the low *Q*_st_, the process of activation, adsorption, and regeneration induces no strain on the host frameworks, and these *pacs* materials suffer no loss in crystallinity through these processes, which indicates excellent recyclability of these extraordinary adsorbents.

## Methods

### Synthesis

Experimental procedures for preparing all the crystalline porous materials achieved in this work are provided in the Supplementary Information. Here we take **Mg2V-DHBDC** (**CPM-231**) as an example to describe the preparation method. In a 20 ml glass vial, 81.2 mg of MgCl_2_˙6H_2_O, 31.5 mg of VCl_3_, 99.0 mg of 2,5-dihydroxybenzene-1,4-dicarboxylic acid (DHBDC), 62.4 mg of 2,4,6-tri(4-pyridinyl)-1,3,5-triazine (TPT) were dissolved in a mixture of 4.0 g of N,N-dimethylacetamide (DMA) and 2.0 g of 1,3-dimethyl-3,4,5,6-tetrahydro-2(1H)- pyrimidinone (DMPU). After addition of 0.3 g 85% formic acid, the vial was sealed and placed in a 130 °C oven for 5 days. The brown crystals of **CPM-231** crystallized on the walls of the glass vial after cooling to room temperature. The black solid on the bottom of the vial was discarded. Pure crystals on the walls of the vial were obtained by filtering and washing the raw product with DMA. The yield was about 20% based on Mg.

### Characterization

The single-crystal X-ray diffractions of *pacs*-MOFs, powder X-ray diffraction (PXRD), thermogravimetric analysis (TGA), energy dispersive spectroscopy (EDS), gas (N_2_, H_2_, CO_2_, CH_4_ and C_2_H_2_) adsorption-desorption isotherms at different temperatures, breakthrough curves and inelastic neutron scattering (INS) results are given in Supplementary Information. PXRD patterns of *pacs*-MOFs are given in Supplementary Figs 1-10. TGA curves of *pacs*-MOFs are given in Supplementary Fig. 11. EDS results for heterometallic MOFs and Fe3-MOFs are given in Supplementary Fig. 12. Additional crystal structures are given in Supplementary Figs 13 and 14. CO_2_ uptake performance summaries for *pacs*-MOFs are given in Supplementary Fig. 15. BET surface area and H_2_ uptake performance summaries for *pacs*-MOFs are presented in Supplementary Fig. 16. N_2_, H_2_, CO_2_, CH_4_ and C_2_H_2_ adsorption-desorption isotherms are included in Supplementary Figs 17–53. CO_2_ adsorption isostere plots and isosteric heats for CO_2_ are given in Supplementary Figs 54 and 55. Repeated adsorption–desorption kinetics for **CPM-231**-**233** are give in Supplementary Fig. 56. Adsorption selectivity data predicted by IAST are given in Supplementary Fig. 57. Comparison of the experimental and DFT-simulated INS spectra are given in Supplementary Fig. 58. Breakthrough curves are depicted in Supplementary Fig. 59. The numbering scheme and names, and synthesis conditions for *pacs*-MOFs are summarized in Supplementary Tables 1 and 2. The summary of gas sorption properties is given in Supplementary Tables 3-7. MOFs with highest CO_2_ uptake at 1 bar are summarized in Supplementary Table 8. Crystal data and structure refinements for **CPM-140-144**, **CPM-153**, **CPM-174**, **CPM-194**, **CPM-230**, **CPM-231**, **CPM-235**, **CPM-236** and **CPM-237** are given in Supplementary Tables 9-15.

### Data availability

The X-ray crystallographic coordinates for structures reported in this Article have been deposited at the Cambridge Crystallographic Data Centre (CCDC), under deposition numbers CCDC 1447148-1447157 and 1447495-1447497 (Supplementary data 1). These data can be obtained free of charge from The Cambridge Crystallographic Data Centre via www.ccdc.cam.ac.uk/data_request/cif. All remaining data are either providing in the Article and its supplementary information or are available from the authors upon request.

## Additional information

**How to cite this article:** Zhai, Q-G. *et al*. An ultra-tunable platform for molecular engineering of high-performance crystalline porous materials. *Nat. Commun.* **7,** 13645 doi: 10.1038/ncomms13645 (2016).

**Publisher's note**: Springer Nature remains neutral with regard to jurisdictional claims in published maps and institutional affiliations.

## Supplementary Material

Supplementary Information Supplementary Figures 1-59, Supplementary Tables 1-15, Supplementary Methods and Supplementary References

Supplementary Data 1 X-ray cif files.

## Figures and Tables

**Table 1:** Summary of CO_2_ uptake performance (cm^3^ g^−1^) at 273/298 K and 1 atm for *pacs*-[M_3_(OH/O)(L)_3_(TPT)] (L=dicarboxylate) MOFs reported in this work.

**M_3_ L**	**DHBDC**	**OHBDC**	**BDC**	**NH**_**2**_**BDC**	**NO**_**2**_**BDC**	**14NDC**	**26NDC**
Mg_3_	194.6/123.8	165.1/103.1	134.5/71.5	99.8/68.2	108.5/67.5	54.7/46.9	×
Mn_3_	×	123.9/81.4	107.0/-	81.6/-	—	—	—
Fe3	180.2/112.4	173.2/95.8	169.8/85.4	—	—	—	—
Co_3_	193.8/134.8	184.6/-	134.0/-	90.4/-	—	—	—
Ni_3_	187.1/127.9	172.9/108.3	134.3/74.9	—	—	—	—
Zn_3_	×	119.0/-	67.8/-	66.0/-	—	—	—
Mg_2_Sc	×	173.8/102.7	164.2/92.1	—	—	—	—
Mg_2_Ti	×	202.2/110.1	171.0/81.4	—	—	—	—
Mg_2_V	232.3/151.6	210.6/115.9	171.5/90.6	169.6/100.3	160.6/94.8	100.5/62.5	77.4/37.9
Mg_2_Fe	191.7/122.0	184.2/101.5	175.6/81.5	—	—	—	—
Mg_2_Ga	211.4/136.8	209.3/121.8	177.2/92.1	—	—	—	—
Mg_2_In	201.3/129.7	195.7/120.8	174.3/91.0	—	—	—	—

× : not yet synthesized; —: not attempted.
