# Supplementary material for: An ultra-tunable platform for molecular engineering of high-performance crystalline porous materials
Source: Nat Commun. 2016 Dec 7;7:13645. doi: 10.1038/ncomms13645 (PMC5150645; doi:10.1038/ncomms13645)
Supplement: Supplementary Information — Supplementary Figures 1-59, Supplementary Tables 1-15, Supplementary Methods and Supplementary References [file ncomms13645-s1.pdf]

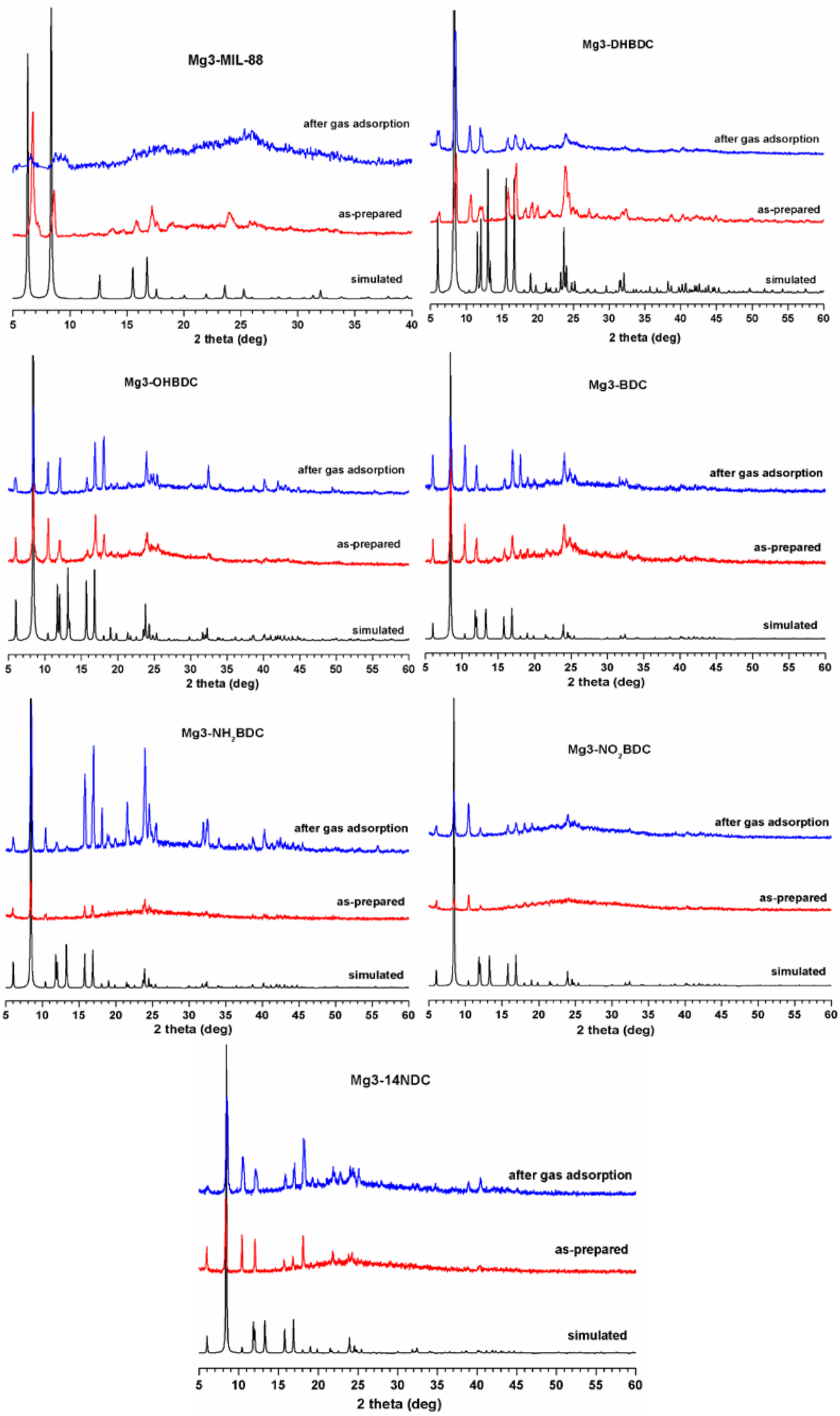

Supplementary Figure 1. Experimental and simulated PXRD patterns for **Mg3-MIL-88 (CPM-140)** and **[Mg3(OH)(dicarboxylate)$_3$(TPT)] (CPM-141-146)**.

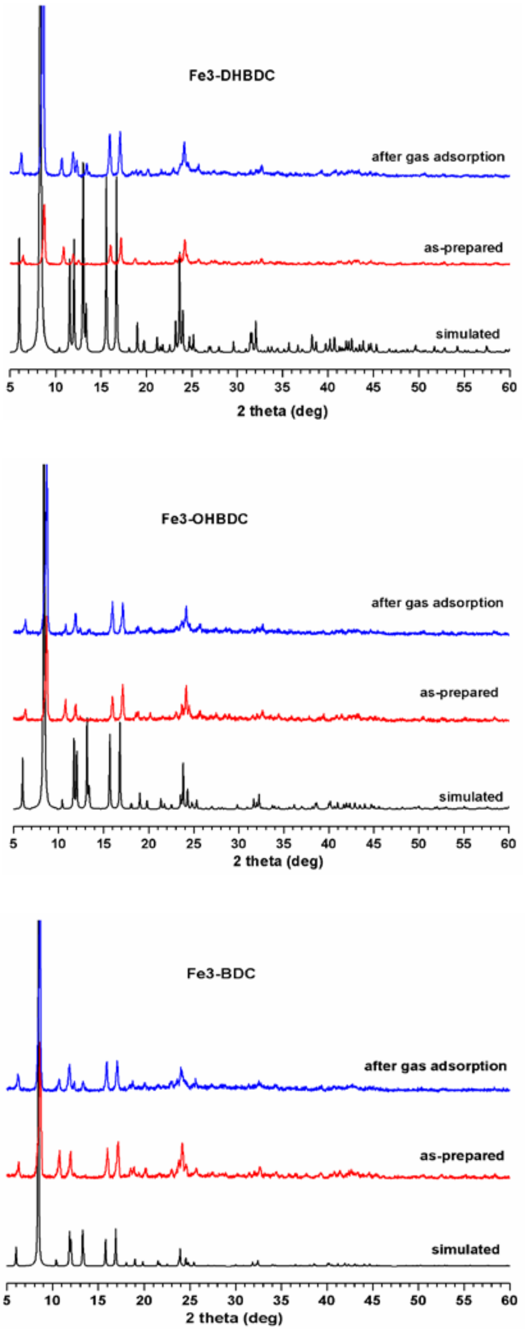

Supplementary Figure 2. Experimental and simulated PXRD patterns for **[Fe3(O)(dicarboxylate)$_3$(TPT)] (CPM-161-163)**.

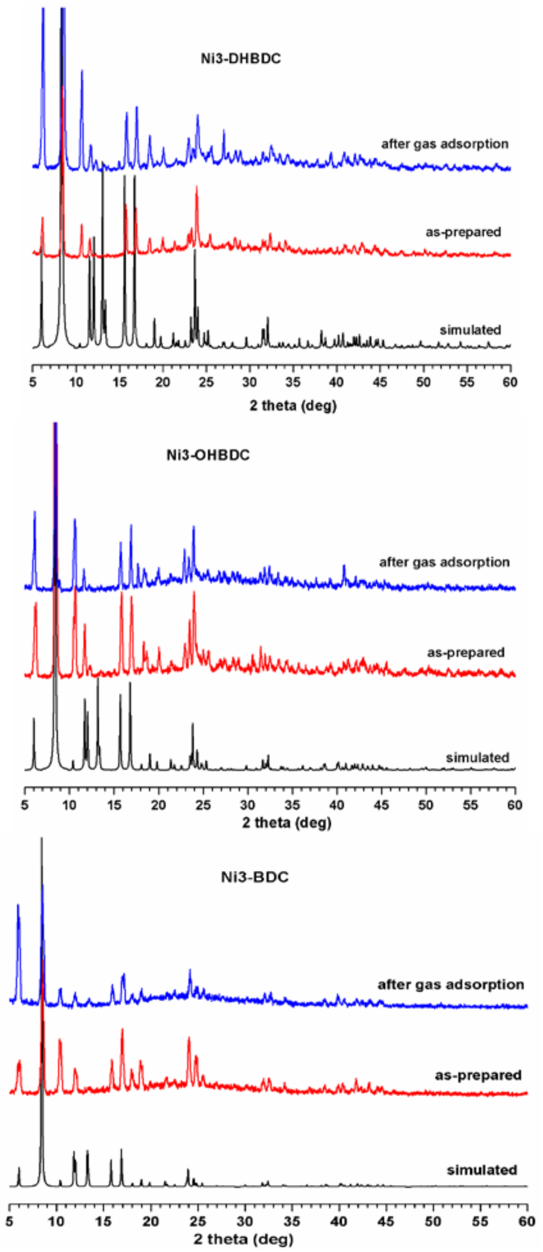

Supplementary Figure 3. Experimental and simulated PXRD patterns for **[Ni3(OH)(dicarboxylate)$_3$(TPT)] (CPM-33a, 33b, 182)**.

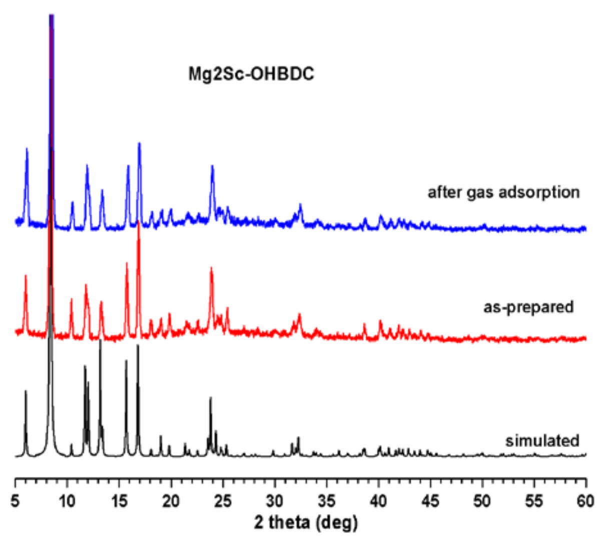

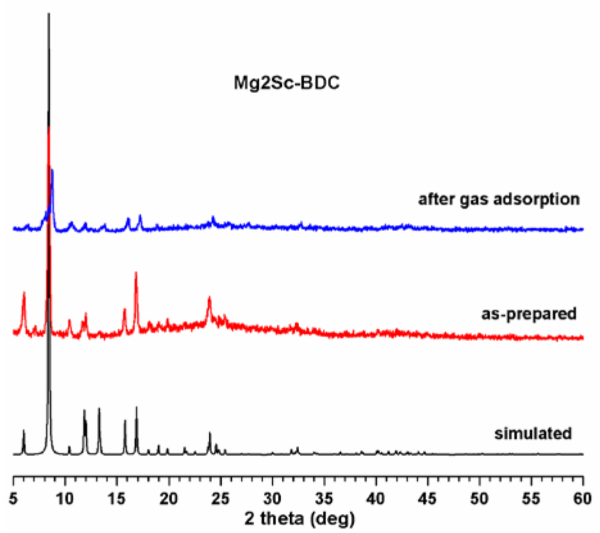

Supplementary Figure 4. Experimental and simulated PXRD patterns for **[Mg2Sc(OH)(dicarboxylate)$_3$(TPT)] (CPM-212 and 213)**.

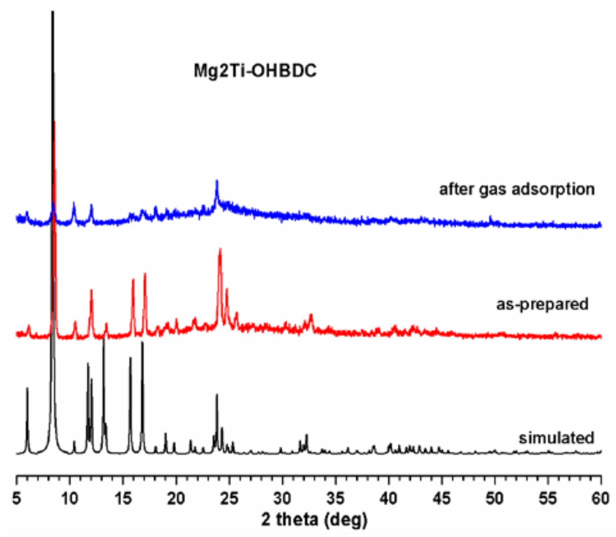

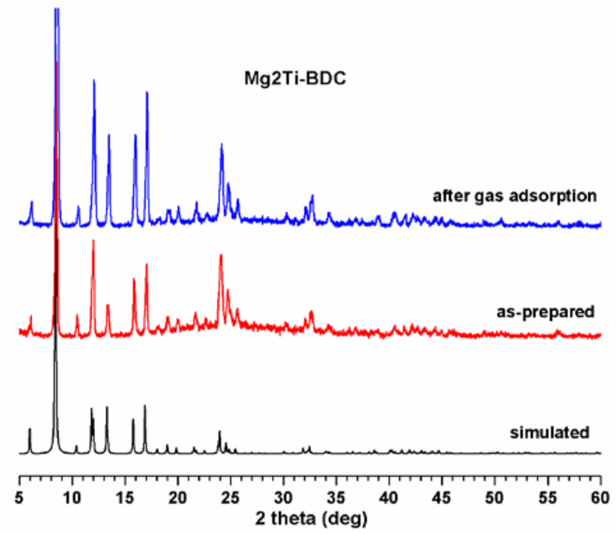

Supplementary Figure 5. Experimental and simulated PXRD patterns for **[Mg2Ti(O)(dicarboxylate)$_3$(TPT)] (CPM-222 and 223)**.

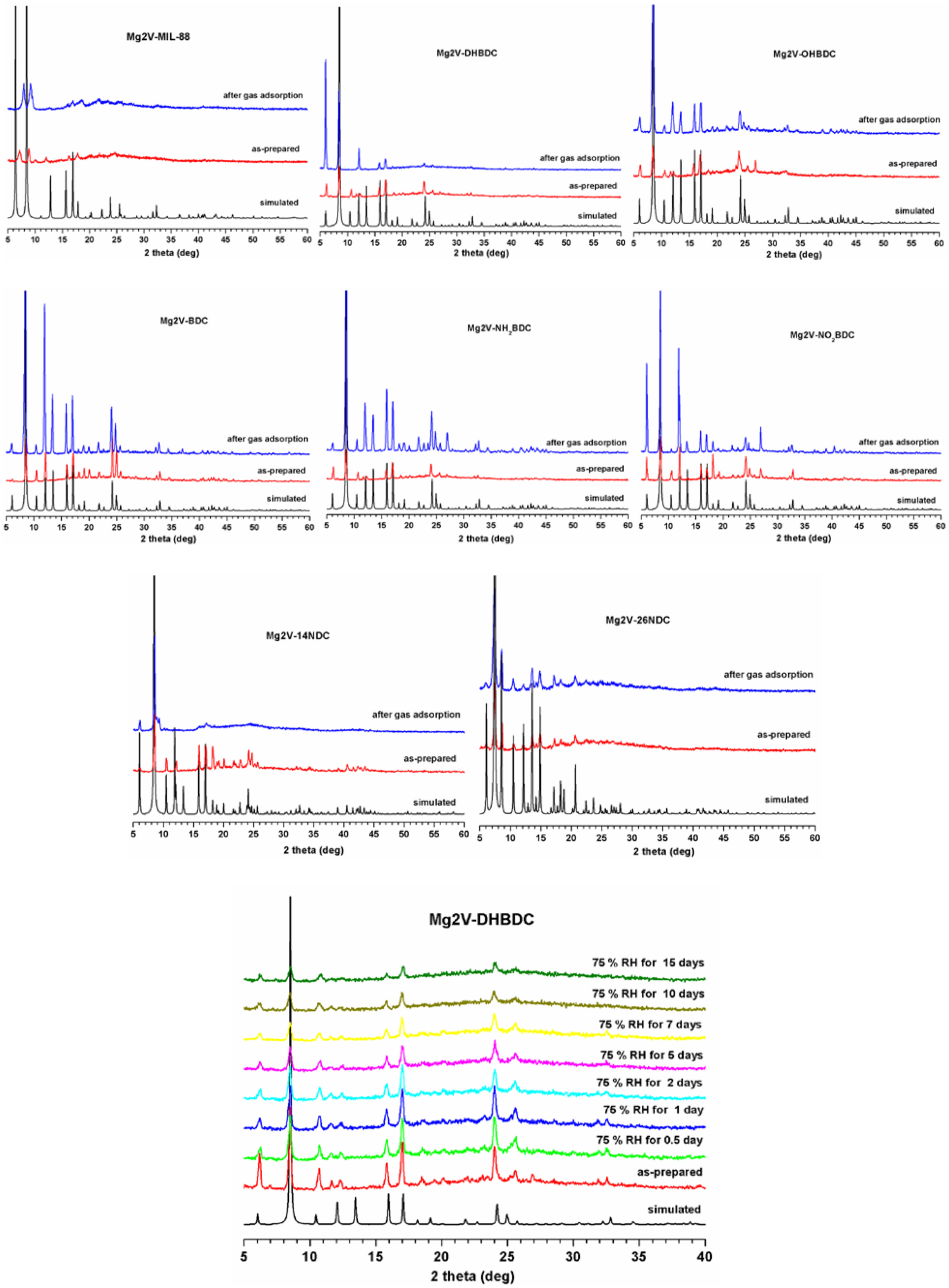

Supplementary Figure 6. Experimental and simulated PXRD patterns for **Mg2V-MIL-88 (CPM-230)** and **[Mg2V(OH)(dicarboxylate)$_3$(TPT)] (CPM-231-237)**.

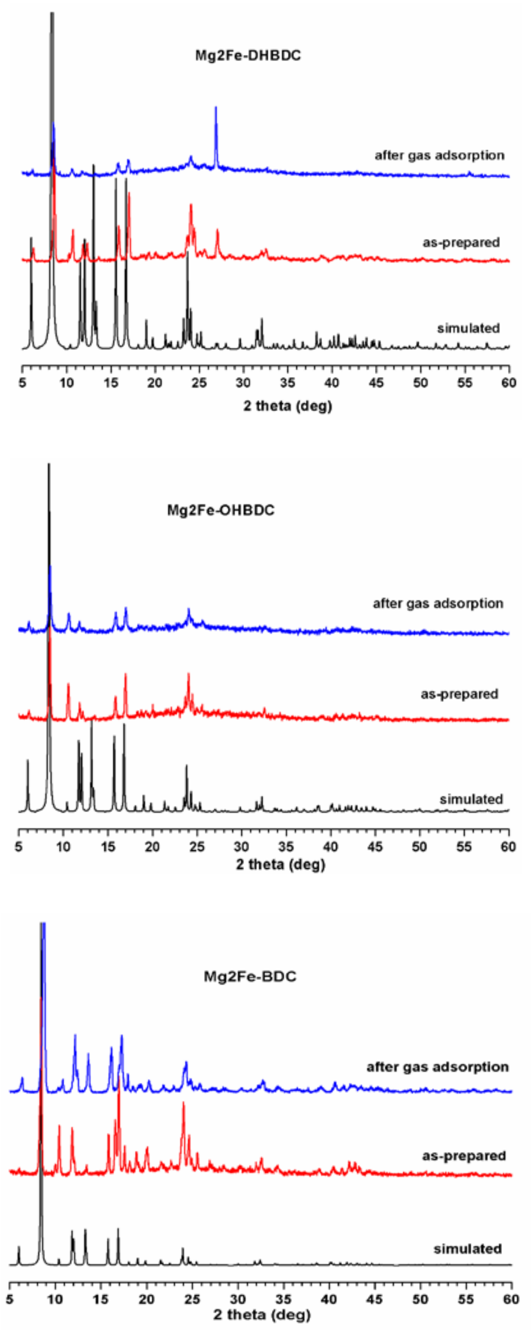

Supplementary Figure 7. Experimental and simulated PXRD patterns for **[Mg2Fe(OH)(dicarboxylate)$_3$(TPT)] (CPM-261-263)**.

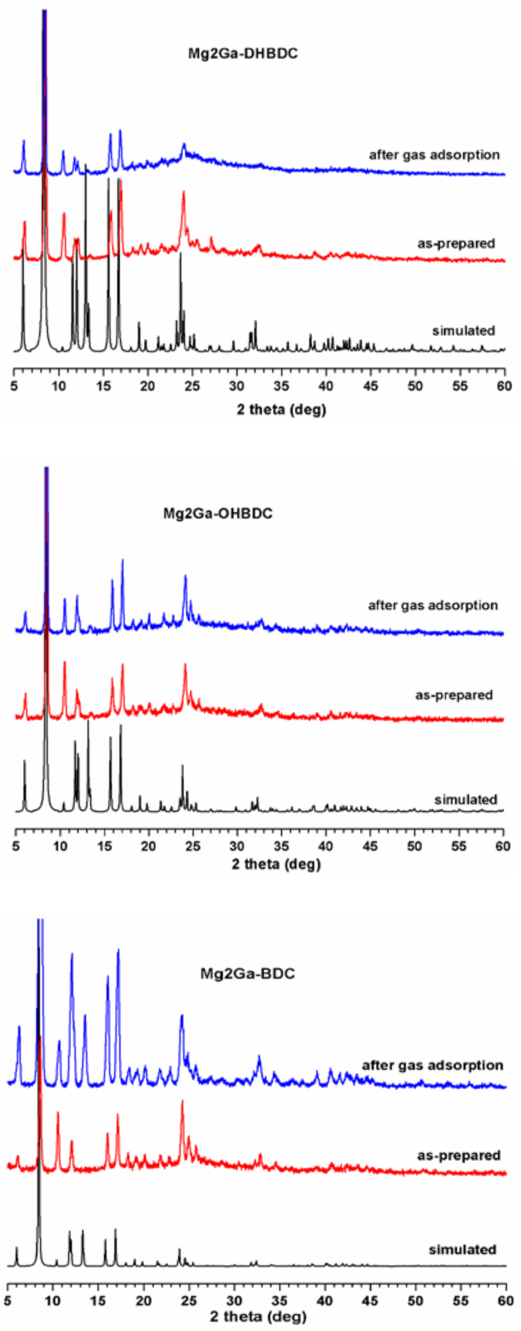

Supplementary Figure 8. Experimental and simulated PXRD patterns for **[Mg2Ga(OH)(dicarboxylate)$_3$(TPT)] (CPM-271-273)**.

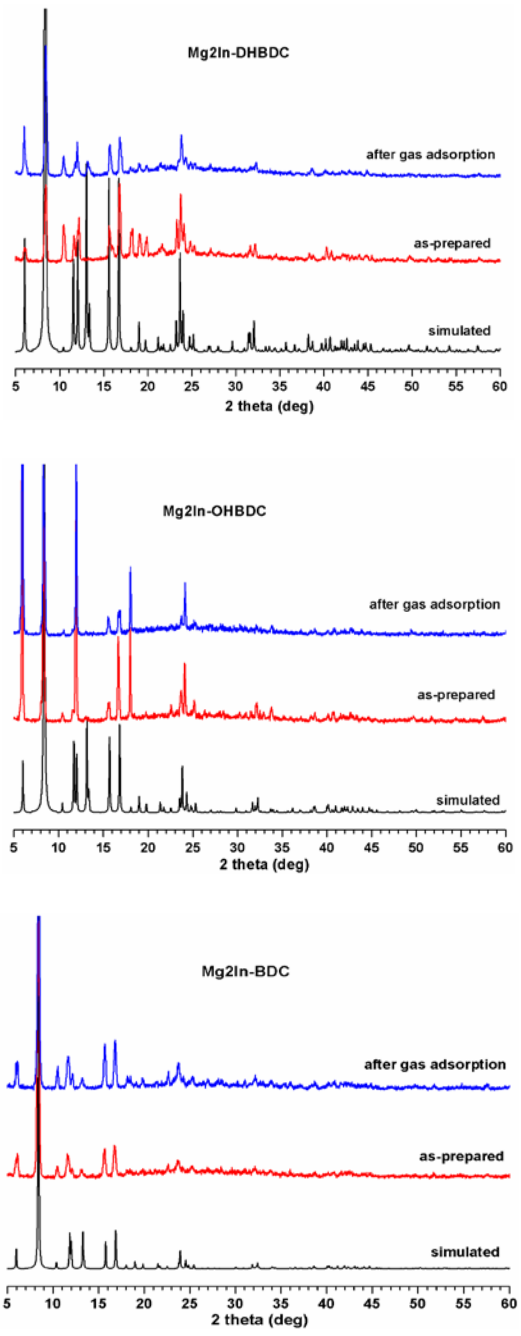

Supplementary Figure 9. Experimental and simulated PXRD patterns for **[Mg2In(OH)(dicarboxylate)$_3$(TPT)] (CPM-291-293)**.

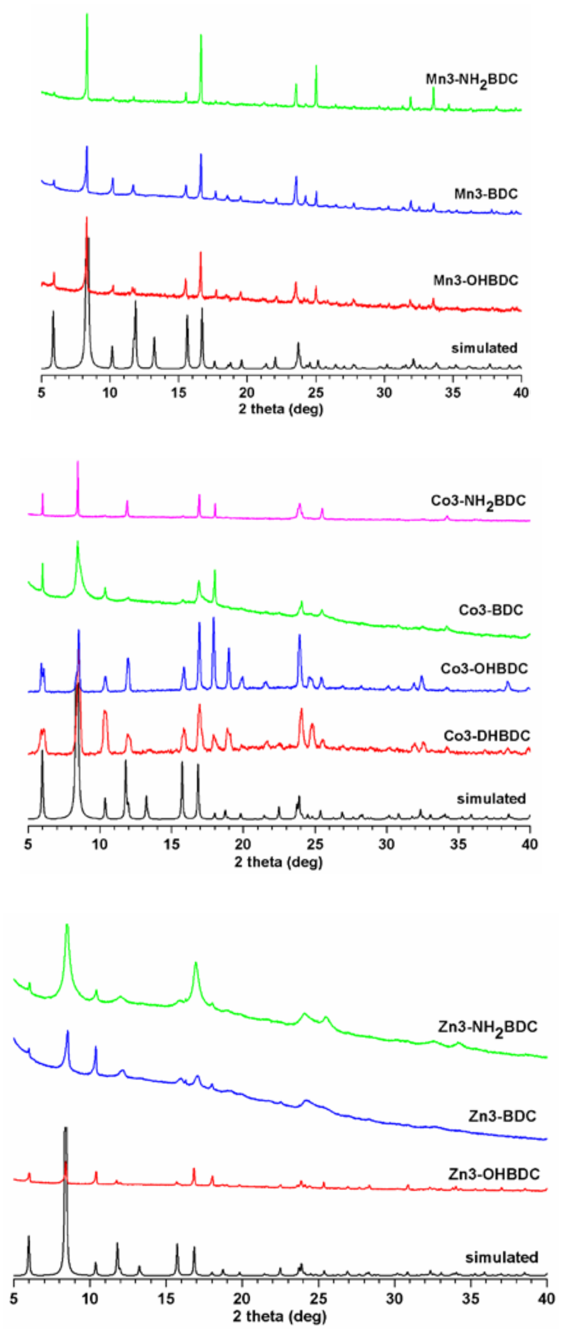

Supplementary Figure 10. Experimental and simulated PXRD patterns for **[Mn3(OH)(dicarboxylate)$_3$(TPT)] (CPM-152-154), [Co3(OH)(dicarboxylate)$_3$(TPT)] (CPM-171-174)** and **[Zn3(OH)(dicarboxylate)$_3$(TPT)] (CPM-192-194)**.

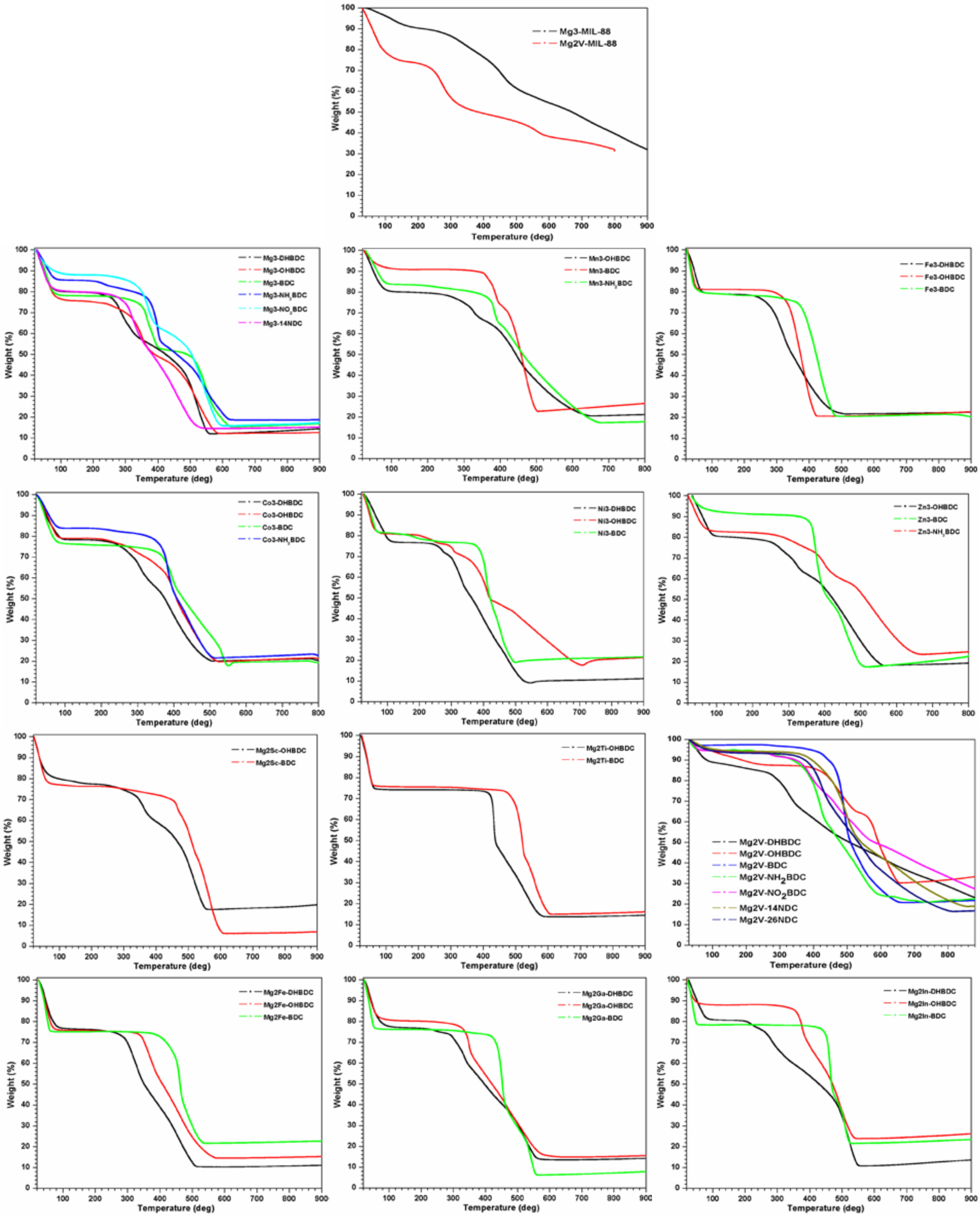

Supplementary Figure 11. TGA curves for all MOFs reported in this work.

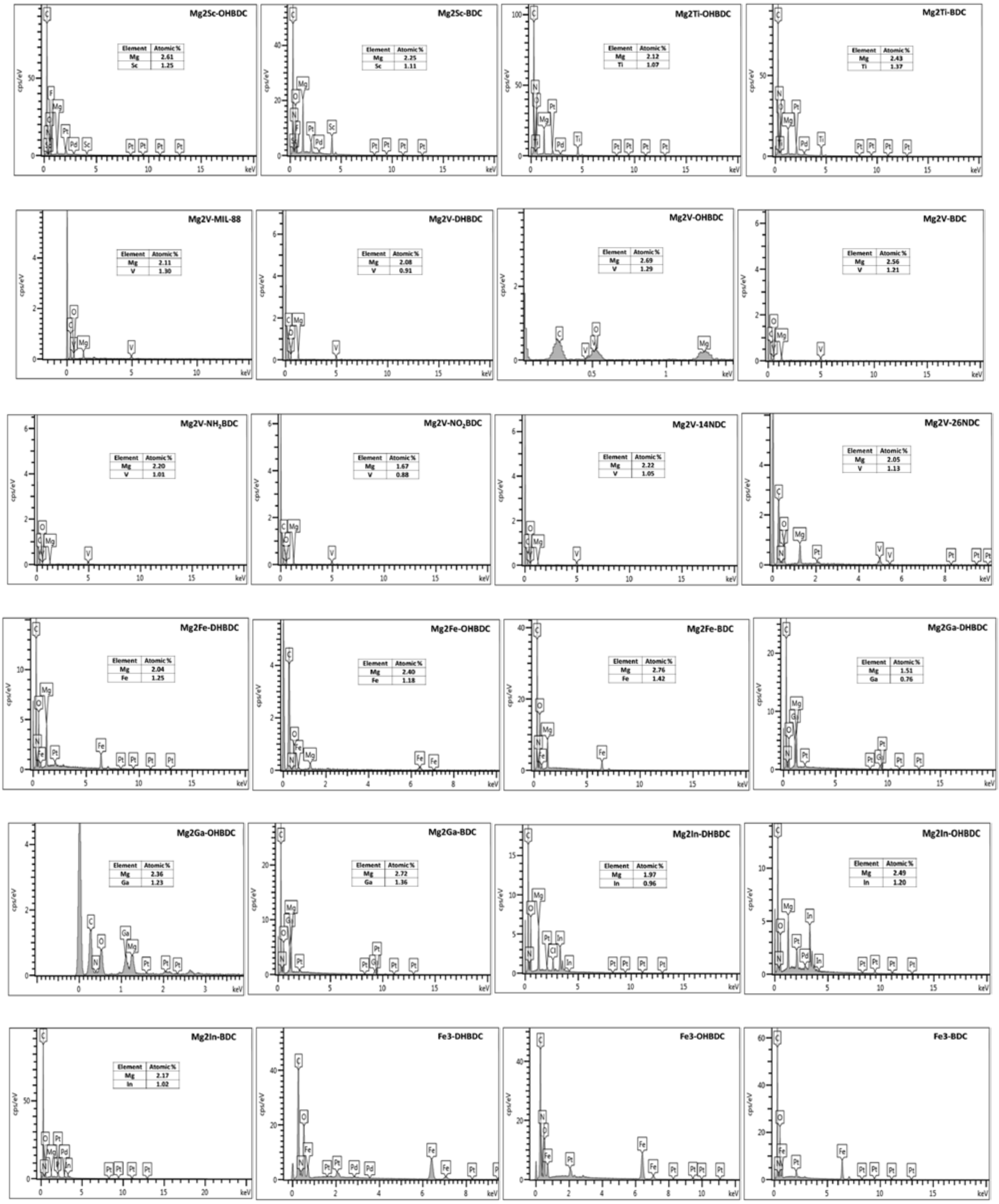

Supplementary Figure 12. EDS results for heterometallic MOFs and Fe3-MOFs **(CPM-161-163)** reported in this work.

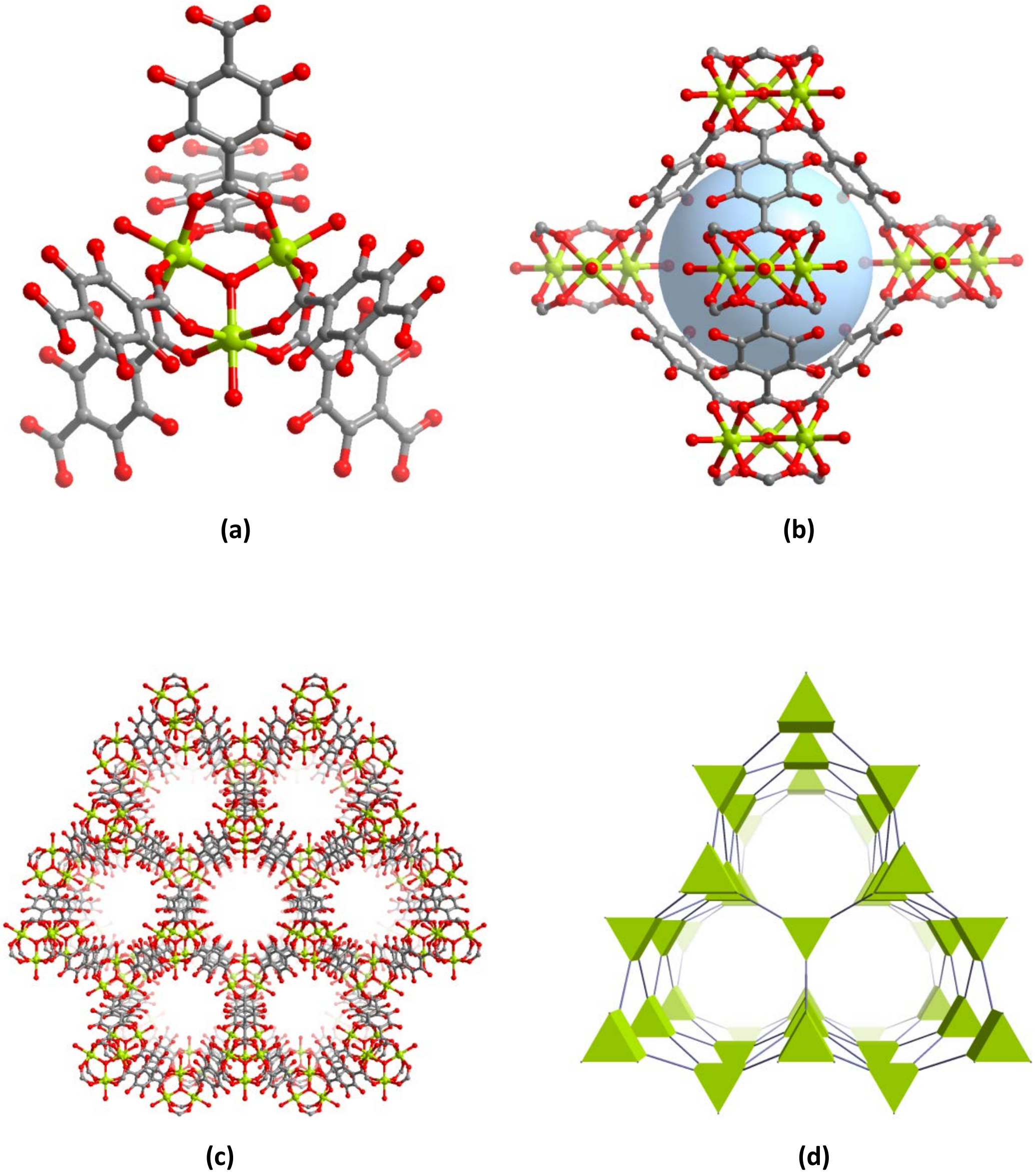

(a) (b) (c) (d)

Supplementary Figure 13. (a) The distribution of DHBDC around each trimeric SBU in **Mg3-MIL-88 (CPM-140)** and **Mg2V-MIL-88 (CPM-230)**. (b) Trigonal bipyramidal cage. (c) The 3D porous framework viewed along *c*-axis direction. (d) The schematic polyhedral drawing of **Mg3-MIL-88 (CPM-140)** and **Mg2V-Mil-88 (CPM-230)**.

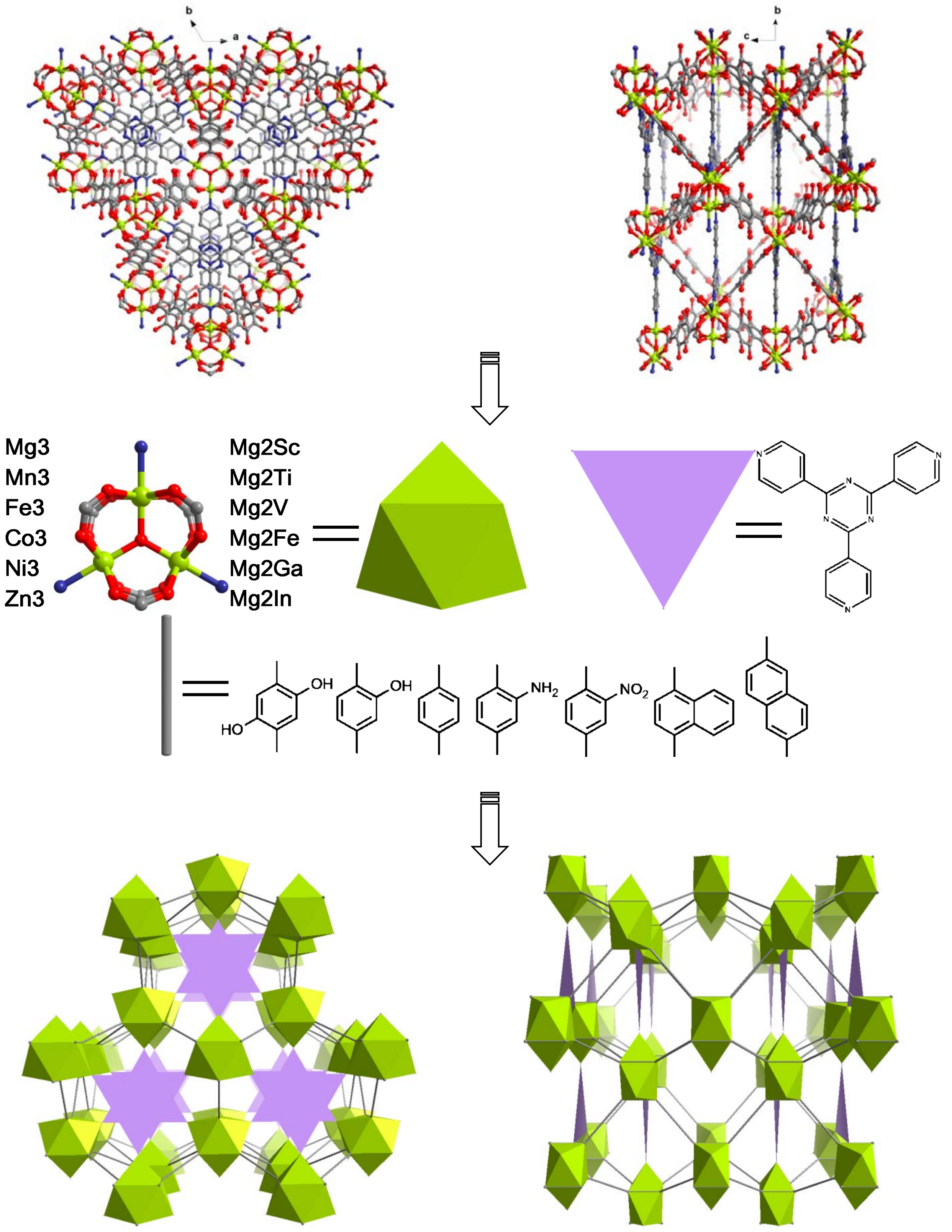

Supplementary Figure 14. The 3D porous framework and schematic polyhedral drawing of **[M₃(OH/O)(dicarboxylate)₃(TPT)]** *pacs* MOFs viewed along *a*- and c-axis directions, respectively.

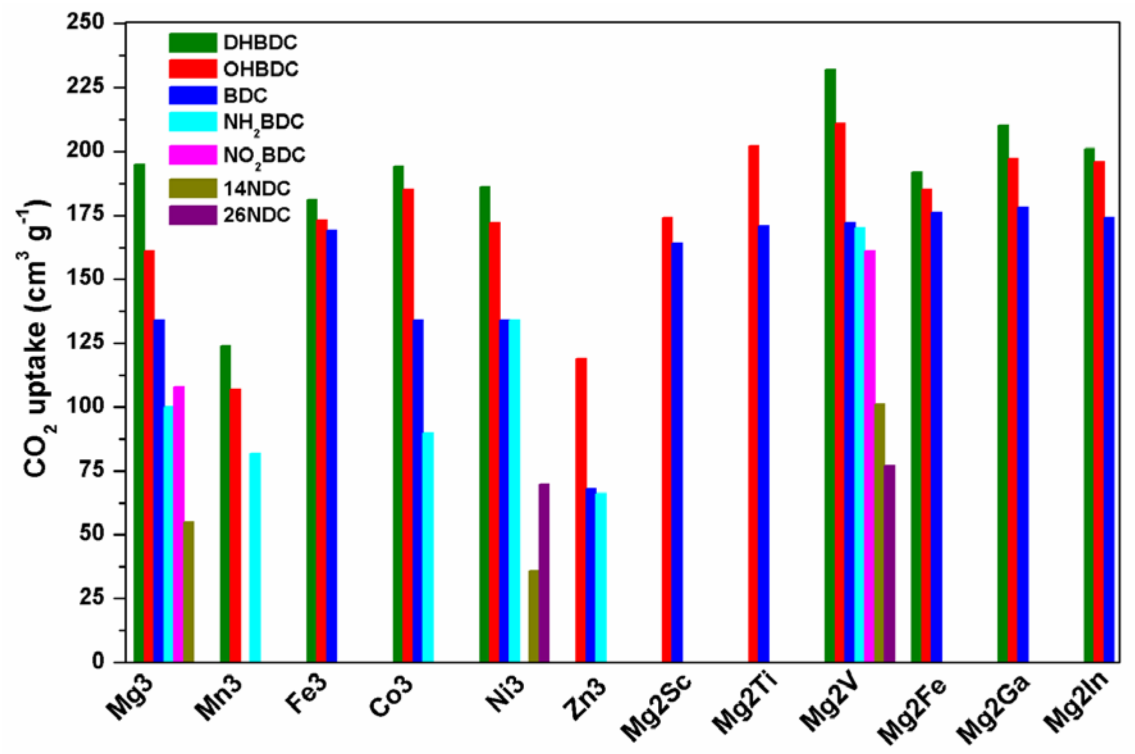

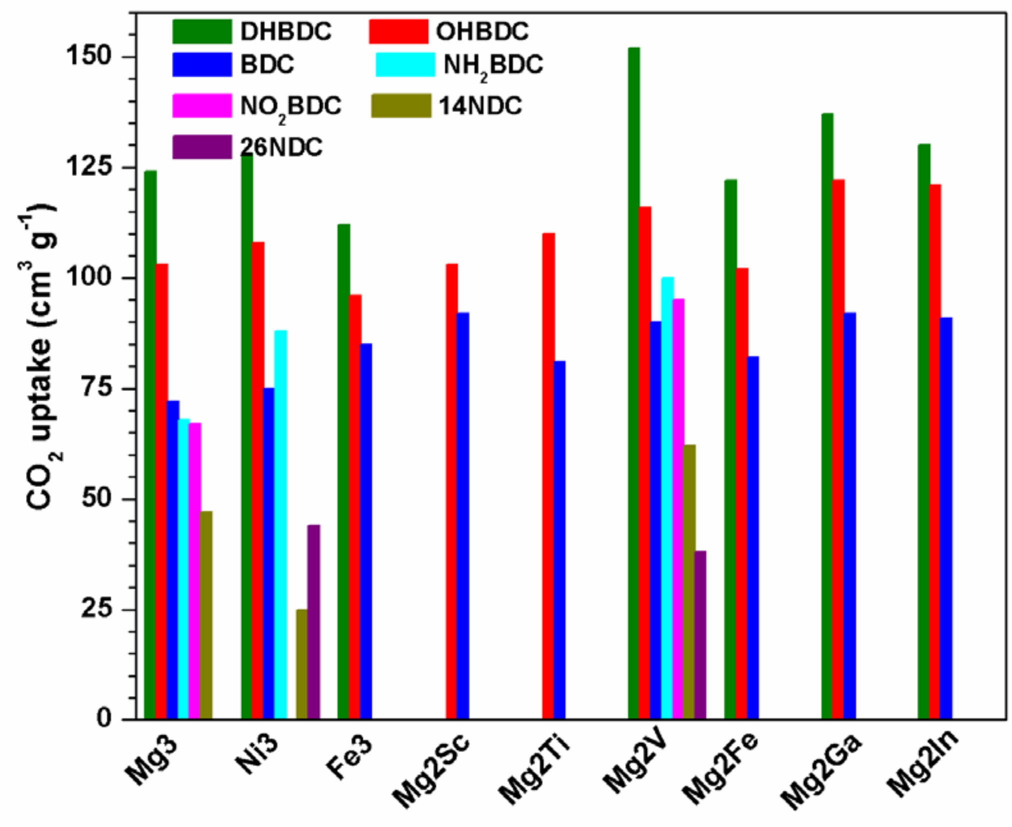

Supplementary Figure 15. CO_2 uptake performance summary for MOFs investigated in this work, Top: 273 K and 1 atm; Bottom: 298 K and 1 atm.

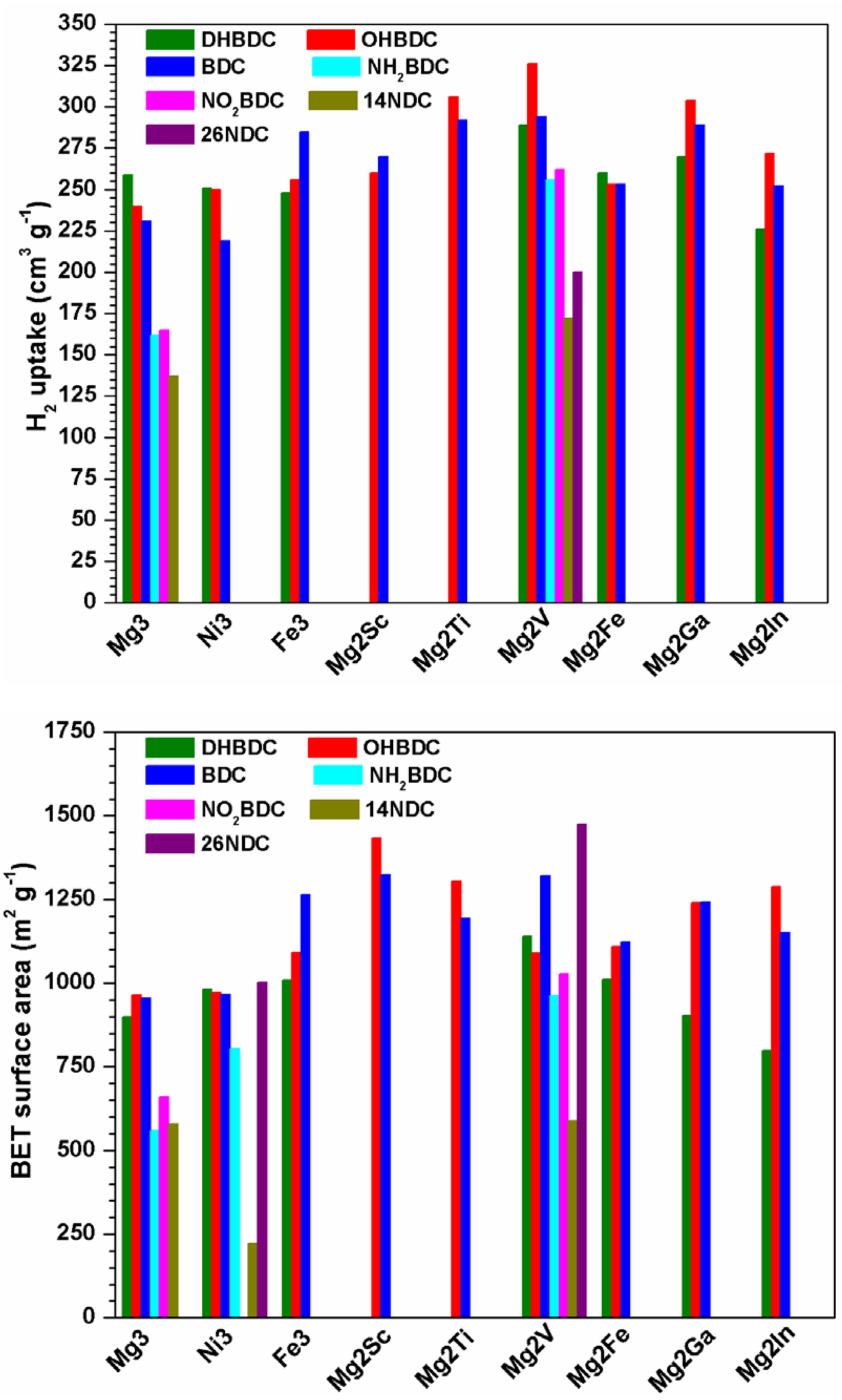

Supplementary Figure 16. H_2 uptake performance at 77 K and 1 atm (top) and BET surface area (bottom) summary for MOFs investigated in this work.

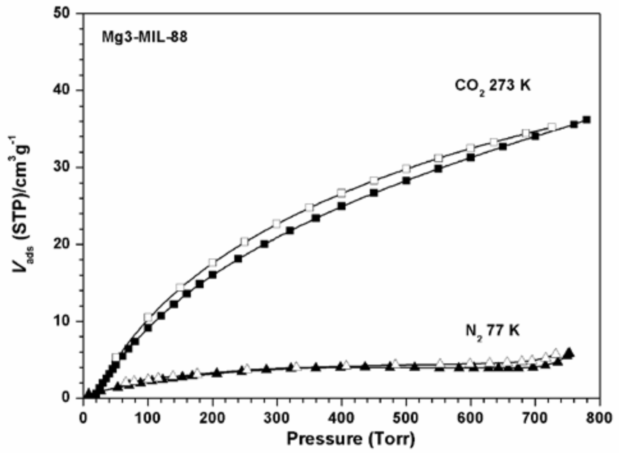

Supplementary Figure 17. CO_2 and N_2 adsorption isotherms for **Mg3-MIL-88 (CPM-140)**.

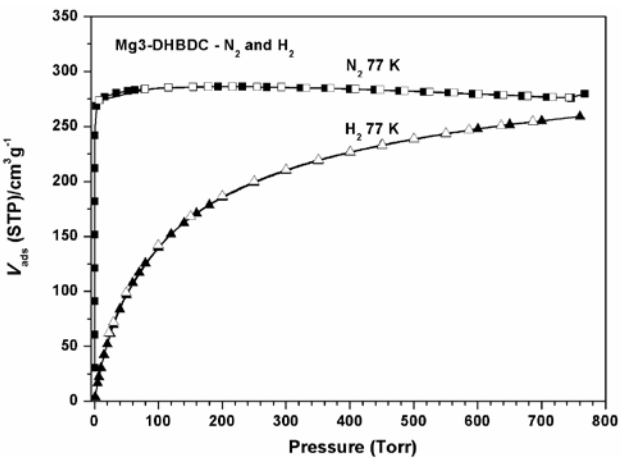

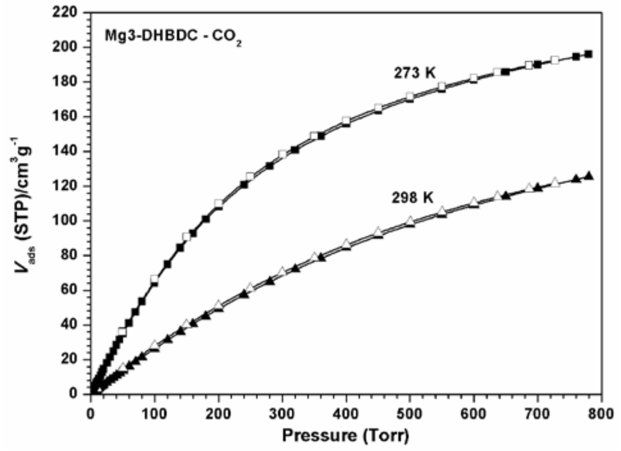

Supplementary Figure 18. N_2, H_2, and CO_2 adsorption isotherms for **Mg3-DHBDC (CPM-141)**.

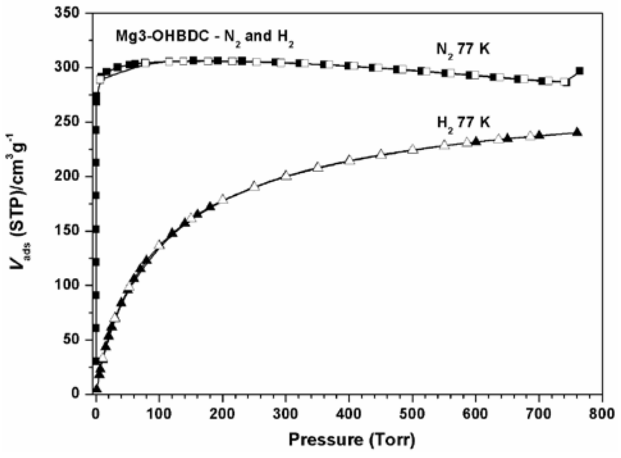

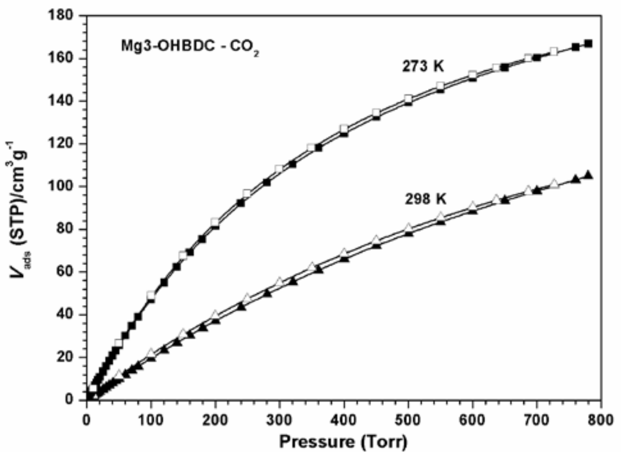

Supplementary Figure 19. N_2, H_2, and CO_2 adsorption isotherms for **Mg3-OHBDC (CPM-142)**.

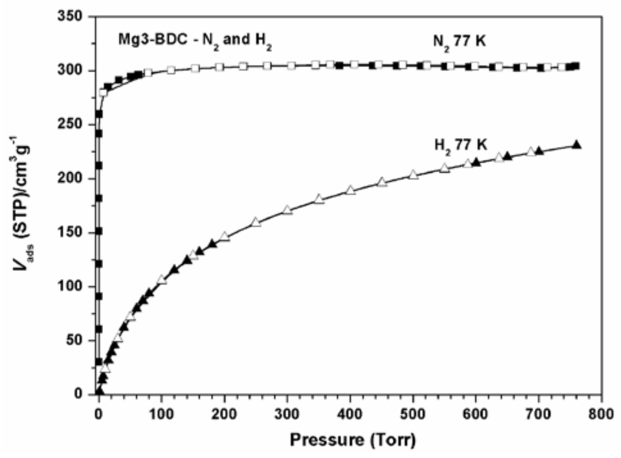

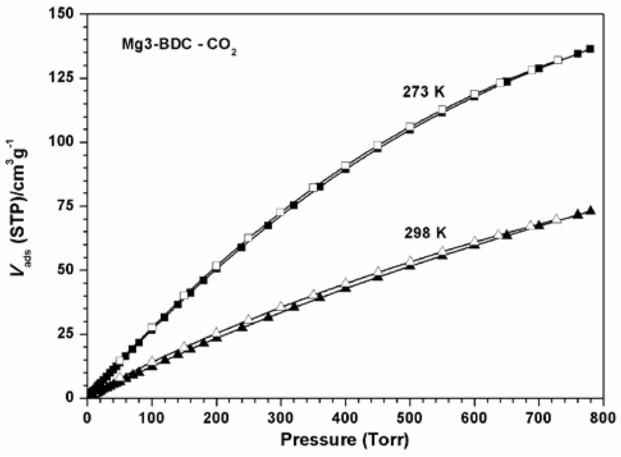

Supplementary Figure 20. N_2, H_2, and CO_2 adsorption isotherms for **Mg3-BDC (CPM-143)**.

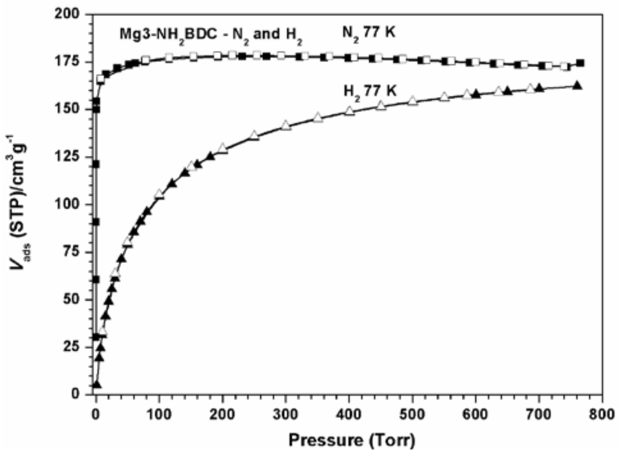

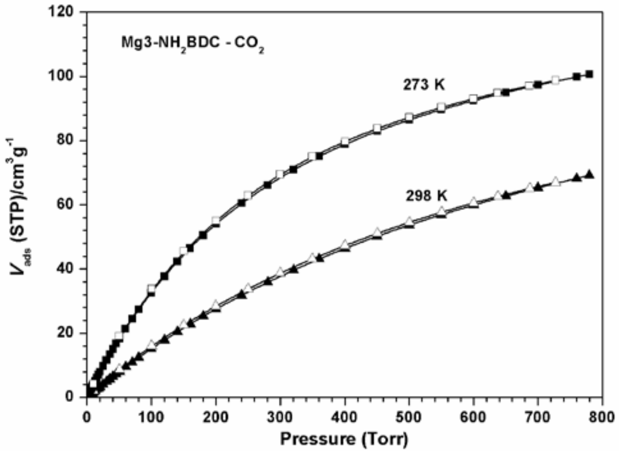

Supplementary Figure 21. N_2, H_2, and CO_2 adsorption isotherms for **Mg3-NH₂BDC (CPM-144)**.

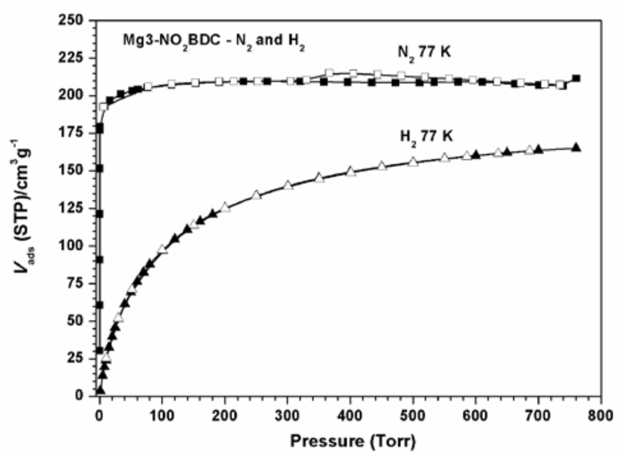

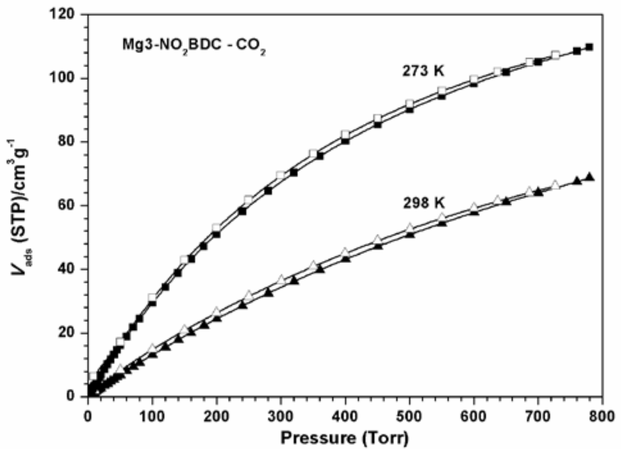

Supplementary Figure 22. N_2, H_2, and CO_2 adsorption isotherms for **Mg3-NO2BDC (CPM-145)**.

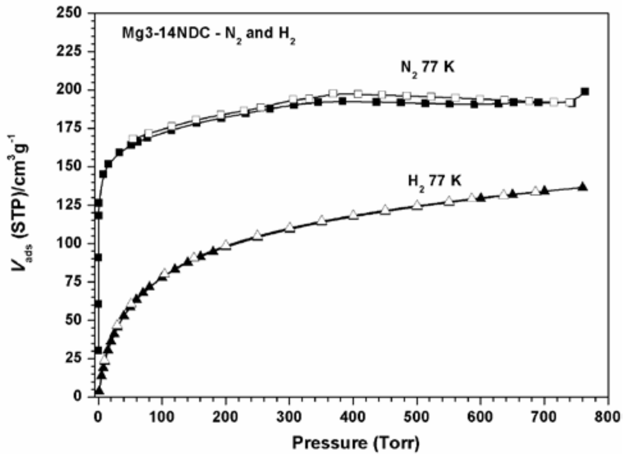

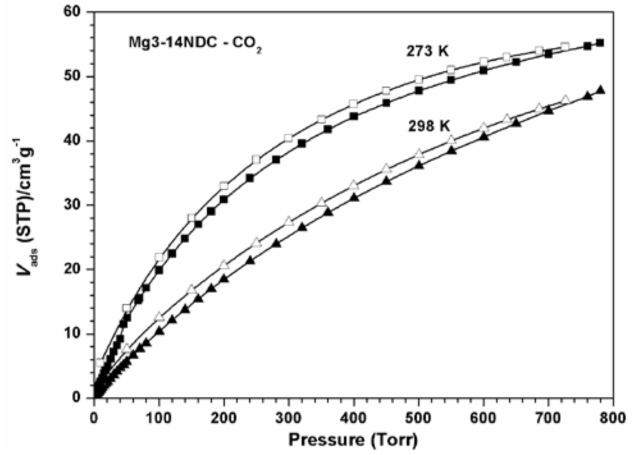

Supplementary Figure 23. N_2, H_2, and CO_2 adsorption isotherms for **Mg3-14NDC (CPM-146)**.

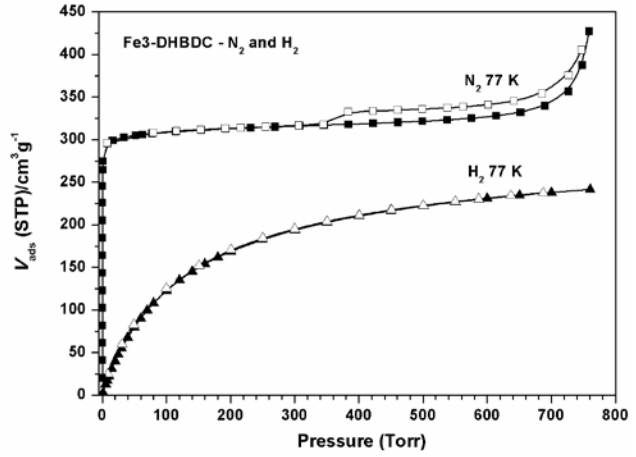

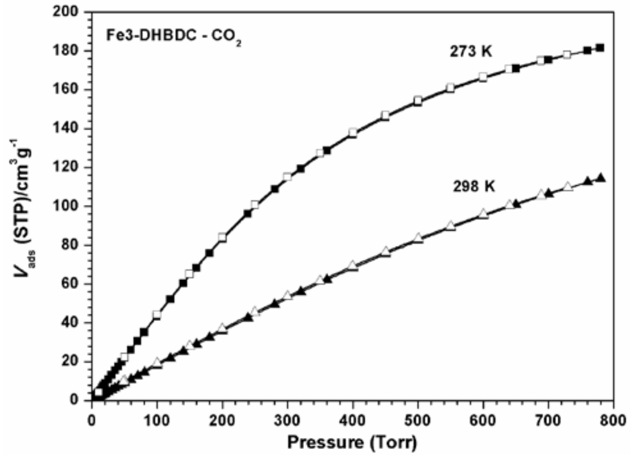

Supplementary Figure 24. N_2, H_2, and CO_2 adsorption isotherms for **Fe3-DHBDC (CPM-161)**.

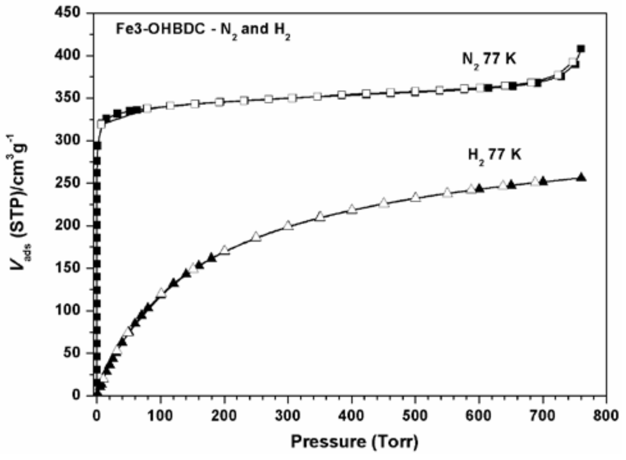

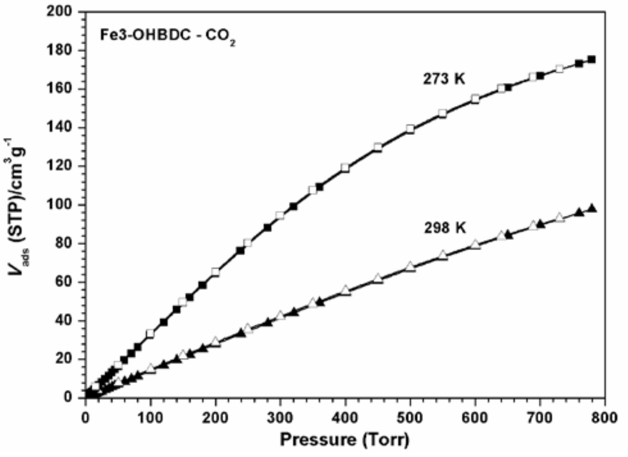

Supplementary Figure 25. N_2, H_2, and CO_2 adsorption isotherms for **Fe3-OHBDC (CPM-162)**.

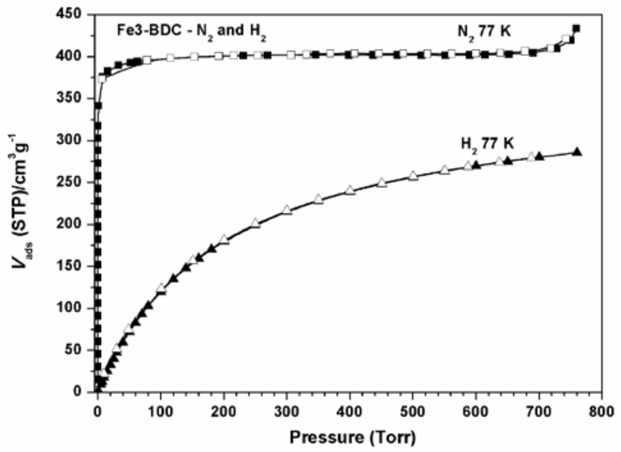

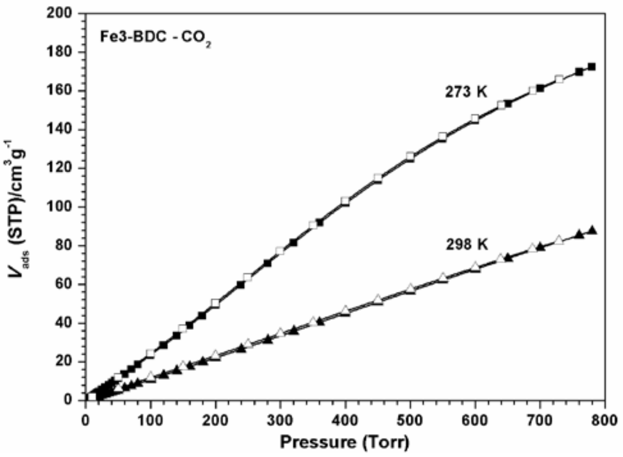

Supplementary Figure 26. N_2, H_2, and CO_2 adsorption isotherms for **Fe3-BDC (CPM-163)**.

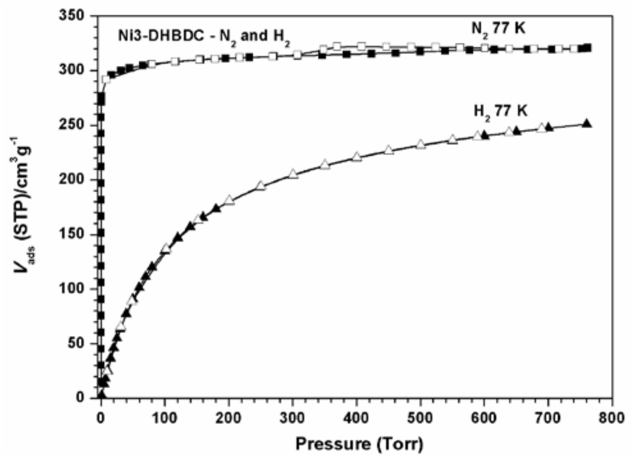

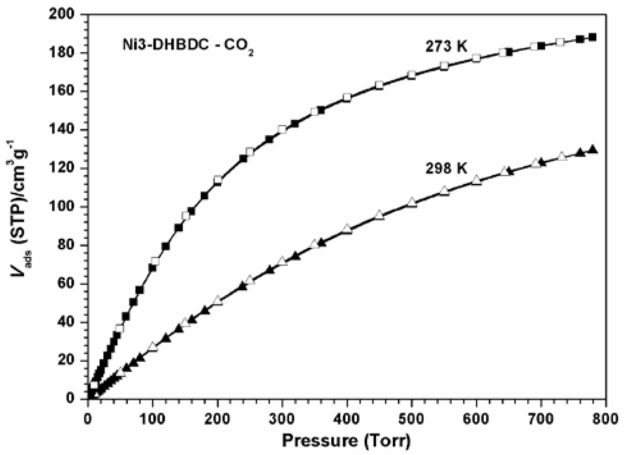

Supplementary Figure 27. N_2, H_2, and CO_2 adsorption isotherms for **Ni3-DHBDC (CPM-33b)**.

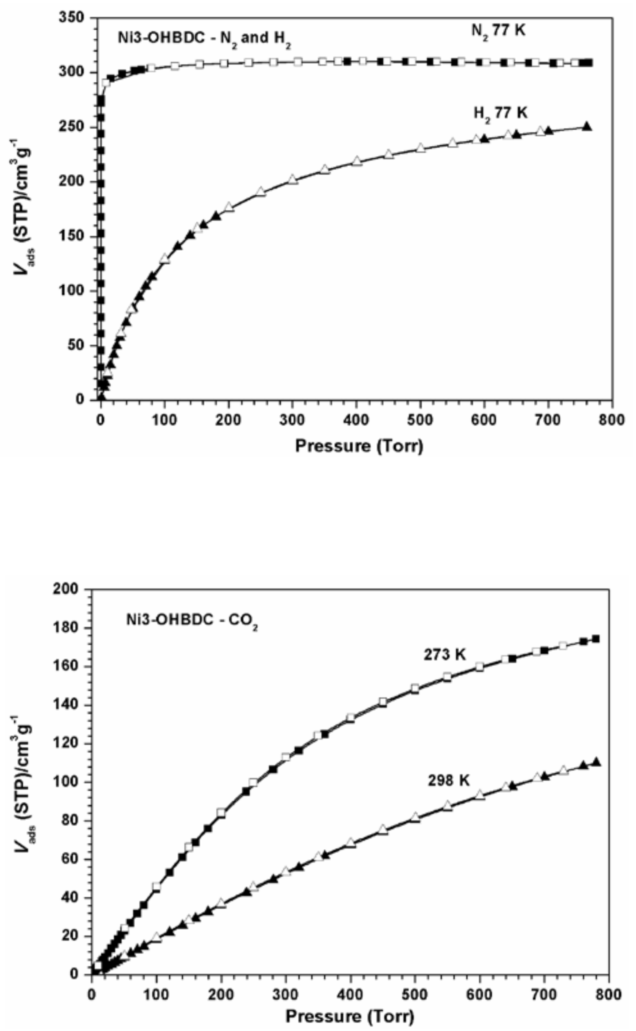

Supplementary Figure 28. N_2, H_2, and CO_2 adsorption isotherms for **Ni3-OHBDC (CPM-182)**.

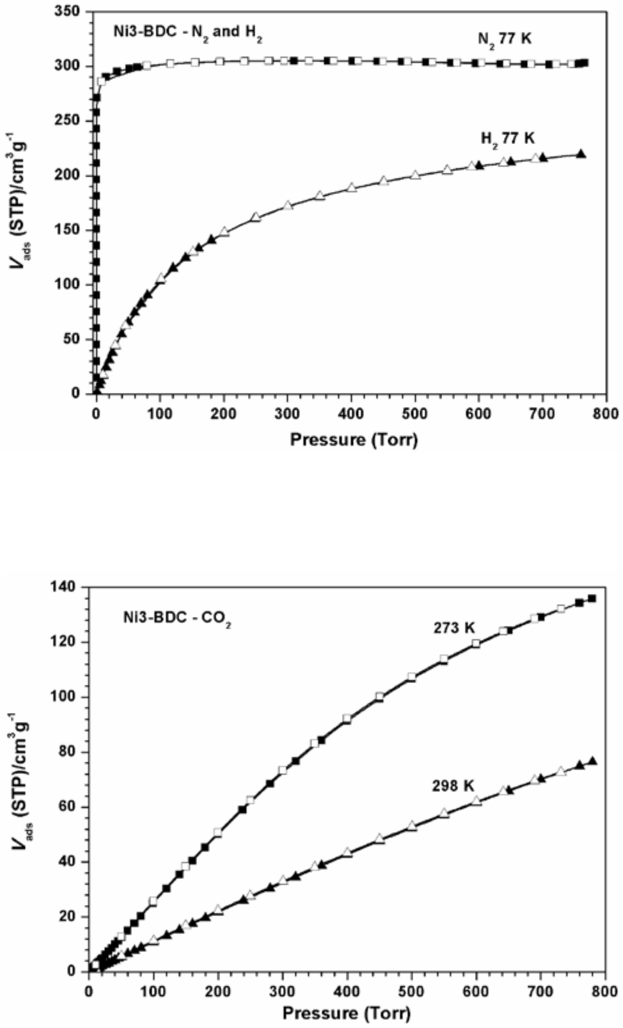

Supplementary Figure 29. N_2, H_2, and CO_2 adsorption isotherms for **Ni3-BDC (CPM-33a)**.

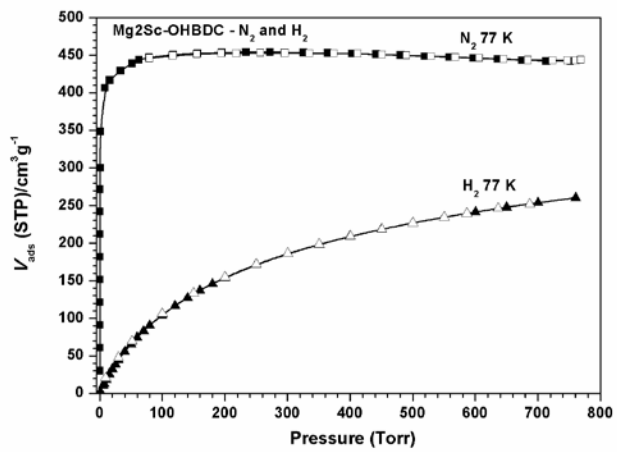

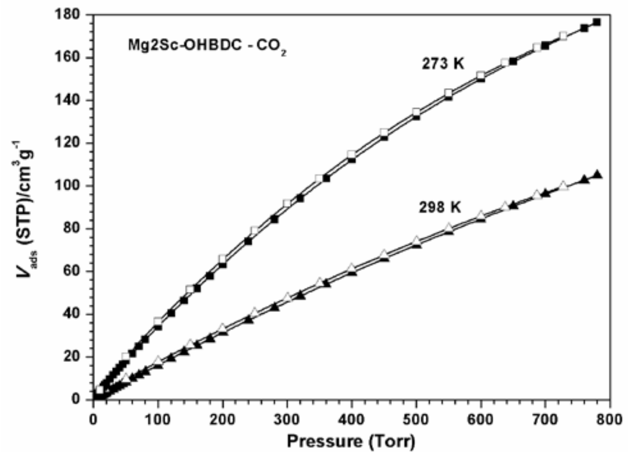

Supplementary Figure 30. N_2, H_2, and CO_2 adsorption isotherms for **Mg2Sc-OHBDC (CPM-212)**.

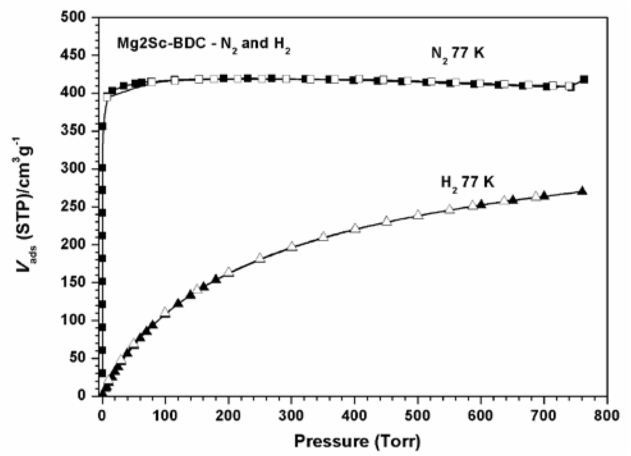

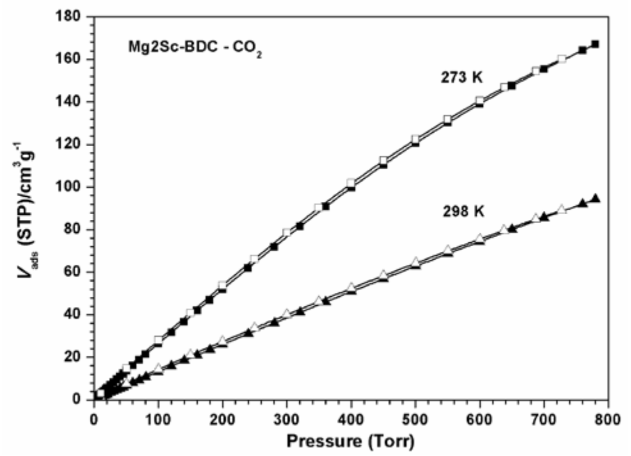

Supplementary Figure 31. N_2, H_2, and CO_2 adsorption isotherms for **Mg2Sc-BDC (CPM-213)**.

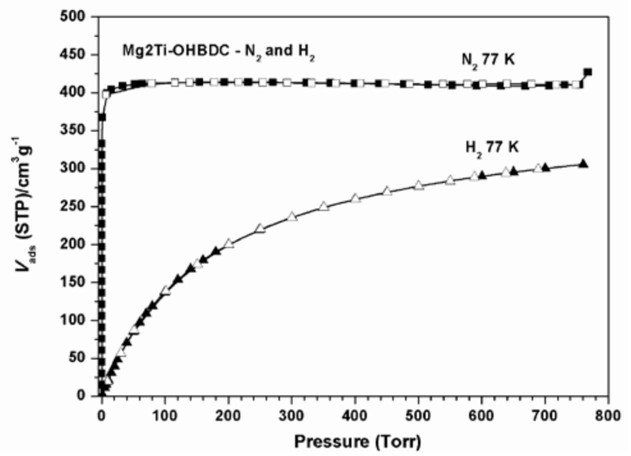

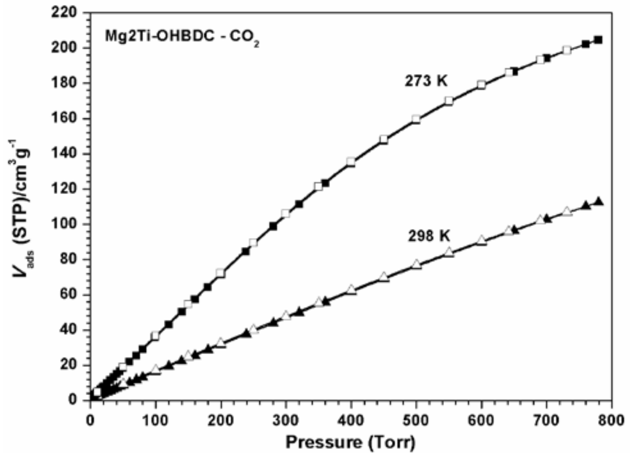

Supplementary Figure 32. N_2, H_2, and CO_2 adsorption isotherms for **Mg2Ti-OHBDC (CPM-222)**.

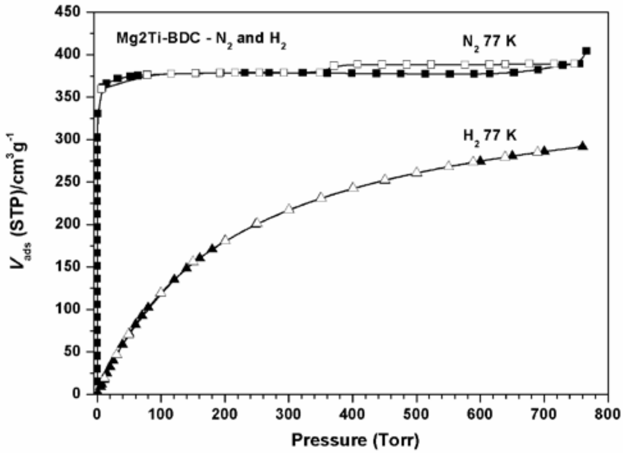

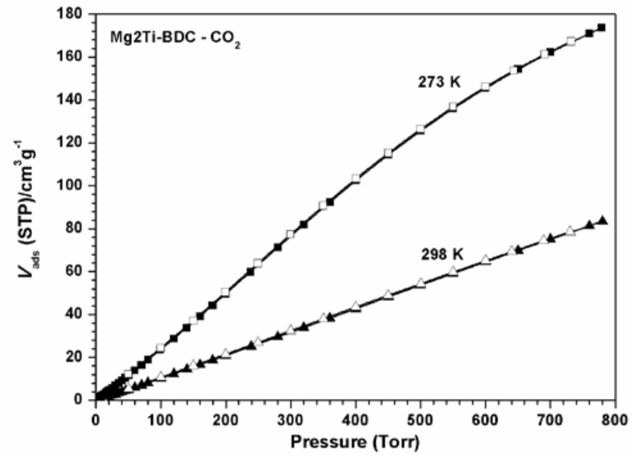

Supplementary Figure 33. N_2, H_2, and CO_2 adsorption isotherms for **Mg2Ti-BDC (CPM-223)**.

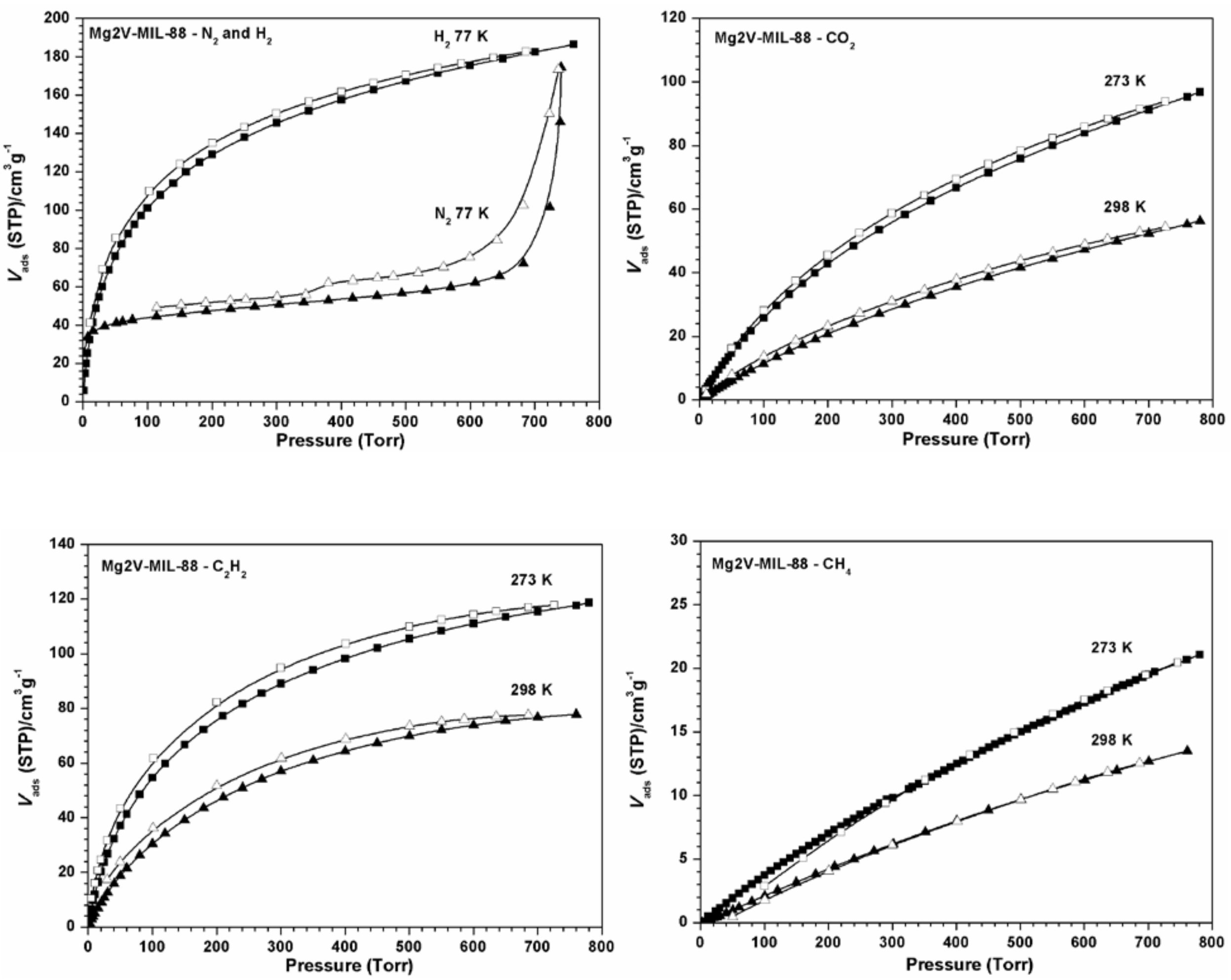

Supplementary Figure 34. N_2, H_2, CO_2 CH_4, and C_2H_2 adsorption isotherms for **Mg2V-MIL-88 (CPM-230)**.

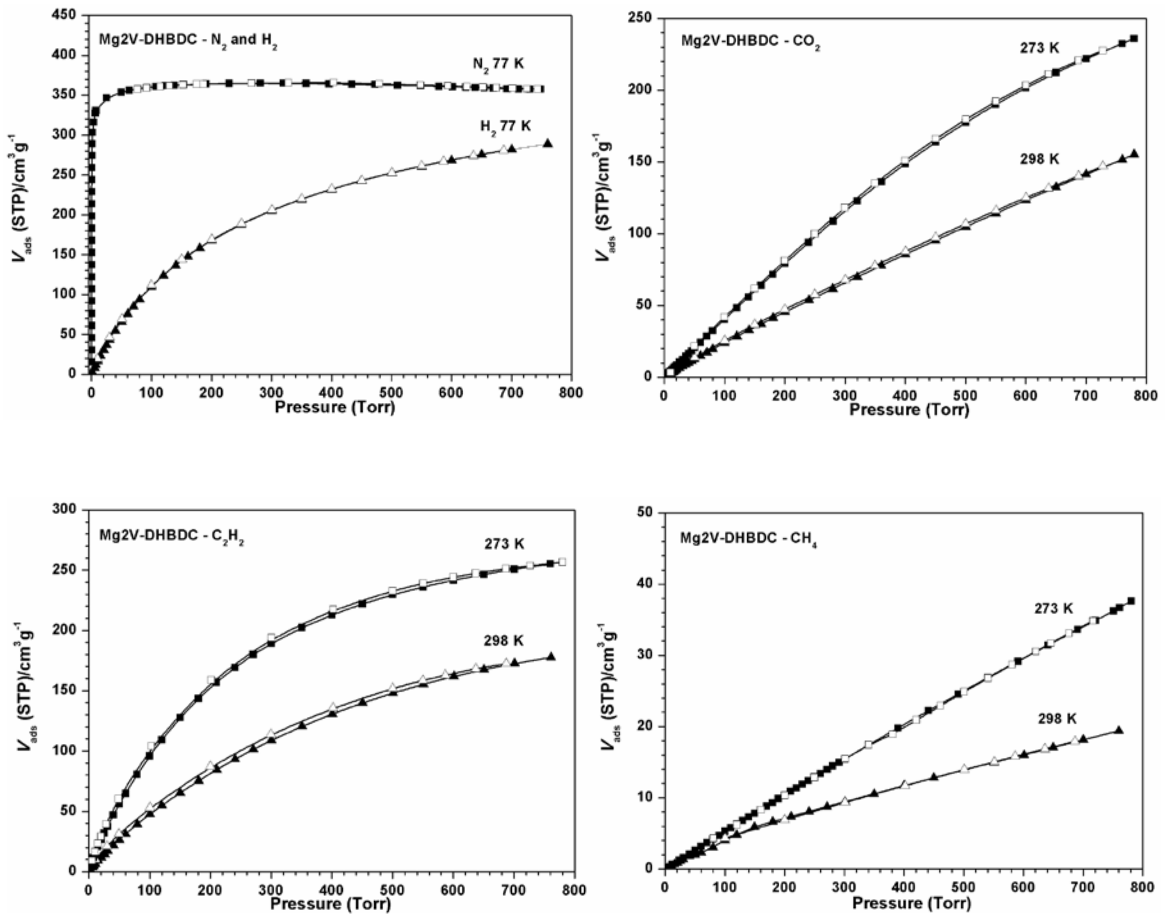

Supplementary Figure 35. N_2, H_2, CO_2 CH_4, and C_2H_2 adsorption isotherms for **Mg2V-DHBDC (CPM-231)**.

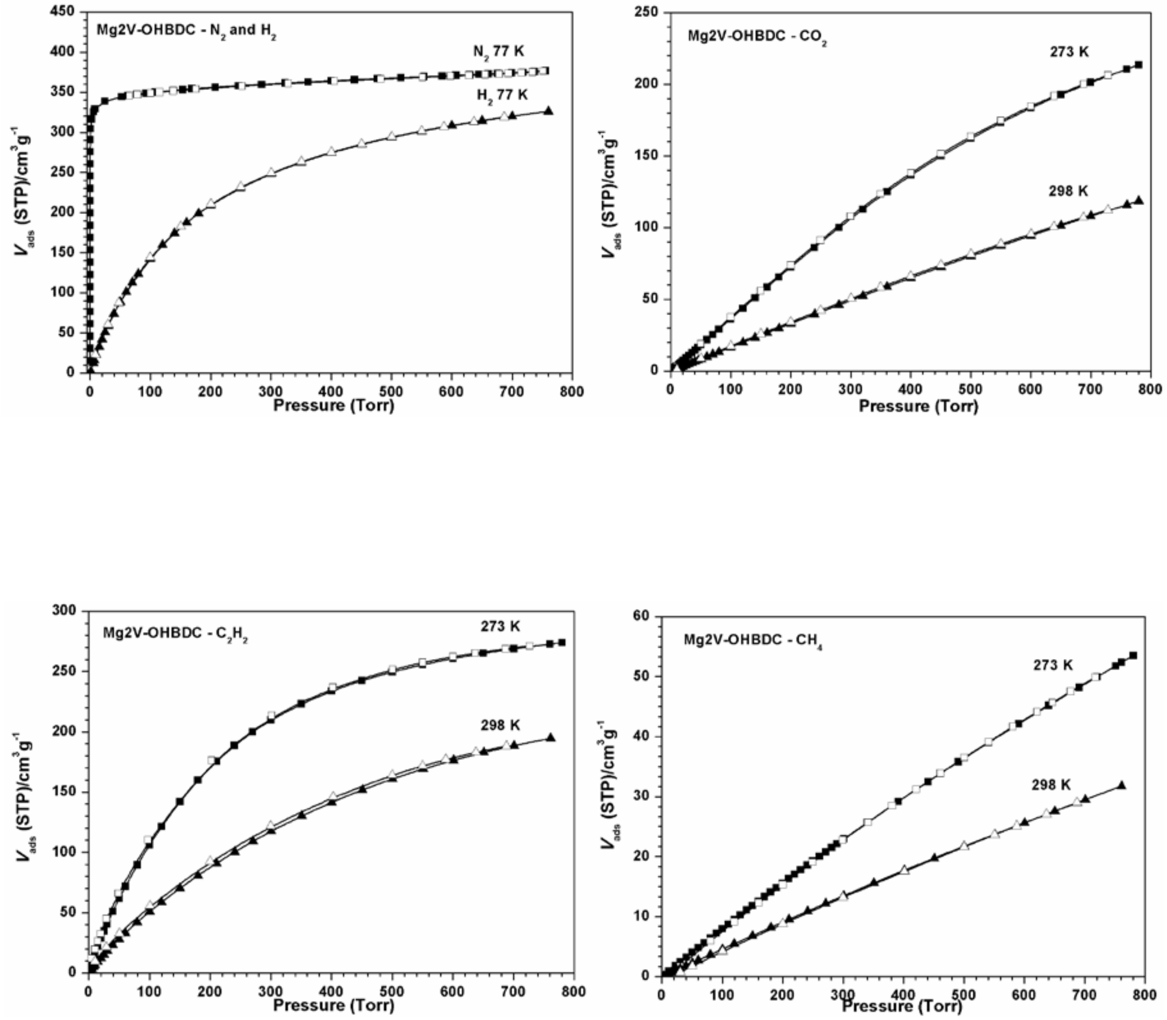

Supplementary Figure 36. N_2, H_2, CO_2 CH_4, and C_2H_2 adsorption isotherms for **Mg2V-OHBDC (CPM-232)**.

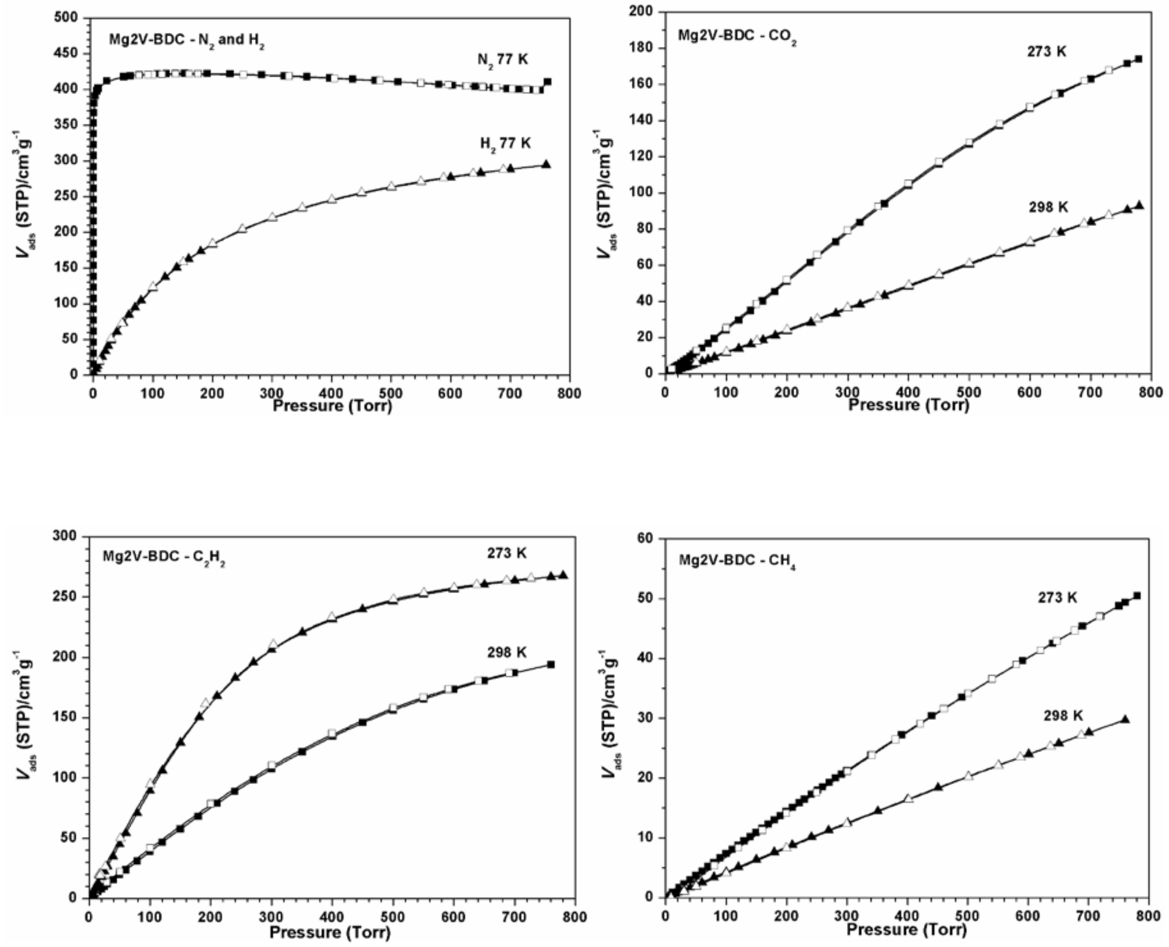

Supplementary Figure 37. N_2, H_2, CO_2 CH_4, and C_2H_2 adsorption isotherms for **Mg2V-BDC (CPM-233)**.

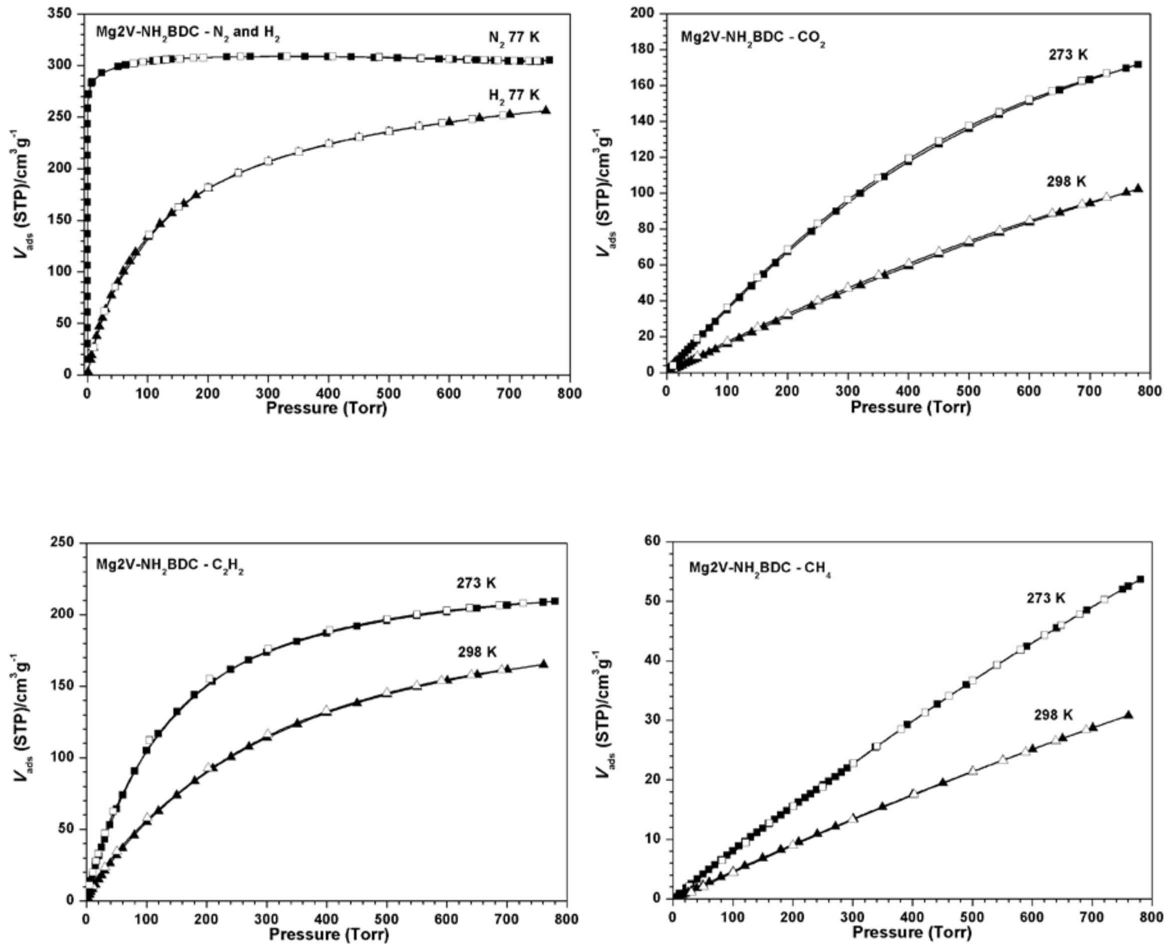

Supplementary Figure 38. N_2, H_2, CO_2 CH_4, and C_2H_2 adsorption isotherms for **Mg2V-NH$_2$BDC (CPM-234)**.

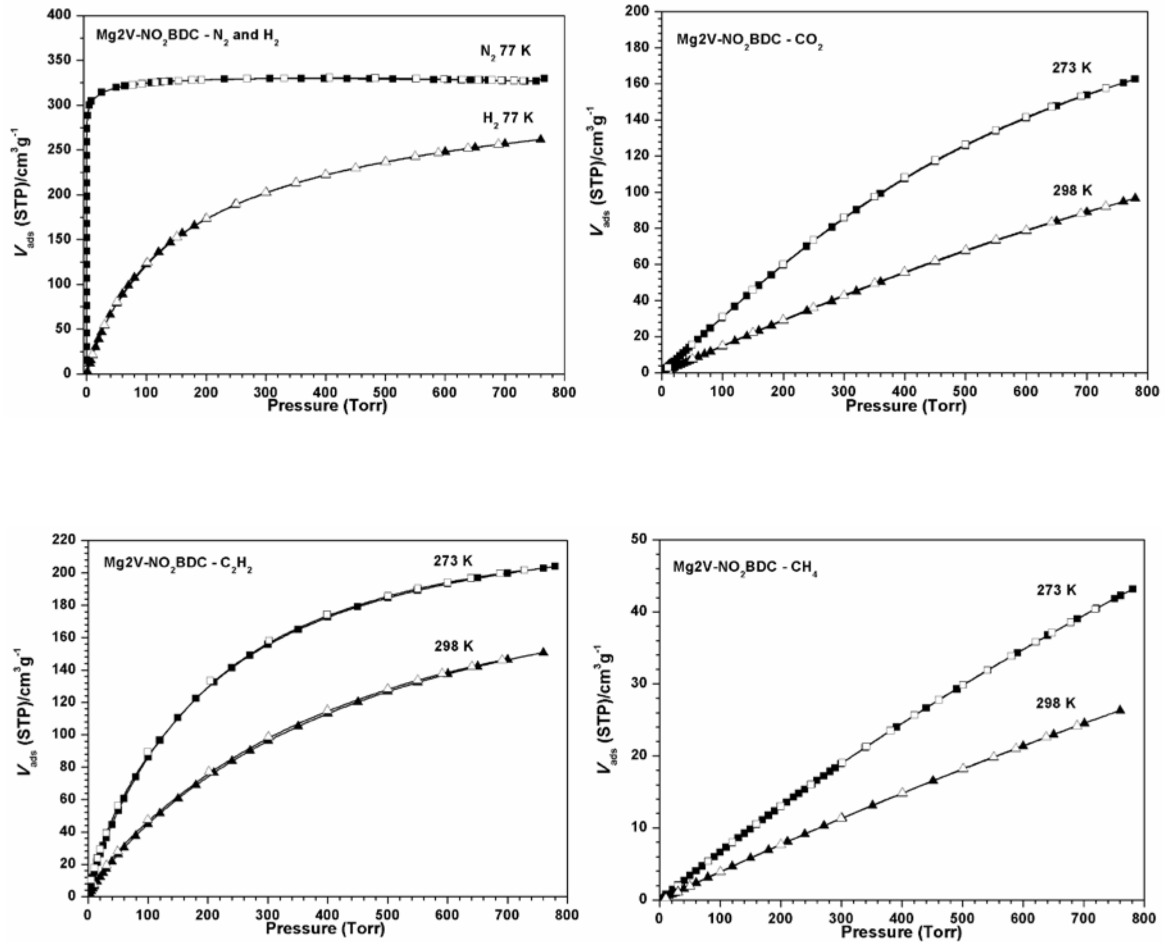

Supplementary Figure 39. N_2, H_2, CO_2 CH_4, and C_2H_2 adsorption isotherms for **Mg2V-NO$_2$BDC (CPM-235)**.

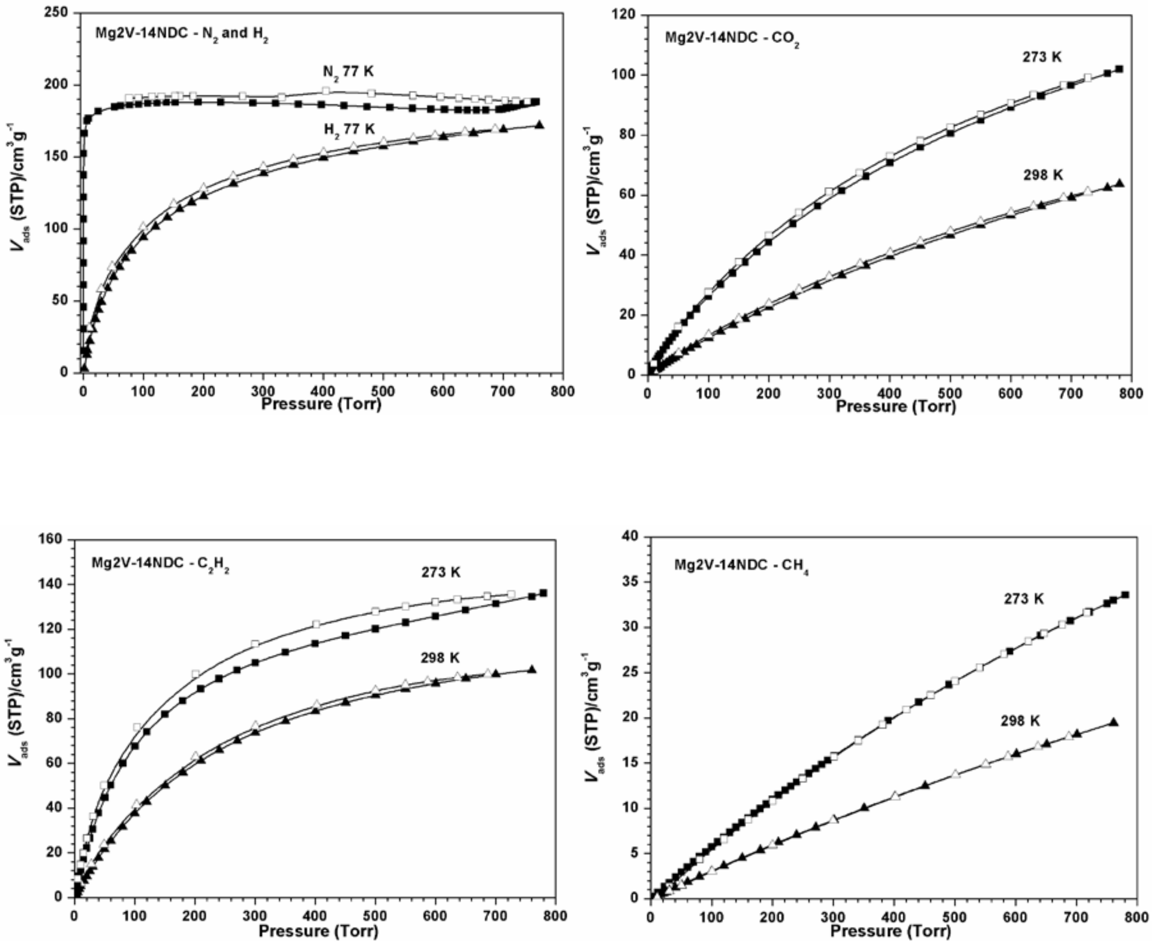

Supplementary Figure 40. N_2, H_2, CO_2 CH_4, and C_2H_2 adsorption isotherms for **Mg2V-14NDC (CPM-236)**.

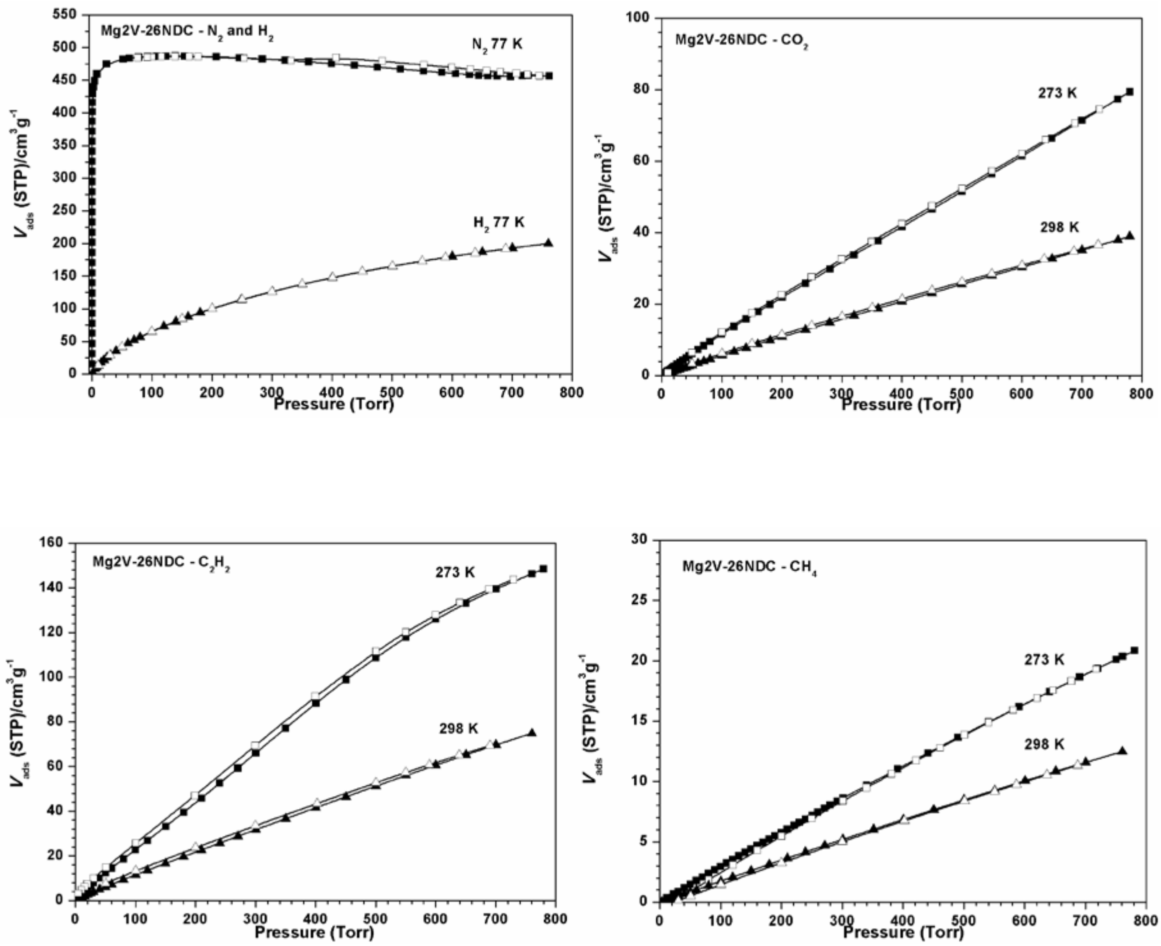

Supplementary Figure 41. N_2, H_2, CO_2 CH_4, and C_2H_2 adsorption isotherms for **Mg2V-26NDC (CPM-237)**.

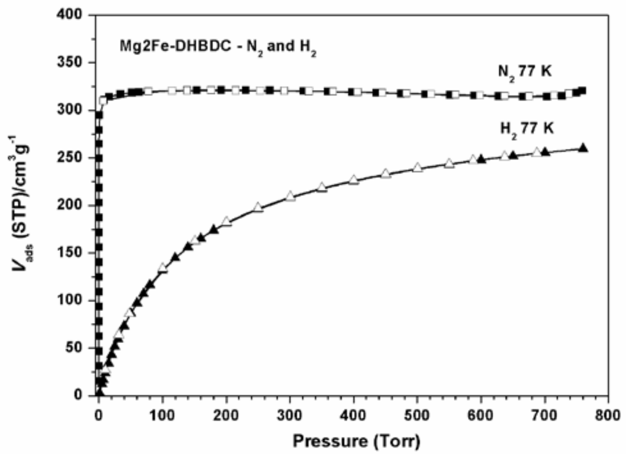

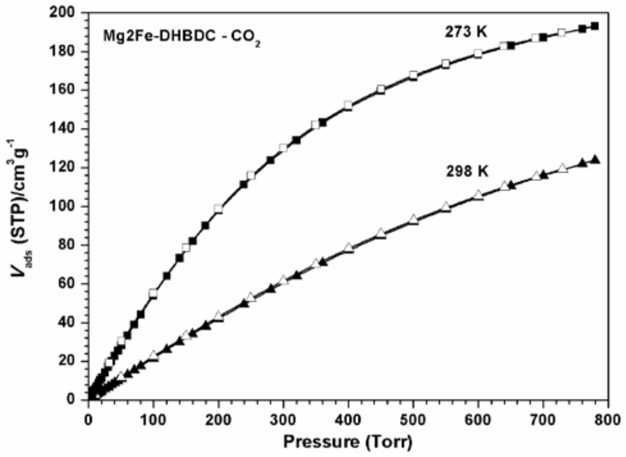

Supplementary Figure 42. N_2, H_2, and CO_2 adsorption isotherms for **Mg2Fe-DHBDC (CPM-261)**.

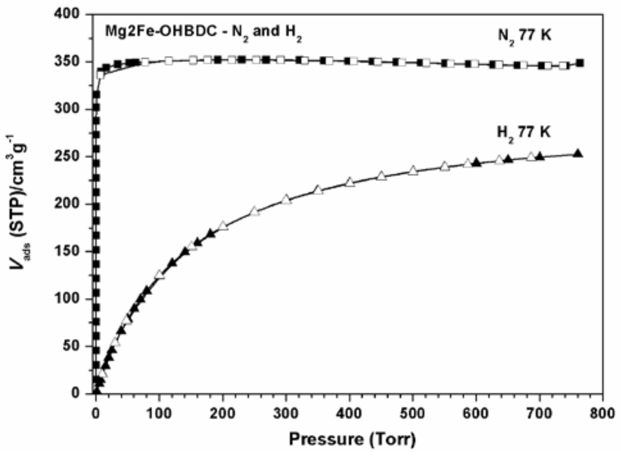

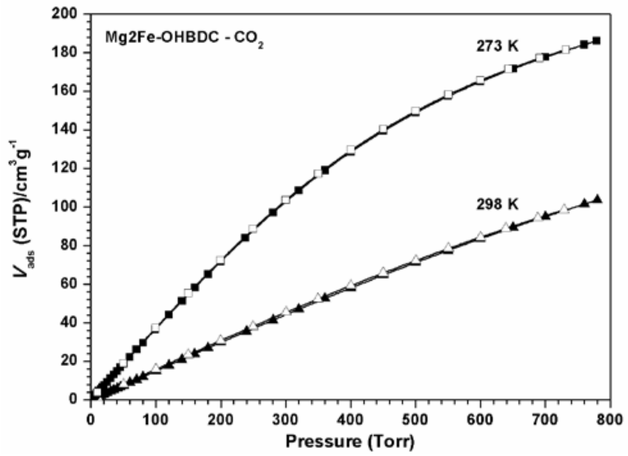

Supplementary Figure 43. N_2, H_2, and CO_2 adsorption isotherms for **Mg2Fe-OHBDC (CPM-262)**.

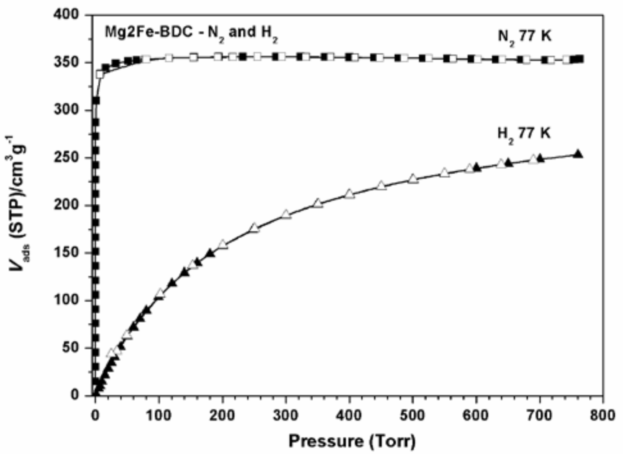

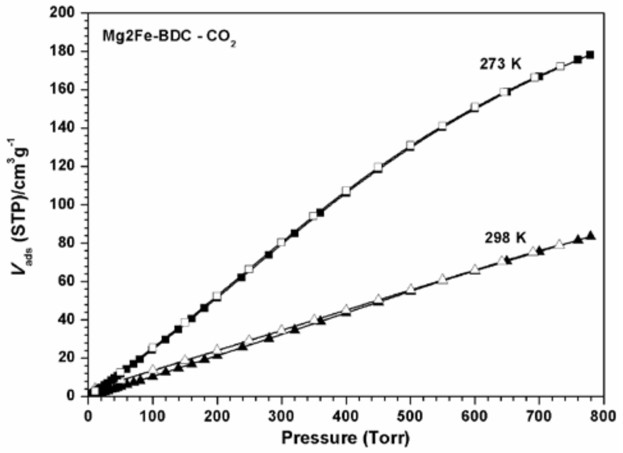

Supplementary Figure 44. N_2, H_2, and CO_2 adsorption isotherms for **Mg2Fe-BDC (CPM-263)**.

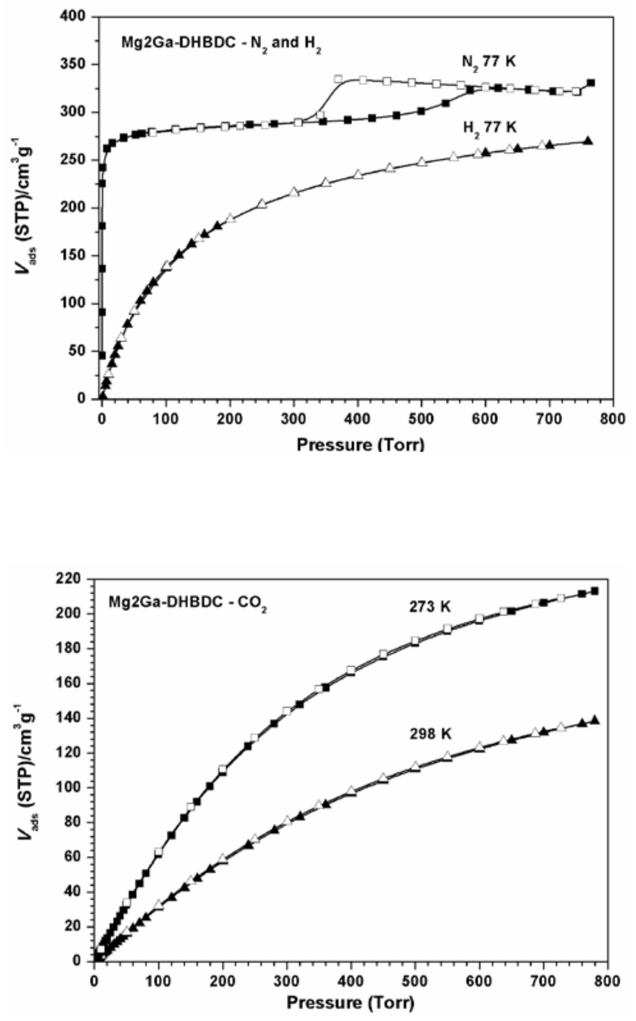

Supplementary Figure 45. N_2, H_2, and CO_2 adsorption isotherms for **Mg2Ga-DHBDC (CPM-271)**.

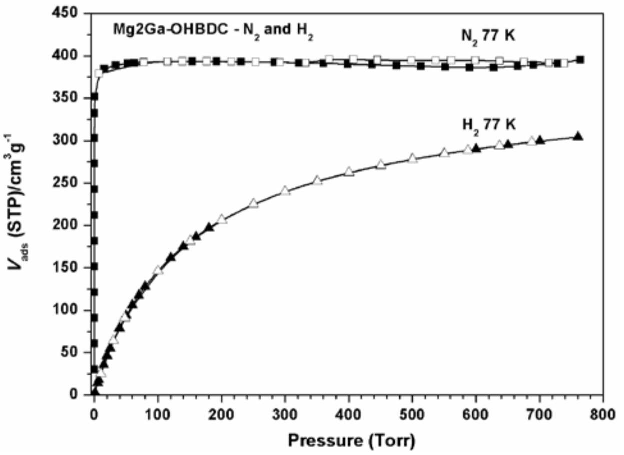

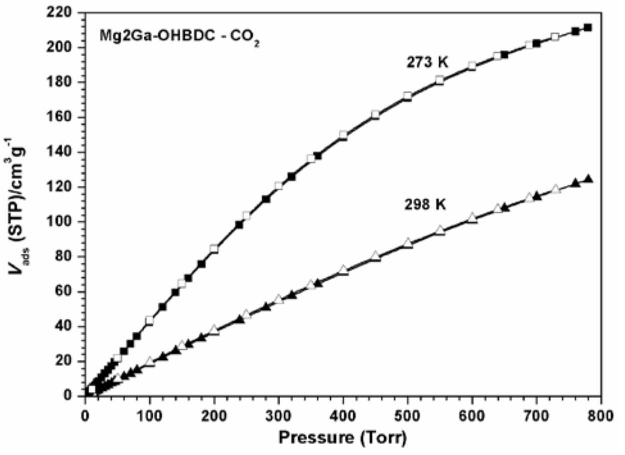

Supplementary Figure 46. N_2, H_2, and CO_2 adsorption isotherms for **Mg2Ga-OHBDC (CPM-272)**.

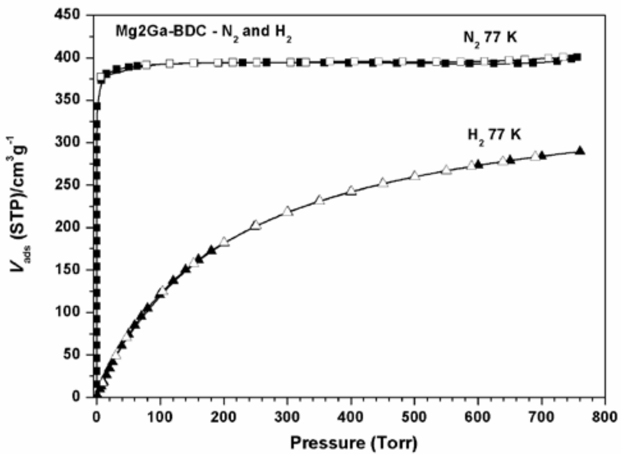

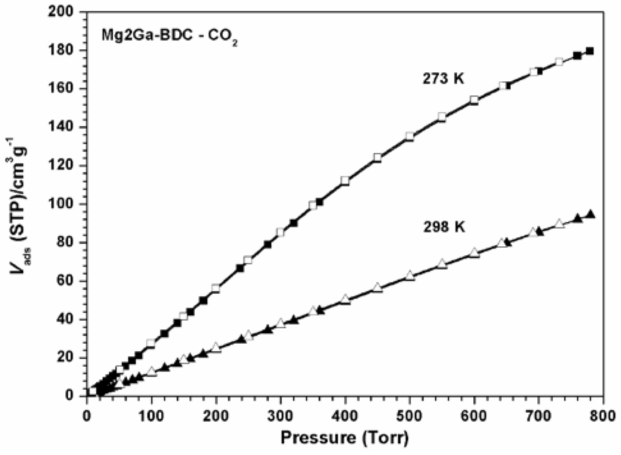

Supplementary Figure 47. N_2, H_2, and CO_2 adsorption isotherms for **Mg2Ga-BDC (CPM-273)**.

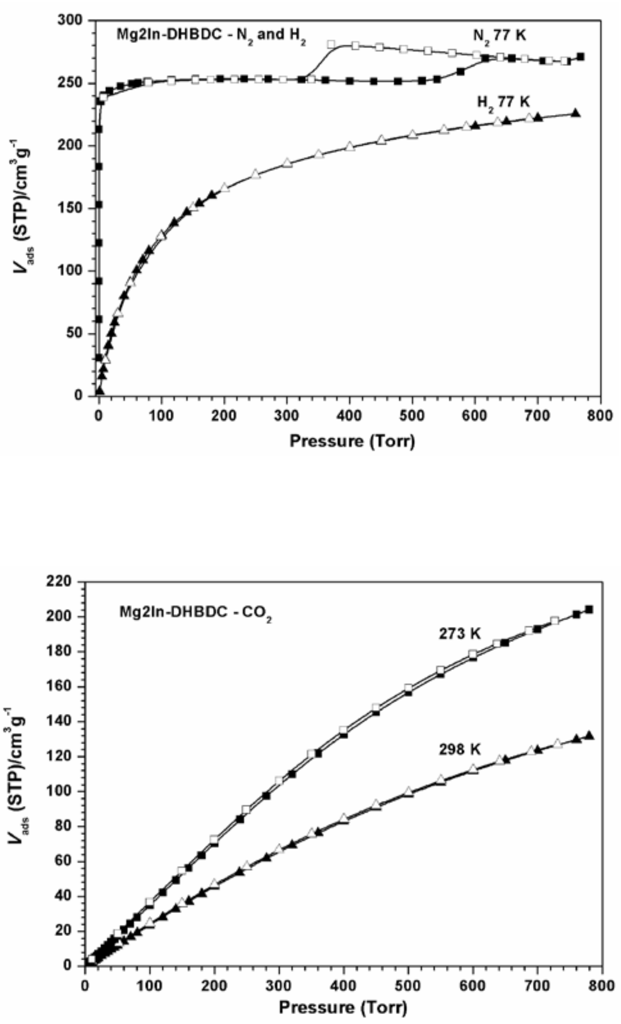

Supplementary Figure 48. N_2, H_2, and CO_2 adsorption isotherms for **Mg2In-DHBDC (CPM-291)**.

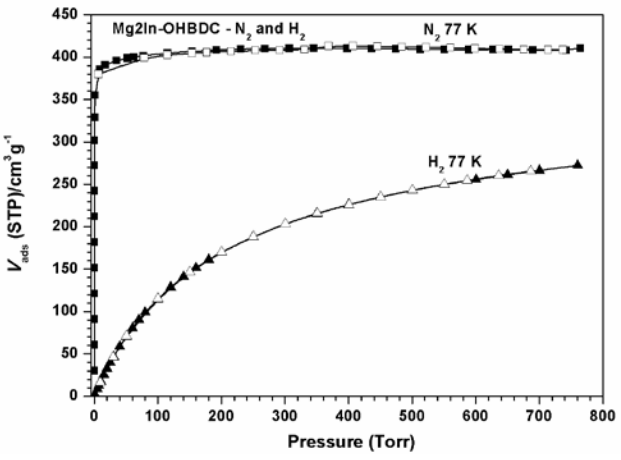

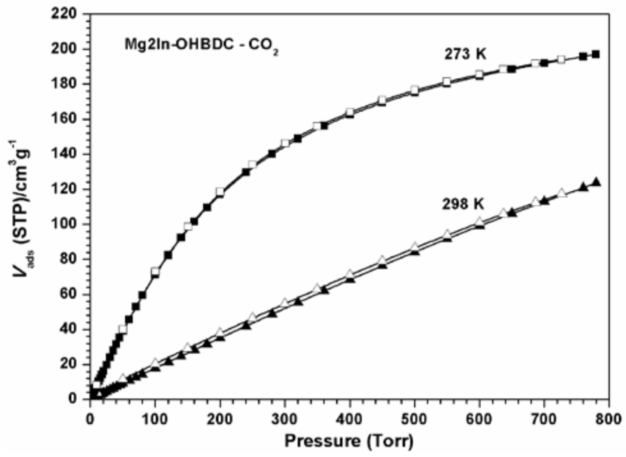

Supplementary Figure 49. N_2, H_2, and CO_2 adsorption isotherms for **Mg2In-OHBDC (CPM-292)**.

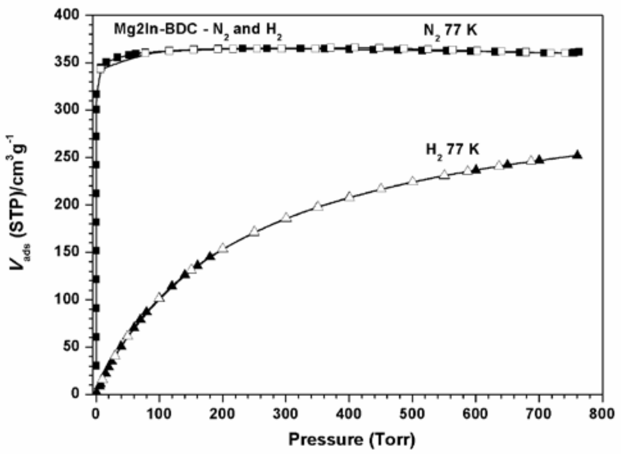

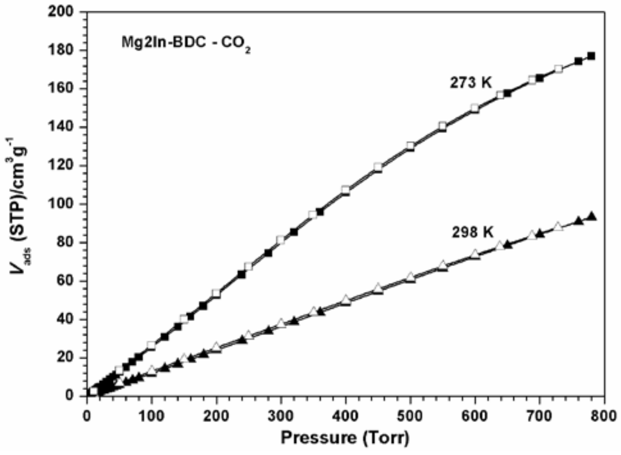

Supplementary Figure 50. N_2, H_2, and CO_2 adsorption isotherms for **Mg2In-BDC (CPM-293)**.

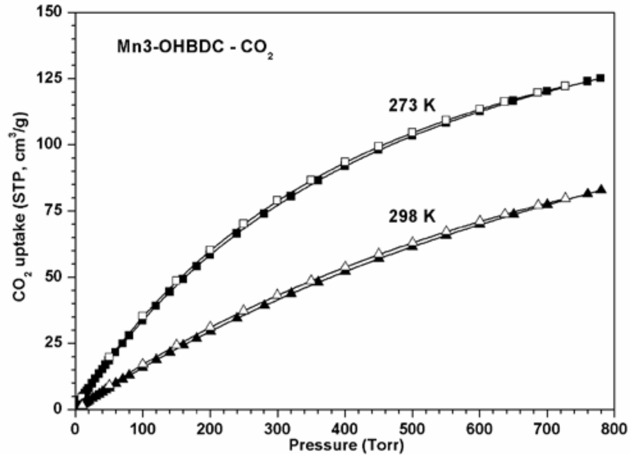

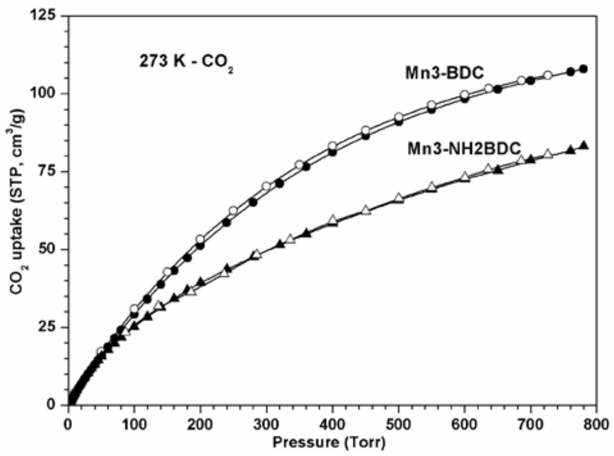

Supplementary Figure 51. CO_2 adsorption isotherms for **Mn3-OHBDC (CPM-152), Mn3-BDC (CPM-153)** and **Mn3-NH_2BDC (CPM-154).**

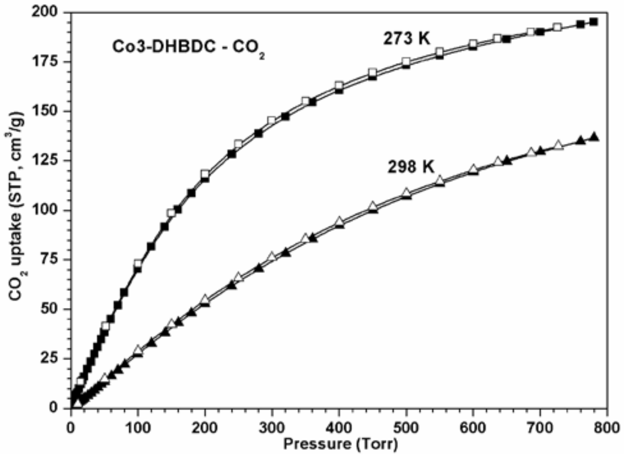

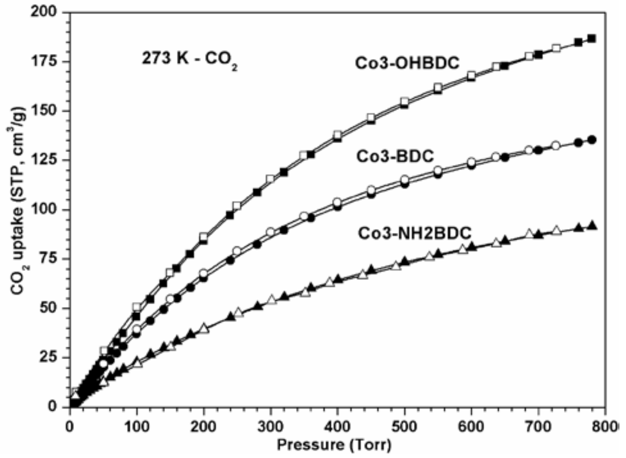

Supplementary Figure 52. CO_2 adsorption isotherms for **Co3-DHBDC (CPM-171), Co3-OHBDC (CPM-172), Co3-BDC (CPM-173)** and **Mn3-NH_2BDC (CPM-174).**

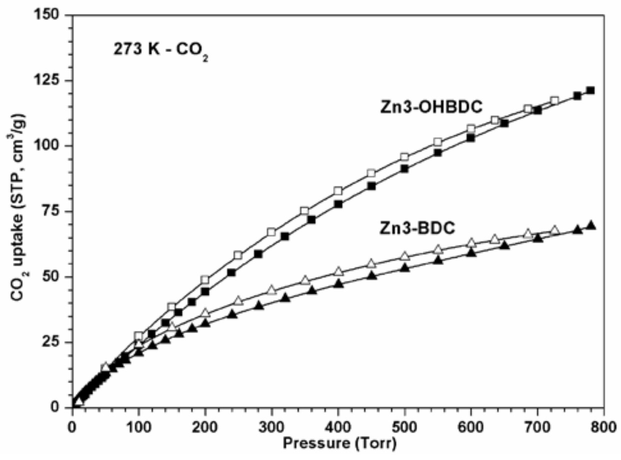

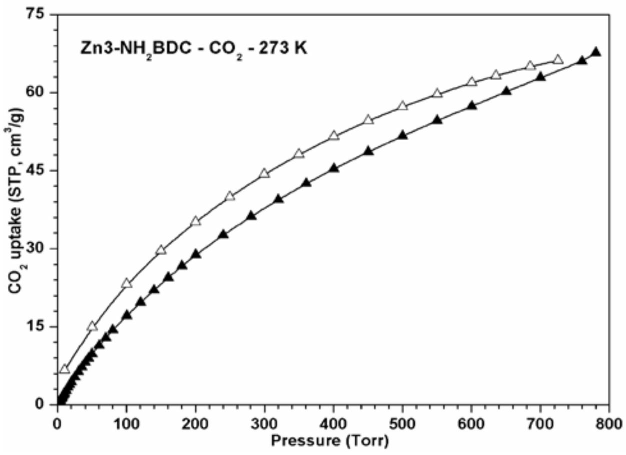

Supplementary Figure 53. CO_2 adsorption isotherms for **Zn3-OHBDC (CPM-192), Zn3-BDC (CPM-193)** and **Zn3-NH_2BDC (CPM-194).**

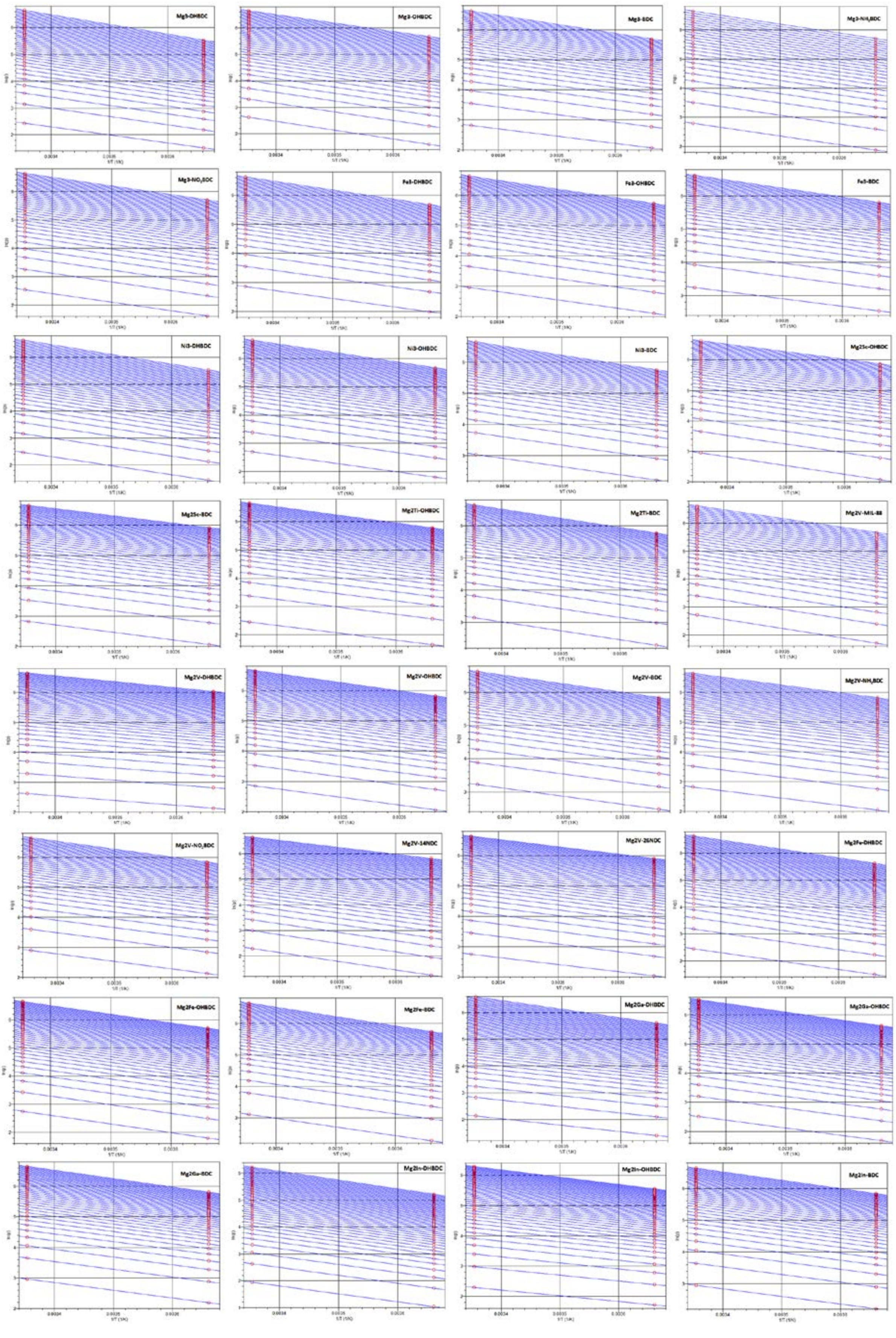

Supplementary Figure 54. CO_2 adsorption isostere plots for reported MOFs, confirming the accuracy of the Q_{st} determined from VT CO_2 adsorption isotherms as evidenced by the linearity in the isosters.

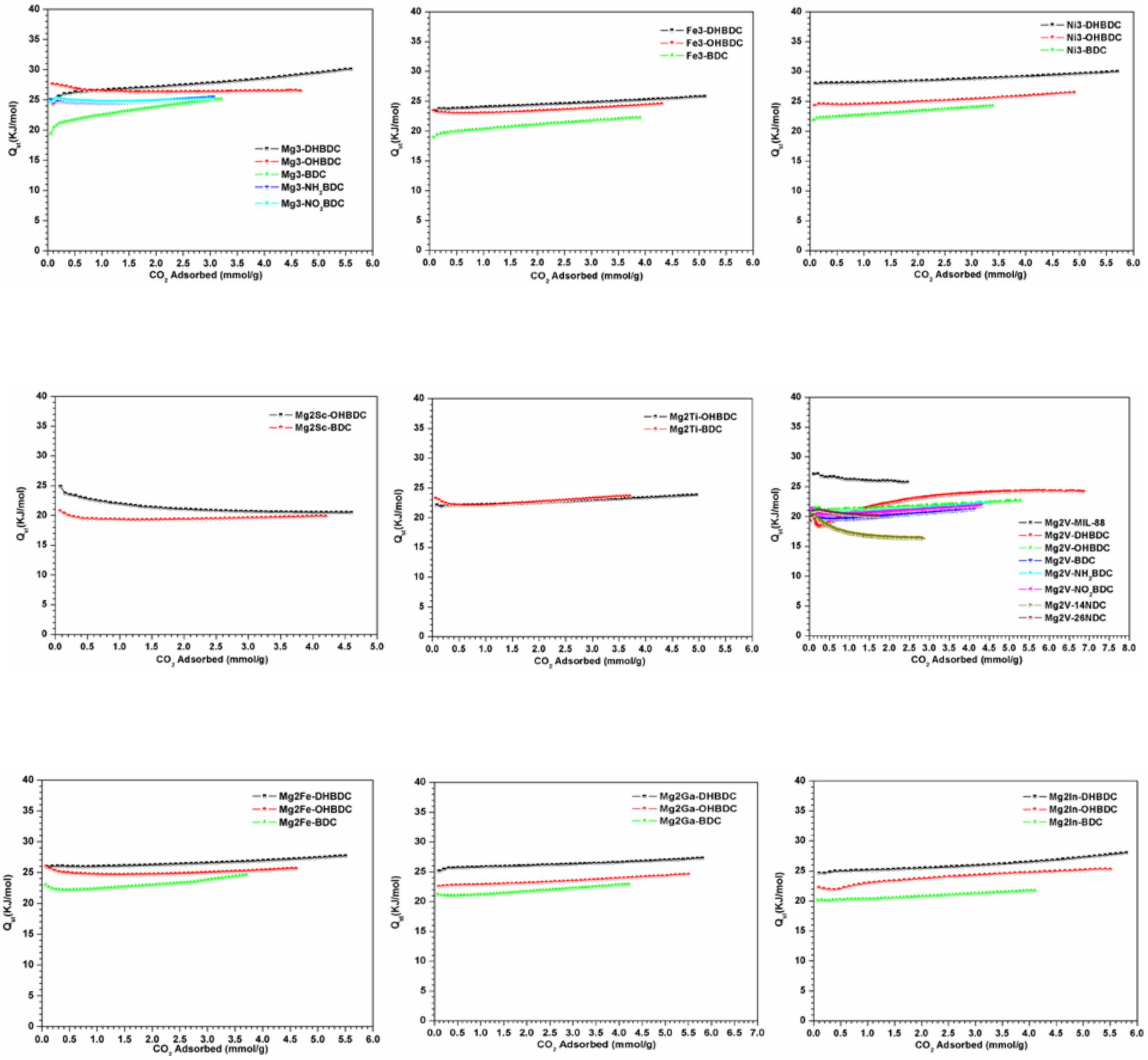

Supplementary Figure 55. Isosteric heat for CO_2 for MOFs reported in this work.

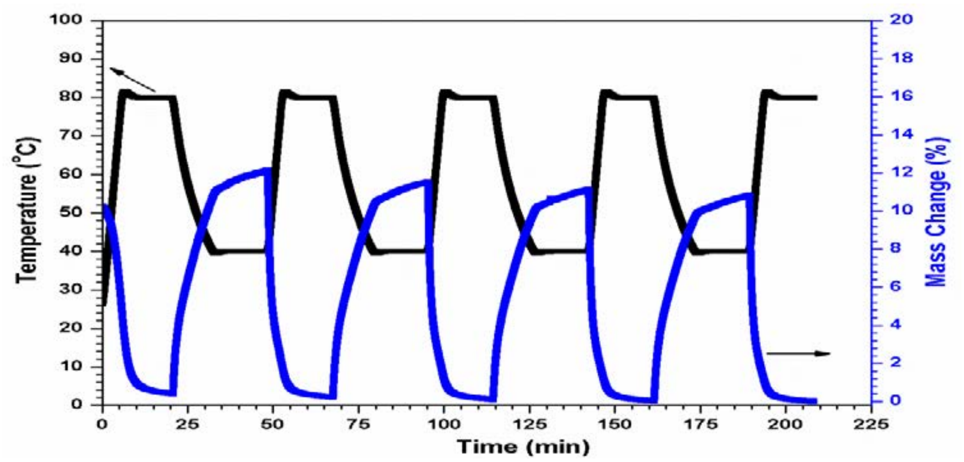

(a)

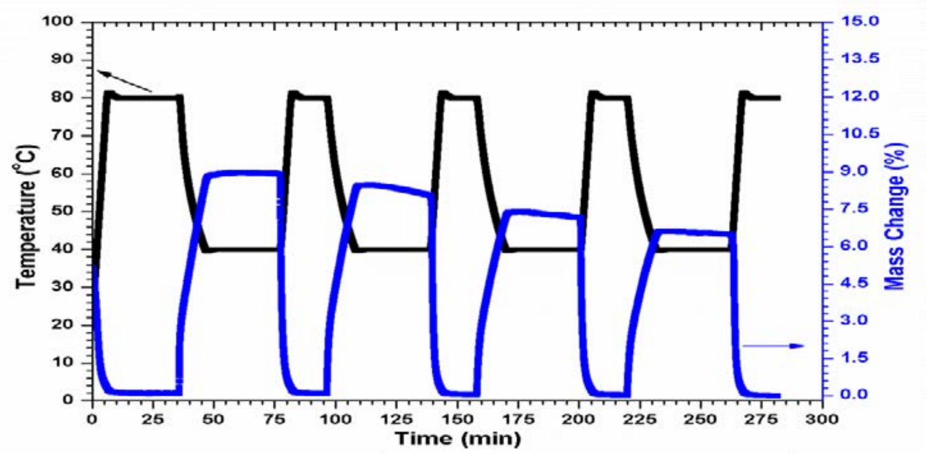

(b)

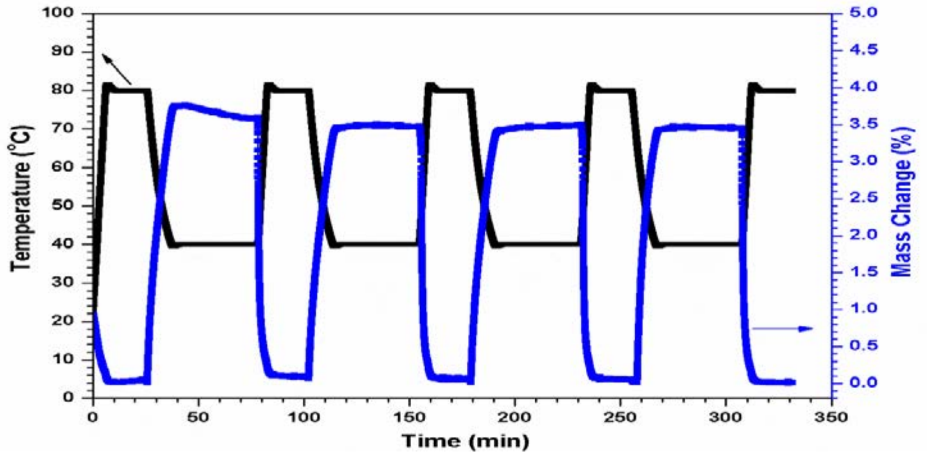

(c)

Supplementary Figure 56. Repeated adsorption–desorption kinetics for **Mg2V-DHBDC (CPM-231)**-(a), **Mg2V-OHBDC (CPM-232)**-(b) and **Mg2V-BDC (CPM-233)**-(c) between a 15 : 85 CO_2/N_2 (v/v) flow at 313 K and a pure N_2 flow at 353 K.

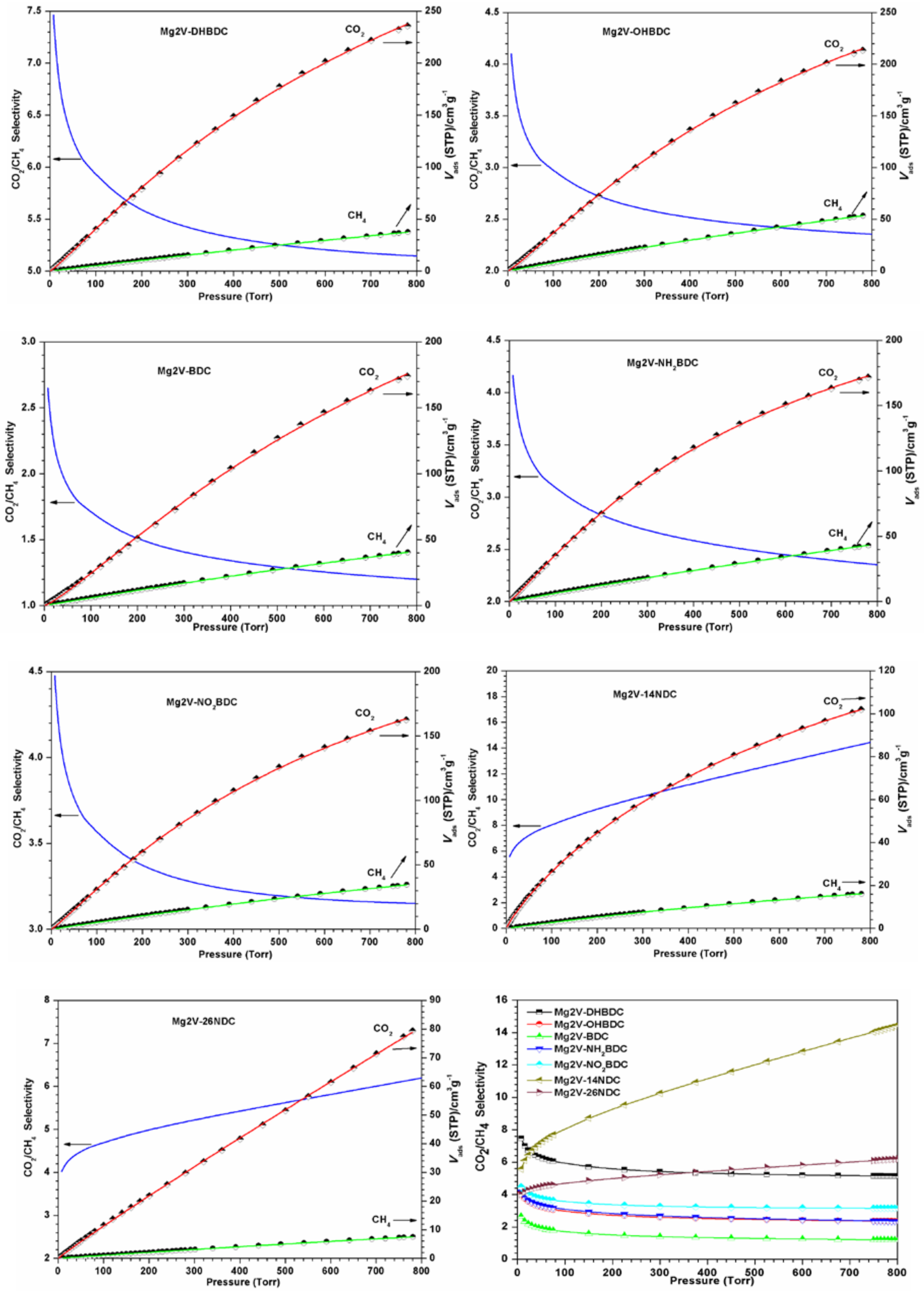

Supplementary Figure 57. Adsorption selectivity predicted by IAST of **[Mg2V(OH)(dicarboxylate)$_3$(TPT)] (CPM-231-CPM-237)** for CO_2 (50%) - CH_4 (50%) at 273 K.

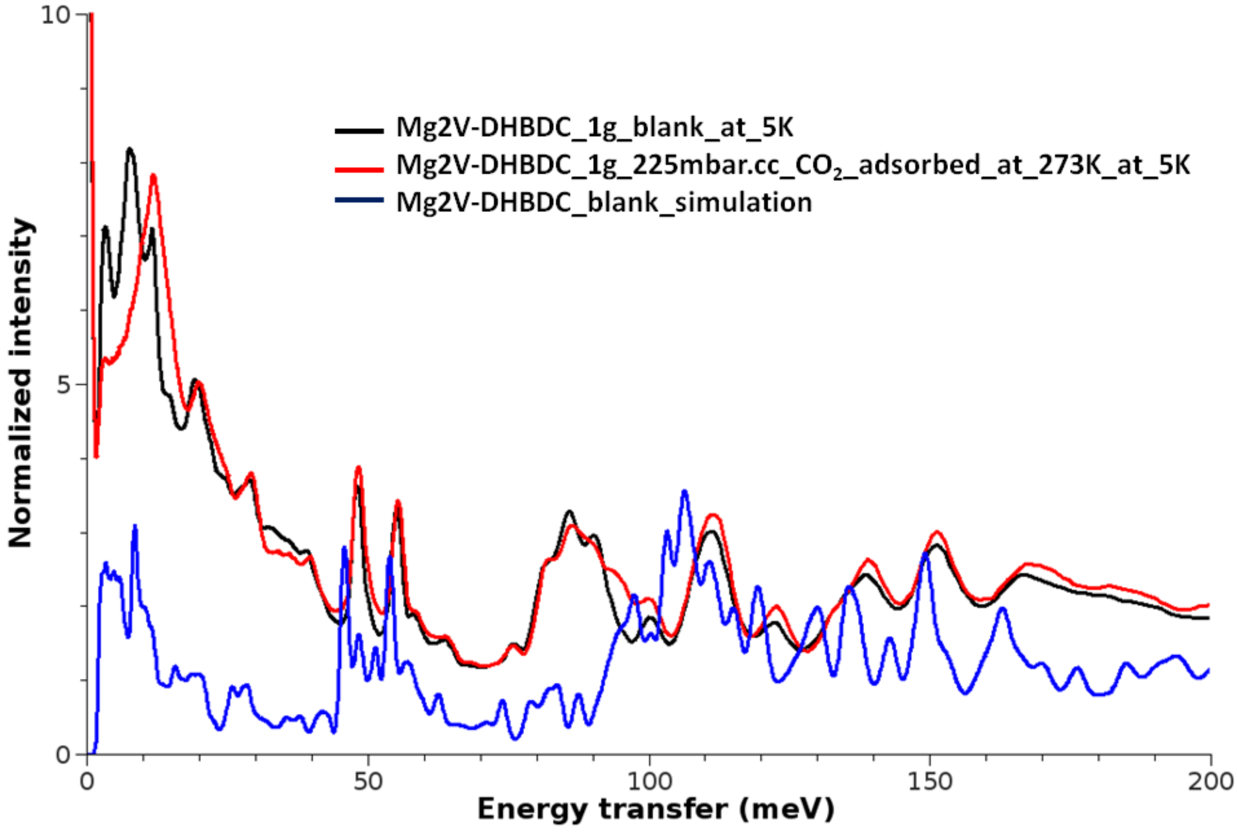

Supplementary Figure 58. Comparison of the experimental and DFT-simulated INS spectra for bare and CO_2-loaded **Mg2V-DHBDC (CPM-231)**.

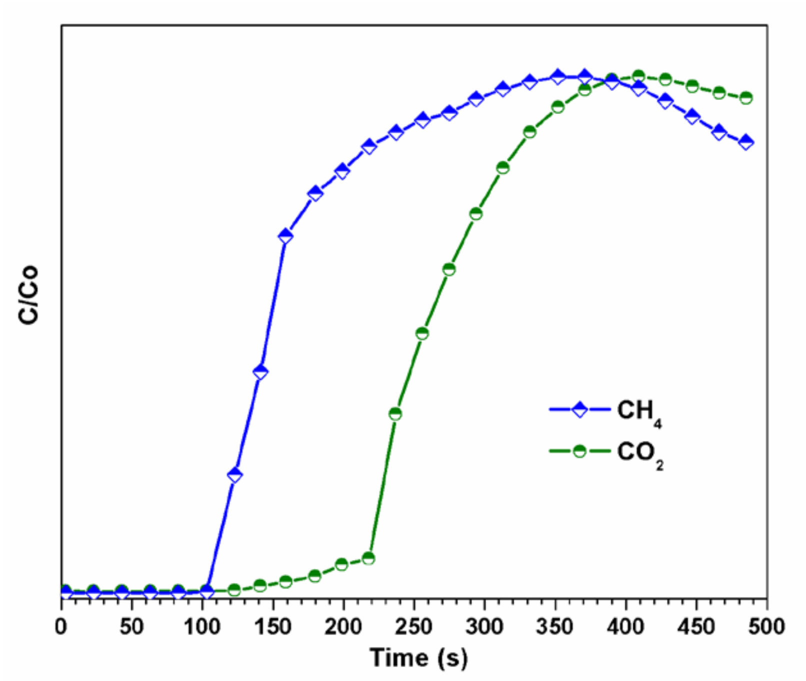

(a)

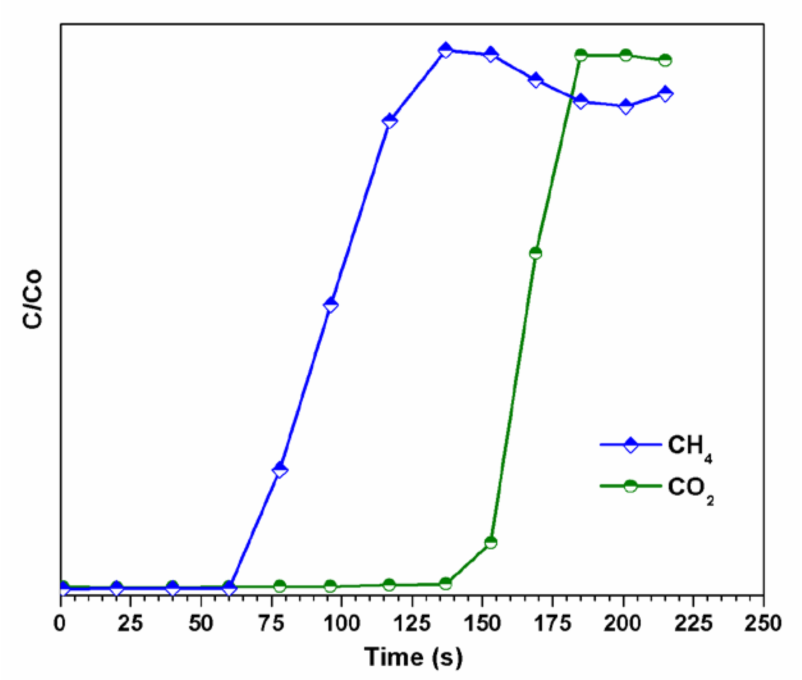

(b)

Supplementary Figure 59. Breakthrough curves for the separation of a 0.15:0.85 (v/v) 16 mL min^{-1} flow of CO_2/CH_4 mixture at 296 K by **Mg2V-DHBDC (CPM-231)** (a) and **Mg2V-OHBDC (CPM-232)** (b).

Supplementary Table 1. The numbering scheme and names for ***pacs*-[M_3(OH/O)(L)$_3$(TPT)] (L = dicarboxylate)** MOFs reported in this work.

M3 \ L	DHBDC	OHBDC	BDC	NH_2BDC	NO_2BDC	14NDC	26NDC
**Mg3**	CPM-141<br>Mg3-DHBDC	CPM-142<br>Mg3-OHBDC	CPM-143<br>Mg3-BDc	CPM-144<br>Mg3-NH_2BDc	CPM-145<br>Mg3-NO_2BDC	CPM-146<br>Mg3-14NDC	X
**Mn3**	X	CPM-152<br>Mn3-OHBDC	CPM-153<br>Mn3-BDC	CPM-154<br>Mn3-NH_2BDC	–	–	–
**Fe3**	CPM-161<br>Fe3-DHBDC	CPM-162<br>Fe3-OHBDC	CPM-163<br>Fe3-BDC	–	–	–	–
**Co3**	CPM-171<br>Co3-DHBDC	CPM-172<br>Co3-OHBDC	CPM-173<br>Co3-BDC	CPM-174<br>Co3-NH_2BDC	–	–	–
**Ni3**	CPM-33b*<br>Ni3-DHBDC	CPM-182<br>Ni3-OHBDC	CPM-33a*<br>Ni3-BDC	CPM-33c*<br>Ni3-NH_2BDC	–	CPM-33d*<br>Ni3-14NDC	CPM-34*<br>Ni3-26NDC
**Zn3**	X	CPM-192<br>Zn3-OHBDC	CPM-193<br>Zn3-BDC	CPM-194<br>Zn3-NH_2BDC	–	–	–
**Mg2Sc**	X	CPM-212<br>Mg2Sc-OHBDC	CPM-213<br>Mg2Sc-BDC	–	–	–	–
**Mg2Ti**	X	CPM-222<br>Mg2Ti-OHBDC	CPM-223<br>Mg2Ti-BDC	–	–	–	–
**Mg2V**	CPM-231<br>Mg2V-DHBDC	CPM-232<br>Mg2V-OHBDC	CPM-233<br>Mg2V-BDC	CPM-234<br>Mg2V-NH_2BDC	CPM-235<br>Mg2V-NO_2BDC	CPM-236<br>Mg2V-14NDC	CPM-237<br>Mg2V-26NDC
**Mg2Fe**	CPM-261<br>Mg2Fe-DHBDC	CPM-262<br>Mg2Fe-OHBDC	CPM-263<br>Mg2Fe-BDC	–	–	–	–
**Mg2Ga**	CPM-271<br>Mg2Ga-DHBDC	CPM-272<br>Mg2Ga-OHBDC	CPM-273<br>Mg2Ga-BDC	–	–	–	–
**Mg2In**	CPM-291<br>Mg2In-DHBDC	CPM-292<br>Mg2In-OHBDC	CPM-293<br>Mg2In-BDC	–	–	–	–

X: failed to synthesize the targeted MOFs; –: not investigated; *: reported in our former work

Supplementary Table 2. Summary of the synthesis conditions for all MOFs in this work.

NO.	M1	M2	L1	L2	Solvent	Additive	T °C
**CPM-140**	$Mg(NO_3)_2$	No	DHBDC	No	DMA/DMPU	HFP	100
**CPM-141 to CPM-146**	$Mg(NO_3)_2$	No	corresponding dicarboxylate ligands	TPT	DMA/DMPU	HFP	130
**CPM-152 CPM-153 CPM-154**	$MnCl_2$	No	OHBDC BDC NH_2BDC	TPT	DMA/DMPU	HFP	130
**CPM-161 CPM-162 CPM-163**	$FeCl_3$	No	DHBDC OHBDC BDC	TPT	DMA/DMPU	HCOOH	130
**CPM-171 to CPM-174**	$CoCl_2$	No	corresponding dicarboxylate ligands	TPT	DMA/DMPU	HFP	130
**CPM-182**	$Ni(NO_3)_2$	No	OHBDC	TPT	DMF/EtOH/H_2O	No	120
**CPM-192 CPM-193 CPM-194**	$ZnCl_2$ + $Zn(NO_3)_2$	No	OHBDC BDC NH_2BDC	TPT	DMA/DMPU	HFP	130
**CPM-212 CPM-213**	$Mg(NO_3)_2$	$Sc(NO_3)_3$	OHBDC BDC	TPT	DMA/DMPU	HCOOH	130
**CPM-222 CPM-223**	$MgCl_2$	$TiCp_2Cl_2$	OHBDC BDC	TPT	DMA/DMPU	HCOOH	130
**CPM-230**	$MgCl_2$	VCl_3	DHBDC	No	DMA/DMPU	HCOOH	100
**CPM-231 to CPM-237**	$MgCl_2$	VCl_3	corresponding dicarboxylate ligands	TPT	DMA/DMPU	HCOOH	130
**CPM-261 CPM-262 CPM-263**	$MgCl_2$	$FeCl_3$	DHBDC OHBDC BDC	TPT	DMA/DMPU	HCOOH	130
**CPM-271 CPM-272 CPM-273**	$MgCl_2$	$Ga(NO_3)_3$	DHBDC OHBDC BDC	TPT	DMA/DMPU	HCOOH	130
**CPM-291**	$MgCl_2$	$InCl_3$	DHBDC	TPT	DMA/DMPU	HCOOH	130
**CPM-292 CPM-293**	$Mg(NO_3)_2$	$In(NO_3)_3$	OHBDC BDC	TPT	DMA/DMPU	HCOOH	130

- For all above MOFs, the additive HFP or HCOOH is necessary. The absence or replacement of the corresponding additive will lead to clear solution, unidentified powder, or impure samples.
- For all above MOFs, the change of metal sources also led to lower yield or impurity, even the failed formation of targeted MOFs.
- Reactions at lower temperatures (< 130 °C) usually lead to decreased yield or impurity.

Supplementary Table 3. Summary of gas sorption properties for **Mg3-MIL-88**, **Mg2V-MIL-88** and **Mg2V** *pacs* MOFs.

**Compound code**	**Mg3-MIL-88**	**Mg2V-MIL-88**	**Mg2V-DHBDC**	**Mg2V-OHBDC**	**Mg2V-BDC**	**Mg2V-NH_2BDC**	**Mg2V-NO_2BDC**	**Mg2V-14NDC**	**Mg2V-26NDC**
S_A Langmuir (m^2/g)	21	212	1597	1564	1843	1349	1439	823	2117
S_A BET (m^2/g)	12	152	1140	1089	1320	963	1028	588	1475
Pore volume (cm^3/g)	0.006	0.058	0.564	0.561	0.653	0.478	0.510	0.291	0.753
CO_2 273 K, 1 bar (mmol/g)	1.59	4.25	10.37	9.40	7.65	7.57	7.17	4.49	3.45
CO_2 298 K, 1 bar (mmol/g)	-	2.46	6.77	5.17	4.04	4.48	4.23	2.79	1.69
Q_{st}^0 (kJ/mol)	-	27.08	20.38	21.43	20.93	21.36	21.35	20.31	19.12
C_2H_2 273 K, 1 bar (mmol/g)	-	5.25	11.39	12.17	11.91	9.32	9.06	6.01	6.53
C_2H_2 298 K, 1 bar (mmol/g)	-	3.48	7.93	8.69	8.65	7.37	6.73	4.54	3.34
CH_4 273 K, 1 bar (mmol/g)	-	0.92	1.64	2.34	2.20	2.35	1.89	1.47	0.91
CH_4 298 K, 1 bar (mmol/g)	-	0.60	1.07	1.42	1.33	1.38	1.18	0.87	0.56
H_2 77 K, 1 bar (mmol/g)	-	8.32	12.90	14.56	13.13	11.44	11.69	7.67	8.93
CO_2-CH_4 selectivity (50-50)	-	-	7.46	4.10	2.65	4.17	4.48	5.58	4.02

Supplementary Table 4. Summary of gas sorption properties for **Mg3** and **Ni3** *pacs* MOFs.

**Compound code**	**Mg3-DHBDC**	**Mg3-OHBDC**	**Mg3-BDC**	**Mg3-NH$_2$BDC**	**Mg3-NO$_2$BDC**	**Mg3-14NDC**	**Ni3-DHBDC**	**Ni3-OHBDC**	**Ni3-BDC**
S_A Langmuir (m^2/g)	1248	1336	1326	776	914	807	1361	1348	1330
S_A BET (m^2/g)	901	965	956	561	660	579	982	973	960
Pore volume (cm^3/g)	0.456	0.498	0.474	0.283	0.326	0.302	0.472	0.481	0.478
CO_2 273 K, 1 bar (mmol/g)	8.69	7.37	6.00	4.46	4.84	2.44	8.35	7.72	6.00
CO_2 298 K, 1 bar (mmol/g)	5.53	4.60	3.19	3.05	3.01	2.09	5.71	4.83	3.34
Q_{st}^0 (kJ/mol)	24.36	27.59	19.33	25.05	24.67	19.04	27.96	24.28	21.81
H_2 77 K, 1 bar (mmol/g)	11.57	10.73	10.30	7.24	7.37	6.10	11.20	11.16	9.79

Supplementary Table 5. Summary of gas sorption properties for **Mn3**, **Co3** and **Zn3** *pacs* MOFs.

**Compound code**	**Mn3-OHBDC**	**Mn3-BDC**	**Mn3-NH_2BDC**	**Co3-DHBDC**	**Co3-OHBDC**	**Co3-BDC**	**Co3-NH_2BDC**	**Zn3-OHBDC**	**Zn3-BDC**	**Zn3-NH_2BDC**
S_A Langmuir (m^2/g)	-	-	-	-	-	-	-	-	-	-
S_A BET (m^2/g)	-	-	-	-	-	-	-	-	-	-
Pore volume (cm^3/g)	-	-	-	-	-	-	-	-	-	-
CO_2 273 K, 1 bar (mmol/g)	5.53	4.78	3.64	8.65	8.24	5.98	4.04	5.31	3.03	2.95
CO_2 298 K, 1 bar (mmol/g)	3.63	-	-	6.02	-	-	-	-	-	-
Q_{st}^0 (kJ/mol)	27.5	-	-	32.8	-	-	-	-	-	-
H_2 77 K, 1 bar (mmol/g)	-	-	-	-	-	-	-	-	-	-

Supplementary Table 6. Summary of gas sorption properties for **Fe3**, **Mg2Sc** and **Mg2Ti** *pacs* MOFs.

**Compound code**	**Fe3-DHBDC**	**Fe3-OHBDC**	**Fe3-BDC**	**Mg2Sc-OHBDC**	**Mg2Sc-BDC**	**Mg2Ti-OHBDC**	**Mg2Ti-BDC**
S_A Langmuir (m^2/g)	1367	1512	1749	1987	1831	1805	1652
S_A BET (m^2/g)	987	1091	1264	1433	1323	1305	1194
Pore volume (cm^3/g)	0.468	0.518	0.624	0.721	0.665	0.650	0.590
CO_2 273 K, 1 bar (mmol/g)	8.04	7.73	7.58	7.76	7.33	9.03	7.63
CO_2 298 K, 1 bar (mmol/g)	5.02	4.28	3.81	4.59	4.11	4.92	3.63
Q_{st}^0 (kJ/mol)	23.36	23.37	18.89	24.83	20.72	22.11	23.21
H_2 77 K, 1 bar (mmol/g)	10.79	11.43	12.75	11.62	12.06	13.64	13.03

Supplementary Table 7. Summary of gas sorption properties for **Mg2Fe**, **Mg2Ga** and **Mg2In** *pacs* MOFs.

**Compound code**	**Mg2Fe-DHBDC**	**Mg2Fe-OHBDC**	**Mg2Fe-BDC**	**Mg2Ga-DHBDC**	**Mg2Ga-OHBDC**	**Mg2Ga-BDC**	**Mg2In-DHBDC**	**Mg2In-OHBDC**	**Mg2In-BDC**
S_A Langmuir (m^2/g)	1400	1535	1554	1252	1714	1719	1107	1785	1592
S_A BET (m^2/g)	1012	1109	1123	903	1240	1243	799	1289	1150
Pore volume (cm^3/g)	0.508	0.554	0.557	0.395	0.621	0.614	0.392	0.640	0.573
CO_2 273 K, 1 bar (mmol/g)	8.56	8.22	7.84	9.44	8.90	7.91	8.99	8.74	7.78
CO_2 298 K, 1 bar (mmol/g)	5.45	4.53	3.64	6.11	5.44	4.11	5.79	5.39	4.06
Q_{st}^0 (kJ/mol)	25.92	26.00	22.95	25.13	22.52	21.14	24.63	22.21	20.07
H_2 77 K, 1 bar (mmol/g)	11.59	11.28	11.31	12.04	13.58	12.92	10.07	12.16	11.25

Supplementary Table 8. Summary of MOFs with highest CO_2 uptake at 1 bar.

Compound code	CO_2 uptake (mmol/g)		Type of	Reference
	273 K	298 K	active sites	
Mg2V-DHBDC (CPM-231)	10.37	6.77	PSP	this work
Mg-MOF-74	10.22	8.04	OMS	Refs 26-27 in main article
Co-MOF-74	NA	6.96	OMS	Ref. 26 in main article
Cu-TDPAT	10.13	7.94	OMS+LBS	Ref. 6 in main article
MAF-X25ox	NA	7.14	OMS+LBS	1
Cu-TPBTM	9.68	5.29	OMS+LBS	2
Mg2Ga-DHBDC (CPM-271)	9.44	6.11	PSP	this work
Mg2V-OHBDC (CPM-232)	9.40	5.17	PSP	this work
JLU-Liu21	9.38	5.27	OMS+LBS	3
CPM-200-Fe/Mg	9.27	5.68	OMS	Ref. 31 in main article
NJU-Bai21	9.22	5.14	OMS+LBS	4
[Cu(Me-4py-trz-ia)]	9.20	6.10	OMS+LBS	5
NOTT-125	9.09	4.13	OMS+LBS	6
Mg2Ti-OHBDC (CPM-222)	9.03	4.92	PSP	this work
NbO-Pd-1	9.00	5.60	OMS	7
Mg2In-DHBDC (CPM-291)	8.99	5.79	PSP	this work
Mg2Ga-OHBDC (CPM-272)	8.90	5.44	PSP	this work
LCu'	8.86	4.38	OMS+LBS	8
SNU-5	8.75	-	OMS	9
Mg2In-OHBDC (CPM-292)	8.74	5.39	PSP	this work
Mg3-DHBDC (CPM-140)	8.69	5.53	PSP	this work
Co3-DHBDC (CPM-171)	8.65	6.02	PSP	this work
Mg2Fe-DHBDC (CPM-261)	8.56	5.45	PSP	this work
Co3-OHBDC (CPM-172)	8.24	-	PSP	this work
Mg2Fe-OHBDC (CPM-262)	8.22	4.53	PSP	this work
Fe3-DHBDC (CPM-161)	8.04	5.02	PSP	this work
Mg2Ga-BDC (CPM-273)	7.91	4.11	PSP	this work
Mg2Fe-BDC (CPM-263)	7.84	3.64	PSP	this work
Mg2In-BDC (CPM-293)	7.78	4.06	PSP	this work
Mg2Sc-OHBDC (CPM-212)	7.76	4.59	PSP	this work
Fe3-OHBDC (CPM-162)	7.73	4.28	PSP	this work
Ni3-OHBDC (CPM-182)	7.72	4.83	PSP	this work
Mg2V-BDC (CPM-233)	7.65	4.04	PSP	this work
Mg2Ti-BDC (CPM-223)	7.63	4.04	PSP	this work
Fe3-BDC (CPM-163)	7.58	3.81	PSP	this work
Mg2V-NH_2BDC (CPM-234)	7.57	4.48	PSP	this work
Mg3-OHBDC (CPM-143)	7.37	4.60	PSP	this work
Mg2Sc-BDC (CPM-213)	7.33	4.11	PSP	this work
Mg2V-NO_2BDC (CPM-235)	7.17	4.23	PSP	this work
PCN-88	7.14	5.50	OMS	10
rht-MOF-7	6.52	4.76	OMS+LBS	11
SIFSIX-2-Cu-i	6.50	5.41	LBS	12
MAF-66	6.26	4.41	LBS	13
bio-MOF-11	6.00	4.10	LBS	14

NA = not available; PSP = pore space partition; OMS = open metal site; LBS = Lewis basic site

Supplementary Table 9. Crystal data and structure refinements for **Mg3-MIL-88 (CPM-140)** and **Mg3-DHBDC (CPM-141)**.

	**Mg3-MIL-88 (CPM-140)**	**Mg3-DHBDC (CPM-141)**
Empirical formula	$Mg_3C_{26}H_{27}NO_{22}$	$Mg_3C_{44}H_{33}N_7O_{19}$
Formula weight	778.42	1036.70
Temperature (K)	200(2)	200(2)
Crystal system, Space group	Hexagonal, *P*6(3)/*mmc*	Hexagonal, *P*6(3)/*mmc*
Unit cell dimensions	$a = b$ = 16.2122(3)<br>c = 16.0642(7)Å<br>V = 3656.57(19)Å^3	$a = b$ = 16.9671(5)<br>c = 15.3079(10) Å<br>V = 3816.5(3) Å^3
Z, Density(cal.)	2, 0.707 g/cm^3	2, 0.902 g/cm^3
Absorption coefficient	0.085 mm^{-1}	0.093 mm^{-1}
F(000)	804	1068
Theta range for data collection	1.45^o to 25.03^o	1.92^o to 25.10^o
Limiting indices	-18<=h<=18, -18<=k<=16, -19<=l<=18	-20<=h<=10, -15<=k<=19, -17<=l<=18
Reflections collected / unique	13456/1247 [R(int) = 0.0762]	13059 / 1318 [R(int) = 0.0705]
Data Completeness measured	99.3 %	99.8 %
Refinement Method	Full-matrix least-squares on F^2	Full-matrix least-squares on F^2
Parameter/Restraints/Data(obs.)	1247 / 0 / 50	1318 / 0 / 74
Goodness-of-fit	1.067	1.033
Final R indices ($I > 2\sigma(I)$)	R1 = 0.0571, wR2 = 0.1693	R1 = 0.0431, wR2 = 0.1076
R indices (all)	R1 = 0.0750, wR2 = 0.1767	R1 = 0.0581, wR2 = 0.1126
Largest difference peaks	1.104 and -0.253 e·A^{-3}	0.250 and -0.367 e·A^{-3}

[a] $R1 = \sum(|F_o| - |F_c|) / \sum|F_o|$, $wR2 = [\sum w(F_o^2 - F_c^2)^2 / \sum w(F_o^2)^2]^{0.5}$.

Supplementary Table 10. Crystal data and structure refinements for **Mg3-OHBDC (CPM-142)** and **Mg3-BDC (CPM-143)**.

	**Mg3-OHBDC (CPM-142)**	**Mg3-BDC (CPM-143)**
Empirical formula	$Mg_3C_{44}H_{33}N_7O_{16}$	$Mg_3C_{44}H_{33}N_7O_{13}$
Formula weight	988.70	940.70
Temperature (K)	200(2)	200(2)
Crystal system, Space group	Hexagonal, *P*6(3)/*mmc*	Hexagonal, *P*6(3)/*mmc*
Unit cell dimensions	*a* = *b* = 16.9886(16)<br>*c* = 15.108(3) Å<br>*V* = 3776.2(10) Å^3	*a* = *b* = 17.0231(11)<br>*c* = 14.947(3) Å<br>*V* = 3751.1(9) Å^3
Z, Density(cal.)	2, 0.870 g/cm^3	2, 0.833 g/cm^3
Absorption coefficient	0.089 mm^{-1}	0.084 mm^{-1}
F(000)	1020	972
Theta range for data collection	1.93^o to 25.03^o	1.94^o to 25.04^o
Limiting indices	-10<=h<=20, -20<=k<=13, -17<=l<=17	-14<=h<=14, -19<=k<=19, -12<=l<=17
Reflections collected / unique	11676 / 1289 [R(int) = 0.0775]	11630 / 1284 [R(int) = 0.0939]
Data Completeness measured	99.8 %	99.9%
Refinement Method	Full-matrix least-squares on F^2	Full-matrix least-squares on F^2
Parameter/Restraints/Data(obs.)	1289 / 0 / 74	1284 / 0 / 65
Goodness-of-fit	1.053	1.005
Final R indices (*I* >2*σ*(*I*))	R1 = 0.0512, wR2 = 0.1381	R1 = 0.0487, wR2 = 0.1235
R indices (all)	R1 = 0.0722, wR2 = 0.1462	R1 = 0.0750, wR2 = 0.1320
Largest difference peaks	0.430 and -0.403 e·A^{-3}	0.471 and -0.335 e·A^{-3}

[a] $R1 = \sum(|F_o| - |F_c|) / \sum|F_o|$, $wR2 = [\sum w(F_o^2 - F_c^2)^2 / \sum w(F_o^2)^2]^{0.5}$.

Supplementary Table 11. Crystal data and structure refinements for **Mg3-NH_2BDC (CPM-144)** and **Mn3-BDC (CPM-153)**.

	**Mg3-NH_2BDC (CPM-144)**	**Mn3-BDC (CPM-153)**
Empirical formula	$Mg_3C_{44}H_{36}N_{10}O_{13}$	$Mn_3C_{42}H_{25}N_6O_{13}$
Formula weight	985.76	986.50
Temperature (K)	200(2)	200(2)
Crystal system, Space group	Hexagonal, *P*6(3)/*mmc*	Hexagonal, *P*6(3)/*mmc*
Unit cell dimensions	*a* = *b* = 16.9970(4)<br>*c* = 14.9864(9) Å<br>*V* = 3749.5(3) $Å^3$	*a* = *b* = 17.4162(4)<br>*c* = 14.9154(7) Å<br>3918.1(3) $Å^3$
Z, Density(cal.)	2, 0.873 g/cm^3	2, 0.836 g/cm^3
Absorption coefficient	0.088 mm^{-1}	0.515 mm^{-1}
F(000)	1020	996
Theta range for data collection	1.94° to 25.05°	1.94° to 25.35°
Limiting indices	-15<=h<=19, -17<=k<=17, -17<=l<=14	-14<=h<=16, -20<=k<=20, -17<=l<=7
Reflections collected / unique	11661 / 1282 [R(int) = 0.0448]	12521 / 1382 [R(int) = 0.0479]
Data Completeness measured	99.9 %	99.9%
Refinement Method	Full-matrix least-squares on F^2	Full-matrix least-squares on F^2
Parameter/Restraints/Data(obs.)	1282 / 0 / 73	1336 / 0 / 65
Goodness-of-fit	1.091	1.027
Final R indices ($I > 2\sigma(I)$)	R1 = 0.0670, wR2 = 0.2027	R1 = 0.0345, wR2 = 0.1005
R indices (all)	R1 = 0.0754, wR2 = 0.2102	R1 = 0.0423, wR2 = 0.1034
Largest difference peaks	0.658 and -0.358 e·A^{-3}	0.471 and -0.357 e·A^{-3}

[a] $R1 = \sum(|F_o| - |F_c|) / \sum|F_o|$, $wR2 = [\sum w(F_o^2 - F_c^2)^2 / \sum w(F_o^2)^2]^{0.5}$.

Supplementary Table 12. Crystal data and structure refinements for **Co3-NH_2BDC (CPM-174)** and **Zn3-NH_2BDC (CPM-194)**.

	**Co3-NH_2BDC (CPM-174)**	**Zn3-NH_2BDC (CPM-194)**
Empirical formula	$Co_3C_{42}H_{28}N_9O_{13}$	$Zn_3C_{42}H_{28}N_9O_{13}$
Formula weight	1043.52	1062.84
Temperature (K)	200(2)	200(2)
Crystal system, Space group	Hexagonal, *P*6(3)/*mmc*	Hexagonal, *P*6(3)/*mmc*
Unit cell dimensions	*a* = *b* = 17.0539(4)<br>*c* = 14.9837(7) Å<br>*V* = 3774.0(2) $Å^3$	*a* = *b* = 17.0448(4)<br>*c* = 14.9913(7) Å<br>*V* = 3771.8(2) $Å^3$
Z, Density(cal.)	2, 0.918 g/cm^3	2, 0.936 g/cm^3
Absorption coefficient	0.696 mm^{-1}	0.990 mm^{-1}
F(000)	1056	1074
Theta range for data collection	1.38^o to 25.03^o	1.38^o to 20.90^o
Limiting indices	-18<=h<=20, -20<=k<=20, -16<=l<=17	-16<=h<=16, -16<=k<=16, -7<=l<=15
Reflections collected / unique	19028 / 1292 [R(int) = 0.0509]	8031 / 795 [R(int) = 0.0735]
Data Completeness measured	100.0 %	100.0 %
Refinement Method	Full-matrix least-squares on F^2	Full-matrix least-squares on F^2
Parameter/Restraints/Data(obs.)	1292 / 0 / 73	795 / 0 / 73
Goodness-of-fit	1.004	0.980
Final R indices ($I > 2\sigma(I)$)	R1 = 0.0500, wR2 = 0.1668	R1 = 0.0501, wR2 = 0.1545
R indices (all)	R1 = 0.0572, wR2 = 0.1719	R1 = 0.0585, wR2 = 0.1611
Largest difference peaks	0.609 and -0.334 e·A^{-3}	0.610 and -0.395 e·A^{-3}

[a] $R1 = \sum(|F_o| - |F_c|) / \sum|F_o|$, $wR2 = [\sum w(F_o^2 - F_c^2)^2 / \sum w(F_o^2)^2]^{0.5}$.

Supplementary Table 13. Crystal data and structure refinements for **Mg2V-MIL-88 (CPM-230)** and **Mg2V-DHBDC (CPM-231)**.

	**Mg2V-MIL-88 (CPM-230)**	**Mg2V-DHBDC (CPM-231)**
Empirical formula	$Mg_2VC_{24}H_{19}O_{22}$	$Mg_2VC_{42}H_{25}N_6O_{19}$
Formula weight	758.95	1017.24
Temperature (K)	200(2)	200(2)
Crystal system, Space group	Hexagonal, *P*6(3)/*mmc*	Hexagonal, *P*6(3)/*mmc*
Unit cell dimensions	$a = b$ = 15.9423(3)<br>c = 16.0411(7) Å<br>V = 3530.75 $Å^3$	$a = b$ = 16.6165(10)<br>c = 15.3366(19) Å<br>V = 3667.2(6) $Å^3$
Z, Density(cal.)	2, 0.714 g/cm^3	2, 0.921 g/cm^3
Absorption coefficient	0.200 mm^{-1}	0.205 mm^{-1}
F(000)	772	1036
Theta range for data collection	1.48° to 24.18°	1.42° to 25.03°
Limiting indices	-16<=h<=14, -17<=k<=18, -18<=l<=11	-18<=h<=18, -14<=k<=17, -12<=l<=18
Reflections collected / unique	11884/1103 [R(int) = 0.0534]	10640 / 1252 [R(int) = 0.0524]
Data Completeness measured	99.7 %	99.4 %
Refinement Method	Full-matrix least-squares on F^2	Full-matrix least-squares on F^2
Parameter/Restraints/Data(obs.)	1103 / 0 / 50	1252 / 125 / 74
Goodness-of-fit	1.072	1.358
Final R indices ($I > 2\sigma(I)$)	R1 = 0.0616, wR2 = 0.1823	R1 = 0.1016, wR2 = 0.3266
R indices (all)	R1 = 0.0743, wR2 = 0.1951	R1 = 0.1229, wR2 = 0.3425
Largest difference peaks	0.749 and -0.607 $e·A^{-3}$	0.699 and -0.842 $e·A^{-3}$

[a] $R1 = \sum(|F_o| - |F_c|) / \sum|F_o|$, $wR2 = [\sum w(F_o^2 – F_c^2)^2 / \sum w(F_o^2)^2]^{0.5}$.

Supplementary Table 14. Crystal data and structure refinements for **Mg2V-NO_2BDC (CPM-235) and Mg2V-14NDC (CPM-236)**.

	**Mg2V-NO_2BDC (CPM-235)**	**Mg2V-14NDC (CPM-236)**
Empirical formula	$Mg_2VC_{42}H_{22}N_9O_{19}$	$Mg_2VC_{54}H_{31}N_6O_{13}$
Formula weight	1056.25	1071.41
Temperature (K)	200(2)	200(2)
Crystal system, Space group	Hexagonal, *P*6(3)/*mmc*	Hexagonal, *P*6(3)/*mmc*
Unit cell dimensions	$a = b$ = 16.9065(5)<br>c = 14.7154(10) Å<br>V = 3642.6(3) Å^3	$a = b$ = 16.8599(12)<br>c = 14.882(3) Å<br>V = 3663.5(8) Å^3
Z, Density(cal.)	2, 0.963 g/cm^3	2, 0.971 g/cm^3
Absorption coefficient	0.210 mm^{-1}	0.203 mm^{-1}
F(000)	1072	1096
Theta range for data collection	1.96^o to 24.4^o	1.95^o to 19.15^o
Limiting indices	-16<=h<=16, -19<=k<=16, -14<=l<=17	-12<=h<=12, -15<=k<=13, -13<=l<=12
Reflections collected / unique	10950 / 1164 [R(int) = 0.0876]	6304 / 612 [R(int) = 0.0753]
Data Completeness measured	99.9 %	99.8 %
Refinement Method	Full-matrix least-squares on F^2	Full-matrix least-squares on F^2
Parameter/Restraints/Data(obs.)	1164 / 23 / 77	612 / 72 / 92
Goodness-of-fit	1.030	1.019
Final R indices ($I > 2\sigma(I)$)	R1 = 0.0942, wR2 = 0.2657	R1 = 0.1244, wR2 = 0.3459
R indices (all)	R1 = 0.1145, wR2 = 0.2816	R1 = 0.1364, wR2 = 0.3663
Largest difference peaks	0.895 and -0.448 e·A^{-3}	0.523 and -0.428 e·A^{-3}

[a] $R1 = \sum(|F_o| - |F_c|) / \sum|F_o|$, $wR2 = [\sum w(F_o^2 – F_c^2)^2 / \sum w(F_o^2)^2]^{0.5}$.

Supplementary Table 15. Crystal data and structure refinements for **Mg2V-26NDC (CPM-237)**.

	**Mg2V-26NDC (CPM-237)**
Empirical formula	$Mg_2VC_{54}H_{31}N_6O_{13}$
Formula weight	1071.41
Temperature (K)	200(2)
Crystal system, Space group	Hexagonal, *P*6(3)/*mmc*
Unit cell dimensions	*a* = *b* = 16.7913(2)<br>*c* = 20.6558(5) Å<br>*V* = 5043.61(15) $Å^3$
Z, Density(cal.)	2, 0.705 g/cm^3
Absorption coefficient	0.147 mm^{-1}
F(000)	1096
Theta range for data collection	1.71^o to 25.01^o
Limiting indices	-19<=h<=19, -19<=k<=16, -23<=l<=24
Reflections collected / unique	21146 / 1699 [R(int) = 0.0799]
Data Completeness measured	99.7 %
Refinement Method	Full-matrix least-squares on F^2
Parameter/Restraints/Data(obs.)	1699 / 0 / 101
Goodness-of-fit	1.018
Final R indices ($I > 2\sigma(I)$)	R1 = 0.0807, wR2 = 0.2260
R indices (all)	R1 = 0.1207, wR2 = 0.2473
Largest difference peaks	0.435 and -0.348 e·A^{-3}

[a] $R1 = \sum(|F_o| - |F_c|) / \sum|F_o|$, $wR2 = [\sum w(F_o^2 - F_c^2)^2 / \sum w(F_o^2)^2]^{0.5}$.

Supplementary Methods

Materials. All chemicals were purchased from Aldrich Chemical Co. and used as received without further purification.

Metal source. $Mg(NO_3)_2 \cdot 6H_2O$, $MgCl_2 \cdot 6H_2O$, $Sc(NO_3)_3 \cdot xH_2O$, bis(cyclopentadienyl)titanium(IV) dichloride ($TiCp_2Cl_2$), VCl_3, $MnCl_2$, $FeCl_3 \cdot 6H_2O$, $CoCl_2 \cdot 6H_2O$, $Ni(NO_3)_2 \cdot 6H_2O$, $ZnCl_2$, $Zn(NO_3)_2 \cdot 6H_2O$, $Ga(NO_3)_3 \cdot xH_2O$, $InCl_3$, and $In(NO_3)_3 \cdot xH_2O$.

Organic ligand. 2,5-dihydroxybenzene-1,4-dicarboxylic acid (DHBDC), 2-hydroxybenzene-1,4-dicarboxylic acid (OHBDC), 1,4-terephthalic acid (BDC), 2-aminobenzene-1,4-dicarboxylic acid (NH_2BDC), 2-nitrobenzene-1,4-dicarboxylic acid (NO_2BDC), naphthalene-1,4-dicarboxylic acid (14NDC), naphthalene-2,6-dicarboxylic acid (26NDC), and 2,4,6-tri(4-pyridinyl)-1,3,5-triazine (TPT).

Solvent and additive. N,N-dimethylacetamide (DMA), N,N-dimethylformamide (DMF), 1,3-dimethyl-3,4,5,6-tetrahydro-2(1H)-pyrimidinone (DMPU), 1,1,1,5,5,5-hexafluoro-2,4-pentanedione (HFP), methanol (CH_3OH), ethanol (CH_3CH_2OH) and 85% formic acid.

Synthesis of $[(CH_3)_2NH_2][Mg_3(OH)(DHBDC)_3(H_2O)_3]$ (CPM-140, Mg3-MIL-88). In a 20 ml glass vial, 102.6 mg of $Mg(NO_3)_2 \cdot 6H_2O$ and 39.6 mg of DHBDC were dissolved in a mixture of 4.0 g of DMA and 2.0 g of DMPU. After addition of 28 µL HFP, the vial was sealed and placed in a 100 ^{o}C oven for 5 days. Large yellow crystals were obtained after cooling to room temperature. Pure sample was obtained by filtering and washing the raw product with DMA. The yield was about 86% based on DHBDC.

Synthesis of $[(CH_3)_2NH_2][Mg_3(OH)(DHBDC)_3(TPT)]$ (CPM-141, Mg3-DHBDC). In a 20 ml glass vial, 102.6 mg of $Mg(NO_3)_2 \cdot 6H_2O$, 39.6 mg of DHBDC, 31.2 mg of TPT were dissolved in a mixture of 4.0 g of DMA and 2.0 g of DMPU. After addition of 28 µL HFP, the vial was sealed and placed in a 130 ^{o}C oven for 5 days. Large dark red crystals were obtained after cooling to room temperature. Pure sample was obtained by filtering and washing the raw product with hot DMA/DMPU mixtures. The yield was about 34% based on DHBDC.

Synthesis of $[(CH_3)_2NH_2][Mg_3(OH)(OHBDC)_3(TPT)]$ (CPM-142, Mg3-OHBDC). In a 20 ml glass vial, 102.6 mg of $Mg(NO_3)_2 \cdot 6H_2O$, 36.4 mg of OHBDC, 31.2 mg of TPT were dissolved in a mixture of 4.0 g of DMA and 2.0 g of DMPU. After addition of 28 µL HFP, the vial was sealed and placed in a 130 ^{o}C oven for 5 days. Large orange crystals were obtained after cooling to

room temperature. Pure sample was obtained by filtering and washing the raw product with DMA. The yield was about 65% based on OHBDC.

Synthesis of [$(CH_3)_2NH_2$][$Mg_3(OH)(BDC)_3(TPT)$] (CPM-143, Mg3-BDC). In a 20 ml glass vial, 102.6 mg of $Mg(NO_3)_2 \cdot 6H_2O$, 33.2 mg of BDC, 31.2 mg of TPT were dissolved in a mixture of 4.0 g of DMA and 2.0 g of DMPU. After addition of 28 µL HFP, the vial was sealed and placed in a 130 ^oC oven for 5 days. Large orange crystals were obtained after cooling to room temperature. Pure sample was obtained by filtering and washing the raw product with DMA. The yield was about 60% based on BDC.

Synthesis of [$(CH_3)_2NH_2$][$Mg_3(OH)(NH_2BDC)_3(TPT)$] (CPM-144, Mg3-NH_2BDC). In a 20 ml glass vial, 102.6 mg of $Mg(NO_3)_2 \cdot 6H_2O$, 36.2 mg of NH_2BDC, 31.2 mg of TPT were dissolved in a mixture of 4.0 g of DMA and 2.0 g of DMPU. After addition of 28 µL HFP, the vial was sealed and placed in a 130 ^oC oven for 5 days. Large red crystals were obtained after cooling to room temperature. Pure sample was obtained by filtering and washing the raw product with DMA. The yield was about 75% based on NH_2BDC.

Synthesis of [$(CH_3)_2NH_2$][$Mg_3(OH)(NO_2BDC)_3(TPT)$] (CPM-145, Mg3-NO_2BDC). In a 20 ml glass vial, 102.6 mg of $Mg(NO_3)_2 \cdot 6H_2O$, 42.2 mg of NO_2BDC, 31.2 mg of TPT were dissolved in a mixture of 4.0 g of DMA and 2.0 g of DMPU. After addition of 28 µL HFP, the vial was sealed and placed in a 130 ^oC oven for 5 days. Orange needle micro-crystals were obtained after cooling to room temperature. Pure sample was obtained by filtering and washing the raw product with DMA. The yield was about 45% based on NO_2BDC.

Synthesis of [$(CH_3)_2NH_2$][$Mg_3(OH)(14NDC)_3(TPT)$] (CPM-146, Mg3-14NDC). In a 20 ml glass vial, 102.6 mg of $Mg(NO_3)_2 \cdot 6H_2O$, 43.2 mg of 14NDC, 31.2 mg of TPT were dissolved in a mixture of 4.0 g of DMA and 2.0 g of DMPU. After addition of 28 µL HFP, the vial was sealed and placed in a 130 ^oC oven for 5 days. Orange needle micro-crystals were obtained after cooling to room temperature. Pure sample was obtained by filtering and washing the raw product with DMA. The yield was about 42% based on NO_2BDC.

Synthesis of [$Mn_3(OH)(OHBDC)_3(TPT)$] (CPM-152, Mn3-OHBDC). In a 20 ml glass vial, 50.4 mg of $MnCl_2$, 36.4 mg of DHBDC and 62.4 mg of TPT were dissolved in a mixture of 4.0 g of DMA and 2.0 g of DMPU. After addition of 28 µL HFP, the vial was sealed and placed in a 130 ^oC oven for 3 days. Yellow polyhedral crystals were obtained after cooling to room temperature. Pure sample was obtained by filtering and washing the raw product with hot DMA/DMPU mixtures for 2 times. The yield was about 46% based on OHBDC.

Synthesis of [Mn_3(OH)($BDC)_3$(TPT)] (CPM-153, Mn3-BDC). In a 20 ml glass vial, 50.4 mg of $MnCl_2$, 33.2 mg of BDC and 62.4 mg of TPT were dissolved in a mixture of 4.0 g of DMA and 2.0 g of DMPU. After addition of 28 µL HFP, the vial was sealed and placed in a 130 ^oC oven for 3 days. Large yellow polyhedral crystals were obtained after cooling to room temperature. Pure sample was obtained by filtering and washing the raw product with hot DMA/DMPU mixtures for 2 times. The yield was about 50% based on BDC.

Synthesis of [Mn_3(OH)($NH_2BDC)_3$(TPT)] (CPM-154, Mn3-NH_2BDC). In a 20 ml glass vial, 50.4 mg of $MnCl_2$, 36.2 mg of NH_2BDC and 62.4 mg of TPT were dissolved in a mixture of 4.0 g of DMA and 2.0 g of DMPU. After addition of 28 µL HFP, the vial was sealed and placed in a 130 ^oC oven for 3 days. Yellow polyhedral crystals were obtained after cooling to room temperature. Pure sample was obtained by filtering and washing the raw product with hot DMA/DMPU mixtures for 2 times. The yield was about 53% based on NH_2BDC.

Synthesis of [Fe_3(O)($DHBDC)_3$(TPT)] (CPM-161, Fe3-DHBDC). In a 20 ml glass vial, 135.2 mg of $FeCl_3{\cdot}6H_2O$, 99.0 mg of DHBDC, 62.4 mg of TPT were dissolved in a mixture of 4.0 g of DMA and 2.0 g of DMPU. After addition of 0.5 g 85% formic acid, the vial was sealed and placed in a 130 ^oC oven for 5 days. Dark brown micro-crystals were obtained after cooling to room temperature. Pure sample was obtained by filtering and washing the raw product with hot DMA/DMPU mixtures for 3-5 times. The yield was about 48% based on Fe.

Synthesis of [Fe_3(O)($OHBDC)_3$(TPT)] (CPM-162, Fe3-OHBDC). In a 20 ml glass vial, 135.2 mg of $FeCl_3{\cdot}6H_2O$, 91.0 mg of OHBDC, 62.4 mg of TPT were dissolved in a mixture of 4.0 g of DMA and 2.0 g of DMPU. After addition of 0.5 g 85% formic acid, the vial was sealed and placed in a 130 ^oC oven for 5 days. Dark brown micro-crystals were obtained after cooling to room temperature. Pure sample was obtained by filtering and washing the raw product with hot DMA/DMPU mixtures for 3-5 times. The yield was about 58% based on Fe.

Synthesis of [Fe_3(O)($BDC)_3$(TPT)] (CPM-163, Fe3-BDC). In a 20 ml glass vial, 135.2 mg of $FeCl_3{\cdot}6H_2O$, 83.0 mg of BDC, 62.4 mg of TPT were dissolved in a mixture of 4.0 g of DMA and 2.0 g of DMPU. After addition of 0.5 g 85% formic acid, the vial was sealed and placed in a 130 ^oC oven for 5 days. Gray micro-crystals were obtained after cooling to room temperature. Pure sample was obtained by filtering and washing the raw product with hot DMA/DMPU mixtures for 3-5 times. The yield was about 50% based on Fe.

Synthesis of [Co_3(OH)($DHBDC)_3$(TPT)] (CPM-171, Co3-DHBDC). In a 20 ml glass vial, 95.2 mg of $CoCl_2{\cdot}6H_2O$, 39.6 mg of DHBDC and 62.4 mg of TPT were dissolved in a mixture of 4.0 g of

DMA and 2.0 g of DMPU. After addition of 28 μL HFP, the vial was sealed and placed in a 130 ^{o}C oven for 3 days. Red hexagonal needle crystals were obtained after cooling to room temperature. Pure sample was obtained by filtering and washing the raw product with hot DMA/DMPU mixtures for 2 times. The yield was about 31% based on DHBDC.

Synthesis of [$Co_3(OH)(OHBDC)_3(TPT)$] (CPM-172, Co3-OHBDC). In a 20 ml glass vial, 95.2 mg of $CoCl_2·6H_2O$, 36.4 mg of OHBDC and 62.4 mg of TPT were dissolved in a mixture of 4.0 g of DMA and 2.0 g of DMPU. After addition of 28 μL HFP, the vial was sealed and placed in a 130 ^{o}C oven for 3 days. Red polyhedral crystals were obtained after cooling to room temperature. Pure sample was obtained by filtering and washing the raw product with hot DMA/DMPU mixtures for 2 times. The yield was about 35% based on OHBDC.

Synthesis of [$Co_3(OH)(BDC)_3(TPT)$] (CPM-173, Co3-BDC). In a 20 ml glass vial, 95.2 mg of $CoCl_2·6H_2O$, 33.2 mg of BDC and 62.4 mg of TPT were dissolved in a mixture of 4.0 g of DMA and 2.0 g of DMPU. After addition of 28 μL HFP, the vial was sealed and placed in a 130 ^{o}C oven for 3 days. Large red polyhedron crystals were obtained after cooling to room temperature. Pure sample was obtained by filtering and washing the raw product with hot DMA/DMPU mixtures for 2 times. The yield was about 46% based on BDC.

Synthesis of [$Co_3(OH)(NH_2BDC)_3(TPT)$] (CPM-174, Co3-NH_2BDC): In a 20 ml glass vial, 95.2 mg of $CoCl_2·6H_2O$, 36.2 mg of NH_2BDC and 62.4 mg of TPT were dissolved in a mixture of 4.0 g of DMA and 2.0 g of DMPU. After addition of 28 μL HFP, the vial was sealed and placed in a 130 ^{o}C oven for 3 days. Large red polyhedral crystals were obtained after cooling to room temperature. Pure sample was obtained by filtering and washing the raw product with hot DMA/DMPU mixtures for 2 times. The yield was about 48% based on NH_2BDC.

Synthesis of [$Ni_3(OH)(OHBDC)_3(TPT)$] (CPM-182, Ni3-OHBDC). In a 20 ml glass vial, 116.0 mg of $Ni(NO_3)_2·6H_2O$, 72.8 mg of OHBDC, 62.4 mg of TPT were dissolved in a mixture of 10.0 g of DMF, 2.0 g of CH_3CH_2OH and 2.0 g H_2O. The vial was sealed and placed in a 120 ^{o}C oven for 7 days. Pure green micro-crystals were obtained after cooling to room temperature. Pure sample was obtained by filtering and washing the raw product with DMF. The yield was about 55% based on Ni.

Synthesis of [$Ni_3(OH)(BDC)_3(TPT)$] (CPM-33a, Ni3-BDC) and **[$Ni_3(OH)(DHBDC)_3(TPT)$] (CPM-33b, Ni3-DHBDC).** CPM-33a and 33b were prepared according to our former reported procedures.

Synthesis of [$(CH_3)_2NH_2$][$Zn_3(OH)(OHBDC)_3(TPT)$] (CPM-192, Zn3-OHBDC). In a 20 ml glass

vial, 54.4 mg of $ZnCl_2$, 118.8 mg of $Zn(NO_3)_2{\cdot}6H_2O$, 36.4 mg of OHBDC, 62.4 mg of TPT were dissolved in a mixture of 4.0 g of DMA and 2.0 g of DMPU. After addition of 28 µL HFP, the vial was sealed and placed in a 130 ^oC oven for 5 days. Yellow crystals were obtained after cooling to room temperature. Pure sample was obtained by filtering and washing the raw product with hot DMA/DMPU mixtures for 2 times. The yield was about 36% based on OHBDC.

Synthesis of $[(CH_3)_2NH_2][Zn_3(OH)(BDC)_3(TPT)]$ (CPM-193, Zn3-BDC). In a 20 ml glass vial, 54.4 mg of $ZnCl_2$, 118.8 mg of $Zn(NO_3)_2{\cdot}6H_2O$, 33.2 mg of BDC, 62.4 mg of TPT were dissolved in a mixture of 4.0 g of DMA and 2.0 g of DMPU. After addition of 28 µL HFP, the vial was sealed and placed in a 130 ^oC oven for 3 days. Yellow irregular crystals were obtained after cooling to room temperature. Pure sample was obtained by filtering and washing the raw product with hot DMA/DMPU mixtures for 2 times. The yield was about 35% based on OHBDC.

Synthesis of $[(CH_3)_2NH_2][Zn_3(OH)(NH_2BDC)_3(TPT)]$ (CPM-194, Zn3-NH_2BDC). In a 20 ml glass vial, 54.4 mg of $ZnCl_2$, 118.8 mg of $Zn(NO_3)_2{\cdot}6H_2O$, 36.2 mg of NH_2BDC, 62.4 mg of TPT were dissolved in a mixture of 4.0 g of DMA and 2.0 g of DMPU. After addition of 28 µL HFP, the vial was sealed and placed in a 130 ^oC oven for 3 days. Large yellow polyhedral crystals were obtained after cooling to room temperature. Pure sample was obtained by filtering and washing the raw product with hot DMA/DMPU mixtures for 2 times. The yield was about 40% based on OHBDC.

Synthesis of $[Mg_2Sc(OH)(OHBDC)_3(TPT)]$ (CPM-212, Mg2Sc-OHBDC). In a 20 ml glass vial, 51.3 mg of $Mg(NO_3)_2{\cdot}6H_2O$, 23.1 mg of $Sc(NO_3)_3{\cdot}xH_2O$, 54.6 mg of OHBDC, 46.8 mg of TPT were dissolved in a mixture of 4.0 g of DMA and 2.0 g of DMPU. After addition of 0.5 g 85% formic acid, the vial was sealed and placed in a 130 ^oC oven for 5 days. Yellow micro-crystals were obtained after cooling to room temperature. Pure sample was obtained by filtering and washing the raw product with hot DMA/DMPU mixtures 3-5 times. The yield was about 45% based on Mg.

Synthesis of $[Mg_2Sc(OH)(BDC)_3(TPT)]$ (CPM-213, Mg2Sc-BDC). In a 20 ml glass vial, 51.3 mg of $Mg(NO_3)_2{\cdot}6H_2O$, 23.1 mg of $Sc(NO_3)_3{\cdot}xH_2O$, 33.2 mg of BDC, 31.2 mg of TPT were dissolved in a mixture of 4.0 g of DMA and 2.0 g of DMPU. After addition of 0.5 g 85% formic acid, the vial was sealed and placed in a 130 ^oC oven for 5 days. Gray micro-crystals were obtained after cooling to room temperature. Pure sample was obtained by filtering and washing the

raw product with hot DMA/DMPU mixtures 3-5 times. The yield was about 54% based on BDC.

Synthesis of [$Mg_2Ti(O)(OHBDC)_3(TPT)$] (CPM-222, Mg2Ti-OHBDC). In a 20 ml glass vial, 81.2 mg of $MgCl_2 \cdot 6H_2O$, 49.8 mg of $TiCp_2Cl_2$, 91.0 mg of OHBDC, 62.4 mg of TPT were dissolved in a mixture of 4.0 g of DMA and 2.0 g of DMPU. After addition of 0.5 g 85% formic acid, the vial was sealed and placed in a 130 ^oC oven for 5 days. Gray micro-crystals were obtained after cooling to room temperature. Pure sample was obtained by filtering and washing the raw product with hot DMA/DMPU mixtures 3-5 times. The yield was about 50% based on Mg.

Synthesis of [$Mg_2Ti(O)(BDC)_3(TPT)$] (CPM-223, Mg2Ti-BDC). In a 20 ml glass vial, 81.2 mg of $MgCl_2 \cdot 6H_2O$, 49.8 mg of $TiCp_2Cl_2$, 83.0 mg of BDC, 62.4 mg of TPT were dissolved in a mixture of 4.0 g of DMA and 2.0 g of DMPU. After addition of 0.5 g 85% formic acid, the vial was sealed and placed in a 130 ^oC oven for 5 days. Gray micro-crystals were obtained after cooling to room temperature. Pure sample was obtained by filtering and washing the raw product with hot DMA/DMPU mixtures 3-5 times. The yield was about 58% based on Mg.

Synthesis of [$Mg_2V(OH)(DHBDC)_3(H_2O)_3$] (CPM-230, Mg2V-MIL-88). In a 20 ml glass vial, 81.2 mg of $MgCl_2 \cdot 6H_2O$, 31.5 mg of VCl_3, and 99.0 mg of DHBDC were dissolved in a mixture of 4.0 g of DMA and 2.0 g of DMPU. After addition of 0.3 g 85% formic acid, the vial was sealed and placed in a 100 ^oC oven for 5 days. Large pure brown crystals were obtained after cooling to room temperature. Pure sample was obtained by filtering and washing the raw product with DMA. The yield was about 55% based on Mg.

Synthesis of [$Mg_2V(OH)(DHBDC)_3(TPT)$] (CPM-231, Mg2V-DHBDC). In a 20 ml glass vial, 81.2 mg of $MgCl_2 \cdot 6H_2O$, 31.5 mg of VCl_3, 99.0 mg of DHBDC, 62.4 mg of TPT were dissolved in a mixture of 4.0 g of DMA and 2.0 g of DMPU. After addition of 0.3 g 85% formic acid, the vial was sealed and placed in a 130 ^oC oven for 5 days. The brown crystals of CPM-231 crystallized on the walls of the glass vial after cooling to room temperature. The black solid on the bottom of the vial was discarded. Pure crystals on the walls of the vial were obtained by filtering and washing the raw product with DMA. The yield was about 20% based on Mg.

Synthesis of [$Mg_2V(OH)(OHBDC)_3(TPT)$] (CPM-232, Mg2V-OHBDC). In a 20 ml glass vial, 81.2 mg of $MgCl_2 \cdot 6H_2O$, 31.5 mg of VCl_3, 91.0 mg of OHBDC, 62.4 mg of TPT were dissolved in a mixture of 4.0 g of DMA and 2.0 g of DMPU. After addition of 0.3 g 85% formic acid, the vial was sealed and placed in a 130 ^oC oven for 5 days. Pure brown micro-crystals were obtained

after cooling to room temperature. Pure sample was obtained by filtering and washing the raw product with DMA. The yield was about 35% based on Mg.

Synthesis of [$Mg_2V(OH)(BDC)_3(TPT)$] (CPM-233, Mg2V-BDC). In a 20 ml glass vial, 81.2 mg of $MgCl_2·6H_2O$, 31.5 mg of VCl_3, 83.0 mg of BDC, 62.4 mg of TPT were dissolved in a mixture of 4.0 g of DMA and 2.0 g of DMPU. After addition of 0.3 g 85% formic acid, the vial was sealed and placed in a 130 ^oC oven for 5 days. Pure gray micro-crystals were obtained after cooling to room temperature. Pure sample was obtained by filtering and washing the raw product with DMA. The yield was about 58% based on Mg.

Synthesis of [$Mg_2V(OH)(NH_2BDC)_3(TPT)$] (CPM-234, Mg2V-$NH_2$BDC). In a 20 ml glass vial, 81.2 mg of $MgCl_2·6H_2O$, 31.5 mg of VCl_3, 90.5 mg of NH_2BDC, 62.4 mg of TPT were dissolved in a mixture of 4.0 g of DMA and 2.0 g of DMPU. After addition of 0.3 g 85% formic acid, the vial was sealed and placed in a 130 ^oC oven for 5 days. Pure gray micro-crystals were obtained after cooling to room temperature. Pure sample was obtained by filtering and washing the raw product with DMA. The yield was about 54% based on Mg.

Synthesis of [$Mg_2V(OH)(NO_2BDC)_3(TPT)$] (CPM-235, Mg2V-$NO_2$BDC). In a 20 ml glass vial, 81.2 mg of $MgCl_2·6H_2O$, 31.5 mg of VCl_3, 105.5 mg of NO_2BDC, 62.4 mg of TPT were dissolved in a mixture of 4.0 g of DMA and 2.0 g of DMPU. After addition of 0.3 g 85% formic acid, the vial was sealed and placed in a 120 ^oC oven for 5 days. Large pure brown crystals were obtained after cooling to room temperature. Pure sample was obtained by filtering and washing the raw product with DMA. The yield was about 65% based on Mg.

Synthesis of [$Mg_2V(OH)(14NDC)_3(TPT)$] (CPM-236, Mg2V-14NDC). In a 20 ml glass vial, 81.2 mg of $MgCl_2·6H_2O$, 31.5 mg of VCl_3, 108.1 mg of 14NDC, 62.4 mg of TPT were dissolved in a mixture of 4.0 g of DMA and 2.0 g of DMPU. After addition of 0.3 g 85% formic acid, the vial was sealed and placed in a 120 ^oC oven for 5 days. Large pure brown crystals were obtained after cooling to room temperature. Pure sample was obtained by filtering and washing the raw product with DMA. The yield was about 56% based on Mg.

Synthesis of [$Mg_2V(OH)(26NDC)_3(TPT)$] (CPM-237, Mg2V-26NDC). In a 20 ml glass vial, 81.2 mg of $MgCl_2·6H_2O$, 31.5 mg of VCl_3, 108.1 mg of 26NDC, 62.4 mg of TPT were dissolved in a mixture of 4.0 g of DMA and 2.0 g of DMPU. After addition of 0.3 g 85% formic acid, the vial was sealed and placed in a 120 ^oC oven for 5 days. Large pure brown crystals were obtained after cooling to room temperature. Pure sample was obtained by filtering and washing the raw product with DMA. The yield was about 62% based on Mg.

Synthesis of [$Mg_2Fe(OH)(DHBDC)_3(TPT)$] (CPM-261, Mg2Fe-DHBDC). In a 20 ml glass vial, 81.2 mg of $MgCl_2 \cdot 6H_2O$, 54.0 mg of $FeCl_3 \cdot 6H_2O$, 99.0 mg of DHBDC, 62.4 mg of TPT were dissolved in a mixture of 4.0 g of DMA and 2.0 g of DMPU. After addition of 0.5 g 85% formic acid, the vial was sealed and placed in a 130 ^oC oven for 5 days. Dark brown micro-crystals were obtained after cooling to room temperature. Pure sample was obtained by filtering and washing the raw product with hot DMA/DMPU mixtures 3-5 times. The yield was about 66% based on Mg.

Synthesis of [$Mg_2Fe(OH)(OHBDC)_3(TPT)$] (CPM-262, Mg2Fe-OHBDC). In a 20 ml glass vial, 81.2 mg of $MgCl_2 \cdot 6H_2O$, 54.0 mg of $FeCl_3 \cdot 6H_2O$, 91.0 mg of OHBDC, 62.4 mg of TPT were dissolved in a mixture of 4.0 g of DMA and 2.0 g of DMPU. After addition of 0.5 g 85% formic acid, the vial was sealed and placed in a 130 ^oC oven for 5 days. Brown micro-crystals were obtained after cooling to room temperature. Pure sample was obtained by filtering and washing the raw product with hot DMA/DMPU mixtures 3-5 times. The yield was about 60% based on Mg.

Synthesis of [$Mg_2Fe(OH)(BDC)_3(TPT)$] (CPM-263, Mg2Fe-BDC). In a 20 ml glass vial, 81.2 mg of $MgCl_2 \cdot 6H_2O$, 54.0 mg of $FeCl_3 \cdot 6H_2O$, 83.0 mg of BDC, 62.4 mg of TPT were dissolved in a mixture of 4.0 g of DMA and 2.0 g of DMPU. After addition of 0.5 g 85% formic acid, the vial was sealed and placed in a 130 ^oC oven for 5 days. Brown micro-crystals were obtained after cooling to room temperature. Pure sample was obtained by filtering and washing the raw product with hot DMA/DMPU mixtures 3-5 times. The yield was about 56% based on Mg.

Synthesis of [$Mg_2Ga(OH)(DHBDC)_3(TPT)$] (CPM-271, Mg2Ga-DHBDC). In a 20 ml glass vial, 81.2 mg of $MgCl_2 \cdot 6H_2O$, 51.2 mg of $Ga(NO_3)_3 \cdot xH_2O$, 99.0 mg of DHBDC, 62.4 mg of TPT were dissolved in a mixture of 4.0 g of DMA and 2.0 g of DMPU. After addition of 0.5 g 85% formic acid, the vial was sealed and placed in a 130 ^oC oven for 5 days. Yellow micro-crystals were obtained after cooling to room temperature. Pure sample was obtained by filtering and washing the raw product with hot DMA/DMPU mixtures 3-5 times. The yield was about 48% based on Mg.

Synthesis of [$Mg_2Ga(OH)(OHBDC)_3(TPT)$] (CPM-272, Mg2Ga-OHBDC). In a 20 ml glass vial, 81.2 mg of $MgCl_2 \cdot 6H_2O$, 51.2 mg of $Ga(NO_3)_3 \cdot xH_2O$, 91.0 mg of OHBDC, 62.4 mg of TPT were dissolved in a mixture of 4.0 g of DMA and 2.0 g of DMPU. After addition of 0.5 g 85% formic acid, the vial was sealed and placed in a 130 ^oC oven for 5 days. Yellow micro-crystals were obtained after cooling to room temperature. Pure sample was obtained by filtering and

washing the raw product with hot DMA/DMPU mixtures 3-5 times. The yield was about 49% based on Mg.

Synthesis of [$Mg_2Ga(OH)(BDC)_3(TPT)$] (CPM-273, Mg2Ga-BDC). In a 20 ml glass vial, 81.2 mg of $MgCl_2 \cdot 6H_2O$, 51.2 mg of $Ga(NO_3)_3 \cdot xH_2O$, 83.0 mg of BDC, 62.4 mg of TPT were dissolved in a mixture of 4.0 g of DMA and 2.0 g of DMPU. After addition of 0.5 g 85% formic acid, the vial was sealed and placed in a 130 ^oC oven for 5 days. Gray micro-crystals were obtained after cooling to room temperature. Pure sample was obtained by filtering and washing the raw product with hot DMA/DMPU mixtures 3-5 times. The yield was about 53% based on Mg.

Synthesis of [$Mg_2In(OH)(DHBDC)_3(TPT)$] (CPM-291, Mg2In-DHBDC). In a 20 ml glass vial, 81.2 mg of $MgCl_2 \cdot 6H_2O$, 44.2 mg of $InCl_3$, 99.0 mg of DHBDC, 62.4 mg of TPT were dissolved in a mixture of 4.0 g of DMA and 2.0 g of DMPU. After addition of 0.5 g 85% formic acid, the vial was sealed and placed in a 130 ^oC oven for 5 days. Brown micro-crystals were obtained after cooling to room temperature. Pure sample was obtained by filtering and washing the raw product with hot DMA/DMPU mixtures 3-5 times. The yield was about 41% based on Mg.

Synthesis of [$Mg_2In(OH)(OHBDC)_3(TPT)$] (CPM-292, Mg2In-OHBDC). In a 20 ml glass vial, 102.6 mg of $Mg(NO_3)_2 \cdot 6H_2O$, 60.0 mg of $In(NO_3)_3 \cdot xH_2O$, 91.0 mg of OHBDC, 62.4 mg of TPT were dissolved in a mixture of 4.0 g of DMA and 2.0 g of DMPU. After addition of 0.5 g 85% formic acid, the vial was sealed and placed in a 130 ^oC oven for 5 days. Yellow micro-crystals were obtained after cooling to room temperature. Pure sample was obtained by filtering and washing the raw product with hot DMA/DMPU mixtures 3-5 times. The yield was about 47% based on Mg.

Synthesis of [$Mg_2In(OH)(BDC)_3(TPT)$] (CPM-293, Mg2In-BDC). In a 20 ml glass vial, 102.6 mg of $Mg(NO_3)_2 \cdot 6H_2O$, 60.0 mg of $In(NO_3)_3 \cdot xH_2O$, 83.0 mg of BDC, 62.4 mg of TPT were dissolved in a mixture of 4.0 g of DMA and 2.0 g of DMPU. After addition of 0.5 g 85% formic acid, the vial was sealed and placed in a 130 ^oC oven for 5 days. Gray micro-crystals were obtained after cooling to room temperature. Pure sample was obtained by filtering and washing the raw product with hot DMA/DMPU mixtures 3-5 times. The yield was about 49% based on Mg.

Single crystal X-ray diffraction. The single crystal samples of **Mg3-MIL-88 (CPM-140)**,

Mg3-DHBDC (CPM-141), **Mg3-OHBDC (CPM-142)**, **Mg3-BDC (CPM-143)**, **Mg3-NH_2BDC (CPM-144)**, **Mn3-BDC (CPM-153)**, **Co3-NH_2BDC (CPM-174)**, **Zn3-NH_2BDC (CPM-194)**, **Mg2V-MIL-88 (CPM-230)**, **Mg2V-DHBDC (CPM-231)**, **Mg2V-NO_2BDC (CPM-235)**, **Mg2V-14NDC (CPM-236)**, and **Mg2V-26NDC (CPM-237)** are suitable for the single-crystal X-ray analysis, which was performed on a Bruker Smart APEX II CCD area diffractometer with nitrogen-flow temperature controller using graphite-monochromated MoKα radiation (λ = 0.71073 Å), operating in the ω and φ scan mode. The SADABS program was used for absorption correction. The structure was solved by direct methods and refined using SHELXTL[15]. All non-hydrogen atoms in the framework were refined with anisotropic displacement parameters. The Mg/V ratio was estimated from the occupancy refinement with single crystal X-ray diffraction data and further supported by the EDX analysis. The large volume fractions of solvents in the lattice pores could not be modelled in terms of atomic sites and were treated using the SQUEEZE routine in the PLATON software package[16]. Crystal data as well as details of data collection and refinements were summarized in Tables S9-S15.

Powder X-ray diffraction. Powder X-ray diffraction (XRD) data were collected on a Bruker D8 Advance powder diffractionmeter with CuKα radiation (40 kV, 40 mA, λ = 1.5418 Å). The simulated powder pattern was calculated using single-crystal X-ray diffraction data and processed by the Mercury 2.3 program provided by the Cambridge Crystallographic Data Centre.

Energy dispersive spectroscopy (EDS). The semi-quantitative elemental analyses of different heterometallic MOF samples (Mg2Sc, Mg2Ti, Mg2V, Mg2Fe, Mg2Ga, and Mg2In) were performed by using a Philips FEI XL30 field emission scanning electron microscope (FESEM) equipped with PGT-IMIX PTS energy dispersive spectroscopy (EDS) detector. Data acquisition was performed with an accelerating voltage of 20 kV and 60 s accumulation time.

Gas adsorption. Gas sorption isotherms of all reported MOFs were measured on a Micromeritics ASAP 2020M surface-area and pore-size analyzer up to 1 atm of gas pressure by the static volumetric method. The as-synthesized samples of **Mg-MIL-88 (CPM-140)** and **Mg2V-MIL-88 (CPM-230)** were immersed in CH_2Cl_2 for 3 days; during the exchange, the CH_2Cl_2 was refreshed three times. The resulting CH_2Cl_2-exchanged samples were then

evacuated (10^{-3} torr) at 60 ^{o}C for 12 h. To activate the crystal samples of all other **[M$_3$(OH/O)(dicarboxylate)$_3$(TPT)]** samples, CH_3OH was used instead of CH_2Cl_2. The resulting CH_3OH-exchanged samples were then evacuated (10^{-3} torr) at 80 ^{o}C for 12 h. All gases used were of 99.999% purity. The gas sorption isotherms for N_2 and H_2 were measured at 77 K. The gas sorption isotherms for CO_2, CH_4 and C_2H_2 were measured at 273 K or 298 K. The isosteric heat of CO_2 adsorption was estimated from the CO_2 sorption data measured at 273 K and 298 K, by using a virial-type expression.

Isosteric heats of adsorption. The isosteric heats of adsorption (Q_{st}) are obtained from the CO_2 sorption isotherms at 273 K and 298 K using Clausius–Clapeyron equation.

$$Q_{st} = R\ \{[\partial \ln p]/[\partial\ (1/T)]\}q \qquad (1)$$

where R is the universal gas constant, q is the amount of CO_2 loaded at pressure p and temperature T. These calculations are done through the "Heat of Adsorption" calculated function embedded in the software supplied by Micromeritics ASAP 2020M surface-area and pore-size analyzer machine.

Selectivity prediction for binary mixture adsorption. Ideal adsorbed solution theory (IAST) was used to predict binary mixture adsorption[17,18] from the experimental pure-gas isotherms. To perform the integrations required by IAST, the single-component isotherms should be fitted by a proper model. There is no restriction on the choice of the model to fit the adsorption isotherms, however, data over the pressure range under study should be fitted precisely [19,20]. Several isotherm models were tested to fit the experimental pure isotherms for CH_4 and CO_2, and the Langmuir–Freundlich equation was found to be the best fit to the experimental data:

$$y = q*b*x^{(1/t)}/(1+b*x^{(1/t)}) \qquad (2)$$

Here, q is the adsorbed amount per mass of adsorbent (mol/kg), x is the pressure of the bulk gas at equilibrium with the adsorbed phase (kPa), b is the affinity coefficients of the sites (1/kPa), and t is measures of the deviations from an ideal homogeneous surface. The R values for all of the fitted isotherms were over 0.99. Hence, the fitted isotherm parameters were applied to perform the necessary integrations in IAST.

Breakthrough curves. The gas-separation properties of **Mg2V-DHBDC (CPM-231)** and **Mg2V-OHBDC (CPM-232)** were also examined by breakthrough experiments using CO_2:CH_4 gas mixtures (about 0.15:0.85 v/v) flowed through activated samples of **CPM-231** (250 mg) and **CPM-232** (400 mg) packed into a glass column (0.3 cm inner diameter, 8 cm length for **CPM-231** and 15 cm length for **CPM-232**). Argon gas was initially purged into the sample column. The gas mixture was dosed into the column at a flow rate of 16 mL min^{-1}. The relative amounts of the gases passing through the column were monitored on a mass spectrometer gas analysis system (Pfeiffer vacuum) detecting ion peaks at m/z 44 (CO_2) and 16 (CH_4).

Density function theory (DFT) calculations. Density function theory (DFT) calculations were performed using CASTEP [21]. The Generalized Gradient Approximation (GGA), as implemented by Perdew-Burke-Ernzerhof (PBE), was used to describe the exchange-correlation interactions. Norm-conserving pseudopotentials were employed to account for the effects of core electrons. The unit cell configuration determined by XRD was used as the initial structure for the simulations. Hydrogen atoms that cannot be determined in XRD are added according to the nominal bond length and angle. Some of the sites have partial occupancy, and to account for this properly a supercell calculation would be desirable, but too costly in practice. Instead, a single unit cell was used and the partially occupied sites were modified to be either occupied or unoccupied so that the overall probability of being occupied is proportional to the actual occupancy. The atomic coordinates were relaxed with no imposed symmetry to allow minimization of the potential energy and the interatomic forces. The energy tolerance for the electronic structure calculations was 5×10^{-7} eV, and the energy tolerance for ionic relaxation was 5×10^{-6} eV. The tolerance for the interatomic forces was 0.01 eV/Å. After convergence was reached, the dynamical matrix was obtained using the linear response method, from which the phonon frequencies and vibrational modes were calculated. The electronic structure calculations and the phonon calculations were performed at the gamma-point only. The aClimax software[22] was used to convert the DFT-calculated phonon results to the simulated INS spectra.

Supplementary References

1. Liao, P., Chen, H., Zhou, D., Liu, S., He, C., Rui, Z., Ji, H., Zhang, J. & Chen, X. Monodentate hydroxide as a super strong yet reversible active site for CO_2 capture from high-humidity flue gas, *Energy Environ. Sci.* **8,** 1011-1016 (2015).
2. Zheng, B., Bai, J., Duan, J., Wojtas, L. & Zaworotko, M. J. Enhanced CO_2 binding affinity of a high-uptake *rht*-type metal−organic framework decorated with acylamide groups. *J. Am. Chem. Soc.* **133,** 748-751 (2011).
3. Liu, B., Yao, S., Shi, C., Li, G., Huo, Q. & Liu, Y. Significant enhancement of gas uptake capacity and selectivity via the judicious increase of open metal sites and Lewis basic sites within two polyhedron-based metal-organic frameworks. *Chem. Commun.* **52,** 3223-3226 (2016).
4. Lu, Z., Bai, J., Hang, C., Meng, F., Liu, W., Pan, Y. & You, X. The utilization of amide groups to expand and functionalize metal-organic frameworks simultaneously. *Chem. Eur. J.* **22,** 6277-6285 (2016).
5. Lassig, D., Lincke, J., Moellmer, J., Reichenbach, C., Moeller, A., Glaser, R., Kalies, G., Cychosz, K. A., Thommes, M., Staudt, R. & Krautscheid, H. A microporous copper metal-organic framework with high H_2 and CO_2 adsorption capacity at ambient pressure. *Angew. Chem., Int. Ed.* **50,** 10344-10348 (2011).
6. Alsmail, N. H., Suyetin, M., Yan, Y., Cabot, R., Krap, C. P., Lü, J., Easun, T. L., Bichoutskaia, E., Lewis, W., Blake, A. J. & Schröder, M. Analysis of high and selective uptake of CO_2 in an oxamide-containing $\{Cu_2(OOCR)_4\}$-based metal-organic framework. *Chem. Eur. J.* **20,** 7317-7324 (2014).
7. Spanopoulos, I., Bratsos, I., Tampaxis, C., Vourloumis, D., Klontzas, E., Froudakis, G. E., Charalambopoulou, G., Steriotisb, T. A. & Trikalitis, P. N. Exceptional gravimetric and volumetric CO_2 uptake in a palladated NbO-type MOF utilizing cooperative acidic and basic, metal–CO_2 interactions. *Chem. Commun.* **52,** 10559-10562 (2016).
8. De, D., Pal, T. K., Neogi, S., Senthilkumar, S., Das, D., Gupta, S. S. & Bharadwaj, P. K. A versatile Cu^{II} metal–organic framework exhibiting high gas storage capacity with selectivity for CO_2: conversion of CO_2 to cyclic carbonate and other catalytic abilities. *Chem. Eur. J.* **22,** 3387-3396 (2016).
9. Lee, Y., Moon, H. R., Cheon, Y. E. & Suh, M. P. A comparison of the H_2 sorption capacities of isostructural metal-organic frameworks with and without accessible metal sites: $[\{Zn_2(abtc)(dmf)_2\}_3]$ and $[\{Cu_2(abtc)(dmf)_2\}_3]$ versus $[\{Cu_2(abtc)\}_3]$. *Angew. Chem. Int. Ed.* **47,** 7741-7745 (2008).
10. Li, J. R., Yu, J., Lu, W., Sun, L. B., Sculley, J., Balbuena, P. B. & Zhou, H. C. Porous materials with

pre-designed single-molecule traps for CO_2 selective adsorption. *Nat. Commun.* **4,** 1538 (2013).

11. Luebke, R., Eubank, J. F., Cairns, A. J., Belmabkhout, Y., Wojtas, L. & Eddaoudi, M. The unique rht-MOF platform, ideal for pinpointing the functionalization and CO_2 adsorption relationship. *Chem. Commun.* **48,** 1455-1457 (2012).
12. Nugent, P., Belmabkhout, Y., Burd, S. D., Cairns, A. J., Luebke, R., Forrest, K., Pham, T., Ma, S., Space, B., Wojtas, L., Eddaoudi, M. & Zaworotko, M. J. Porous materials with optimal adsorption thermodynamics and kinetics for CO_2 separation. *Nature* **495,** 80-84 (2013).
13. Lin, R. B., Chen, D., Lin, Y. Y., Zhang, J. P. & Chen, X. M. A zeolite-like zinc triazolate framework with high gas adsorption and separation performance. *Inorg. Chem.* **51,** 9950-9955 (2012).
14. An, J., Geib, S. J. & Rosi, N. L. High and selective CO_2 uptake in a cobalt adeninate metal-organic framework exhibiting pyrimidine- and amino-decorated pores. *J. Am. Chem. Soc.* **132,** 38-39 (2009).
15. Sheldrick, G. M. A short history of *SHELX*. *Acta Crystallogr. A* **64,** 112-122 (2008).
16. Spek, A. L. Single-crystal structure validation with the program *PLATON. J. Appl. Crystallogr.* **36,** 7-13 (2003).
17. Bae, Y. S., Mulfort, K. L., Frost, H., Ryan, P., Punnathanam, S., Broadbelt, L. J., Hupp, J. T. & Snurr, R. Q. Separation of CO_2 from CH_4 using mixed-ligand metal–organic frameworks. *Langmuir* **24,** 8592-8598 (2008).
18. Cessford, N. F., Seaton, N. A. & Duren, T. Evaluation of ideal adsorbed solution theory as a tool for the design of metal-organic framework materials. *Ind. Eng. Chem. Res.* **51,** 4911-4921 (2012).
19. Babarao, R., Hu, Z. Q., Jiang, J. W., Chempath, S. & Sandler, S. I. Storage and separation of CO_2 and CH_4 in silicalite, C168 schwarzite, and IRMOF-1: a comparative study from Monte Carlo simulation. *Langmuir* **23,** 659-666 (2007).
20. Goetz, V., Pupier, O. & Guillot, A. Carbon dioxide-methane mixture adsorption on activated carbon. *Adsorption* **12,** 55-63 (2006).
21. Clark, S. J., Segall, M. D., Pickard, C. J., Hasnip, P. J., Probert, M. J., Refson, K. & Payne, M. C. First principles methods using CASTEP. *Zeitschrift für Kristallographie* **220,** 567-571 (2005).
22. A. J. Ramirez-Cuesta, aCLIMAX 4.0.1, The new version of the software for analyzing and interpreting INS spectra. *Comput. Phys. Commun.* **157,** 226-238 (2004).
